# Contributions to the development and simulations of generic, modular and multiphysics greenhouses dynamic models, evaluated with a whole year study case dataset

**DOI:** 10.1371/journal.pone.0340619

**Published:** 2026-02-17

**Authors:** Samuel Sourisseau, Cyril Toublanc, Etienne Chantoiseau, Michel Havet

**Affiliations:** 1 Oniris, Nantes Université, CNRS, GEPEA, UMR 6144, UsC INRAE, F-44000, Nantes, France; 2 Institut Agro Rennes-Angers UP EPHor Environmental Physics and Horticulture Research Unit, F-49045, Angers, France; University of Bologna, ITALY

## Abstract

North-Western Europe heated greenhouses need to address their high fossil energy dependency while they start to face with climate change. In this context, physics-based greenhouses dynamic models can be used for prospective assessments of innovative shapes, equipment, control, etc. A prerequisite for such model-based evaluation is a review of existing implementations, the improvement and development of suitable sub-models in a generic and modular approach that do not require calibration, and the evaluation of a use case global model with a solid experimental dataset. First, this paper details contributions to existing models regarding several aspects: solar gain, boundaries effects, airflows and leakages, heat and mass transfer. The second part is dedicated to the evaluation of an experimental tomato greenhouse global model for an 11-month period. Its assessment is multiphysics: indoor climate, utilities consumptions, yield and Leaf Area Index. The resulting 5 min sampling indoor air climate Root Mean Square Error is 1.3 °C (temperature) and 8.1%RH (relative humidity). The tomato yield Mean Absolute Error is 1.1 kg m^-2^. Mass balances also quantify the losses and potentials for water and CO_2_. The global model outputs are compared with literature, and it is demonstrated that assessing the accuracy of models based only on statistical indicators is questionable. This approach is compatible with assessments of prospective solutions, it increases the confidence for scaling results from small to large commercial greenhouses, and it constitutes a base from which simpler and black box models can be derived for other applications such as predictive control.

## 1. Introduction

Compared with open field cultivation and at equivalent land use, greenhouses constitute a widespread way to increase yield and to extend the production period for vegetables and fruits that can grow under shelter. Acting as a barrier with outdoor, they protect the crop from weather hazards and possible external biotic threats such as insects [1] which may also be vectors of diseases [2]. Conversely, it makes the use of integrated pest management easier [3]. Depending on the greenhouse properties and associated equipment, the indoor growth environment can also be adapted to provide better conditions and to avoid those favorable to physiological disorders or diseases development [4,5].

During the past decades, the continuous evolutions applied to greenhouse cultivation led to considerable effects on fruits and vegetables production. In the Netherlands for instance, the yields have been increased by 113% for tomato between 1983 and 2010 [6]. Nevertheless, the Intergovernmental Panel on Climate Change has identified with high confidence a key risk for the European agriculture; without adaptation, expected production losses in the Southern part due to heat and drought would not be balanced by the Northern countries [7]. On the other side, the Northern Europe greenhouses agronomic performance partially results from a high fossil-energy consumption when heating is applied [8], and is consequently not sustainable. Thus, cultivation practices have to evolve in the concerned areas.

In France, the vegetables heated greenhouses are dedicated to tomato (89%) and cucumber (11%), with an average energy consumption around 341 kWh m⁻² y⁻¹  corresponding to an energy efficiency of 5.7 kWh kg_tomato_^-1^ [9]. 80% of their surfaces depend on natural gas as a primary heat source because of the cogeneration facilities generalization in the last decades. In addition to this unsustainable high amount of energy consumption, heated greenhouses like any open field irrigated crop rely on water availability, although in a lower proportion because of a higher water efficiency usage [3,9,10]. Nevertheless, recurrent difficulties in water resource management nowadays occur in France, leading to water usage restrictions [11]. Predictions for the future are not optimistic: for instance [12], expect a decrease of precipitation during summer between 16% to 23% and a decrease of yearly average river flow between 10% to 40% (depending on the location) as well as some important low-water flow drops. As well, producers are also interested in evaluating the effects on the yield of more frequent and severe heat waves.

Concurrently to on-site experiments, solutions to these problems can be numerically assessed using models focused on simulating innovative architectures, materials, control, etc. However, because phenomena in greenhouses are highly coupled, they cannot be individually evaluated and a systemic approach must be used. As a matter of fact, greenhouse modelling is commonly used for indoor climate and energy studies since the 1950 [13]. From the 1980’s, the literature has been so prolific that is it now difficult to have a comprehensive understanding of all of them. According to [14], 41 greenhouses models have been developed between 1958–1986, while recent reviews count more than 150 models among which almost half of them have been created during the last decade. One of the objectives of their paper is precisely to provide the keys to identify the existing approaches or models that can be re-used or serve as a base for a defined purpose.

Among the existing model categories in the literature, the phenomenological analytical models aim at implementing physic-based equations at the suitable scale with reasonable simplifying assumptions. According to [15,16] was one of the first authors to implement a complete mechanistic dynamic greenhouse climate model. Many other detailed models of the same type have been developed since the 1990, such as the KASPRO one from [17] and the Gembloux Greenhouse Dynamic Model (GGDM) [18]. More recently [19], developed and evaluated an adaptive greenhouse one applicable to various climates for model-based design.

Concomitantly, the performance of standard computers has increased, offering the possibility to apply more and more complex approaches. Depending on the model type and purpose, a great diversity of tools and languages are now commonly used in the literature [20], such as Modelica [21,22]. Recently [23,24], implemented in this language the Vanthoor’s greenhouse models including the tomato yield one [25] as well as additional thermal and energy systems. The so-called “Greenhouses” library has been then re-used for several purposes:

- [26] built and calibrated a Canadian Northern greenhouse model and used it for the evaluation of a thermal energy storage system
- [27] explored growing scenarios at 10 locations in the U.S.A. using the two global greenhouse models embedded in the library
- [28] compared (simulation) the production of tomato in greenhouse and open-field to evaluate their footprints in terms of energy, gas and water.
- [29] adapted one of the embedded models for a multi-energy distributed control study

The modelling approach applied for the work presented in this paper uses the “Greenhouses” library as a start. From the understanding of the authors, no external code review was done in the previously cited studies, except [26] who did it on some sub-models he used: consequently, a comprehensive one has been firstly conducted. Then, further developments have been done not only to implement the components specifically needed for the present work, but also with the aim of applying a generic and modular methodology which does not imply noticeable model calibration. The latter requirement is justified by the fact that it should be possible to re-use the implemented sub-models to evaluate innovative greenhouses, where parameters can only be set according defined characteristics (dimensions, physical properties and equipment datasheets) or reasonable assumptions. Furthermore, a deeper integration of meteorological conditions appeared necessary. Indeed, 1) the light availability to the crop shall be handled with care, and greenhouse structures are by nature very sensitive to solar gain 2) while the availability of meteorological data as model inputs could be a problem twenty years ago, weather measurements in many locations in the world are now commonly accessible 3) there is a need to forecast the effect of the climate change in the models. Moreover, models in the literature are usually applied to large greenhouses, where boundary effects can be neglected. However, this causes some difficulties when such models are applied to small ones, where walls surfaces are noticeable compared to the roof. This should be taken into account (or at least quantified) to scale-up any results obtained in small (experimental) greenhouse to commercial ones. Besides, smaller greenhouses would be required for studies dedicated to the evaluation of protected cultivation integration in cities (urban farming). Finally, the modelling approach should also rely on methodology applied in other fields such as in buildings and control, facilitating the compatibility with available and detailed off-the-shelf implementations.

For clarity purpose, the modelling tools, the validation use-case experimental greenhouse and its extended dataset are presented first (section 1). A description of the contributions to the existing models and approaches in the literature follows in section 3. Finally, this paper presents the evaluation of a 1037 m^2^ experimental Venlo-type tomato greenhouse global model over the whole production season using an extended dataset (section 4).

Regarding the approach validation on the experimental greenhouse, the global model assessment has been done using various recorded data: indoor and outdoor climate, utilities consumptions, yield and Leaf Area Index (LAI, total leaves area divided by the cultivated floor area) measurements. Its parametrization is detailed in the Supporting Information S1 File. As for the Vanthoor’s tomato yield model parameters values, they have been adapted to correspond to the cultivar and cultivation practices: thus, it constitutes an instantiable reference for any future greenhouse concept evaluation. In the results part, a focus is done on the outputs of the sub-models dedicated to the roof solar transmittance calculation. The main ones (indoor climate, yield, LAI) are compared with measurements and with the literature. Discussions are also centered on mass transfer, model limits and “accuracy” assessment: in particular, the effects of more or less detailed approaches for the modelling of specific aspects is analyzed. As well, improvement perspectives are identified.

## 2. Materials and methods

### 2.1. Numerical tools

This systemic approach lies on the use of the open-specified Modelica language, which is dedicated to the modelling of multi-physics systems and aims to provide a suitable framework for knowledge-based implementation and to facilitate their sharing [30]. Its formalism includes convenient features such as acausality, units checking and all the advantages of object-oriented languages (inheritance, abstraction, etc.) [31]. Being domain-agnostic, it has been applied since more than two decades to various application fields [32] and numerous free and commercial libraries are developed, maintained, documented and shared. The present work used the “Greenhouses” v.1 [23] one as a starting point. However, others Free and Open-Source (FOS) libraries have also been explored, such as “Buildings” [33] from which a few sub-models have been re-used or slightly adapted. For future applications, the integration of domain-specific packages has also been anticipated (e.g., control and photovoltaic). The implementation was done based on Modelica v.4.0.0, and mainly using the Dymola v.2021x [34] simulation environment. Nevertheless, attention has been paid to ensure the compatibility with the FOS OpenModelica software [35] (see Supporting Information S5 File). All the results presented in this paper have been obtained with the DASSL [36] solver, although others such as the CVODE one [37] have also been tested.

Specific developments have also been done in Python (v. 3.8–3.12) for several purposes:

- Trivial model outputs visualization
- Processing of specific sub-models outputs, such as to visualize the radiance distributions provided by the implemented Modelica model detailed in Section 3.2.2, and of which the figure given in the latter Section is an example. Since this data post-processing is not trivial, the associated Python script is given in the Supporting Information S2 File.
- In addition to phenomena modelling and a literature review, calibration-free approaches may require preliminary studies for the assessment of some model inputs. In the present case, concerning radiative heat transfers, a specific methodology has been set up in Python to compute the view factors in close space with numerous obstacles (see section 3.5.2).

### 2.2. Experimental greenhouse description

**Fig 1:**
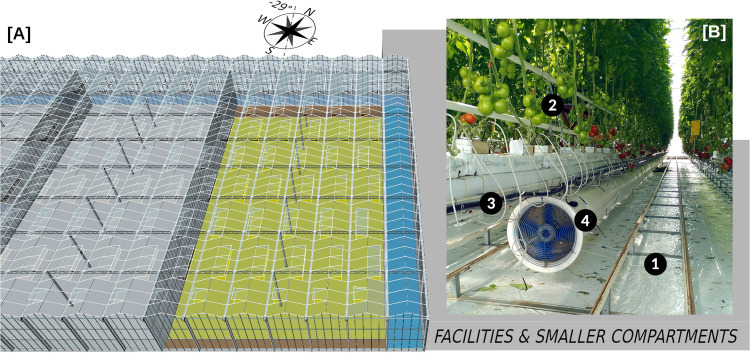
A studied greenhouse compartment general drawing and a picture from inside. (A) The studied greenhouse compartment (yellow) with its alleyways (brown), its adjoining corridors on the east and north sides (blue), and another similar compartment on its west side. (B) Picture taken in the compartment. In addition to three heating pipe networks (Rails 51 (1), Forcas (2), PE (3)), a perforated air mixing duct (4) is installed below 4 out of the 15 tomato rows.

As illustrated on Fig 1, the experimental glasshouse is a 1037 m^2^ Venlo-type compartment located at the CTIFL (*Centre Technique Interprofessionnel des Fruits et Légumes*) facilities close to Nantes in France (lat. 47.29°, long. −1.46°) in a sub-urban area. The greenhouse was built in 2013. The compartment is 43.2 m long, 24 m wide and 7.78 m high (6.97 m high below gutters). It is made of 6 spans of 4 m width and 22° roof slope (*ψ*_*r*_), and its orientation is not exactly North-South (−29°). It is surrounded on its north and east sides by corridors and on its west wall by a similar compartment: only the south side is exposed to outdoors. The roof is fitted with actuated alternate vents (44° maximum aperture, details are provided in S.1.1 and S.1.4).

The actual cultivated area is 944 m^2^ because of two East-West alleyways: the north one (concrete) is 3 m large while the south one (concrete covered with teardrop pattern steel sheet) is 1.2 m large. The compartment contains 15 rows of soil-less tomato crop (Clodano F1 Hybrid cultivar, Syngenta) cultivated on growing gutters at 1.1 m from the floor on a rock wool medium. The mature plant apex is maintained at 3.9 m from the ground. Below the cultivated area, the floor is covered by a white plastic sheeting.

As far as equipment are concerned, the compartment is fitted with:

- A thermal screen (Svensson Luxous 1547 D FR) and a shading one (Svensson Harmony 3315 O FR) that are unfolded over the crop at the truss (gutters) level.
- A thermal curtain at the south wall of the same material than the thermal screen.
- Three heating pipes networks: 1) the rail-51 loops close to the floor (one between each tomato row) 2) the Forcas loops in the crop (one per row) 3) the PE loops to avoid condensation close to the stems (one per row). Heat is provided by a natural gas boiler that supplies all the CTIFL facilities.
- Below 4 out of the 15 growing gutters extremities, an axial fan extracts air from the ambient and blows it into a perforated flexible (plastic) 0.8 m diameter ducts along the row.

The corridors are also equipped with their own thermal screens, thermal curtains, heating pipes (along the external walls) and vents (not shown on Fig 1).

Complementary information regarding the equipment is provided in S.1.

### 2.3. Data analysis and treatment

The present study uses a dataset recorded from the 3^rd^ of December 2014 (pre-planting) to the 1^st^ of November 2015 (end of the production season). Table 1 lists the measured values, their acquisition frequencies and how they are considered by the global model. Additional information and the dataset itself can be found in S2 File: in particular, this Supporting Information provides:

**Table 1:** List of recorded values.

Measurement/ source	Frequency	Notation	Type for the model
Outdoors			
Outdoor air temperature	5 min	*T*_*air,out*_	Input value
Outdoor air humidity	5 min	*RH*_*air,out*_ *, x*_*air,out*_	Input value
Outside global horizontal solar irradiance	5 min	*I*_*glob*_	Input value
Net longwave irradiance between the sky and the pyrgeometer	5 min	*I*_*lw,Sky-Pyr*_	Input value
Wind speed (8 m from the ground)	5 min	*U*_*wind,8m*_	Input value
Indoors			
Indoor air temperature (3.7 m high, “process” value)	5 min	*T*_*air,in*_	Evaluation value
Indoor air temperatures at 0.65 m, 1.5 m and 4.7 m from the ground	5 min	*T*_*air,in,L*_ *, T*_*air,in,M*_ *, T*_*air,in,H*_	Evaluation value
Indoor air humidity (3.7 m high, “process” value)	5 min	*RH*_*air,in*_ *, x*_*air,in*_	Evaluation value
Indoor air CO_2_ concentration (3.7 m high, “process” value)	5 min	*C*_*CO2,air,in*_	Input value
East corridor air temperature	5 min	–	Input value
North corridor air temperature	5 min	–	Input value
Agronomic data			
Leaf Area Index after each pruning	event	*LAI*	Evaluation value
Harvested marketable and unmarketable fruits quantity and weight	event	–	Evaluation value
Net irrigation volume	daily	–	Evaluation value
Crop length	weekly	*l*_*Crop*_	Input value
Equipment			
Injected heat through the rail 51 pipes	5 min	–	Input value
Injected heat through the Forcas pipes	5 min	–	Input value
Injected heat through the PE pipes	5 min	–	Input value
Injected CO_2_ mass	weekly	–	Evaluation value
Roof vents opening feedback	5 min	–	Input value
Thermal and shading screens position	5 min	–	Input value

- for each sub-dataset, all the experimental measurements pre-processing operations that have been done to manage issues such as data completion with other sources and punctual recording problems.
- The instrumentation details

#### 2.3.1. Weather station measurements.

**Fig 2:**
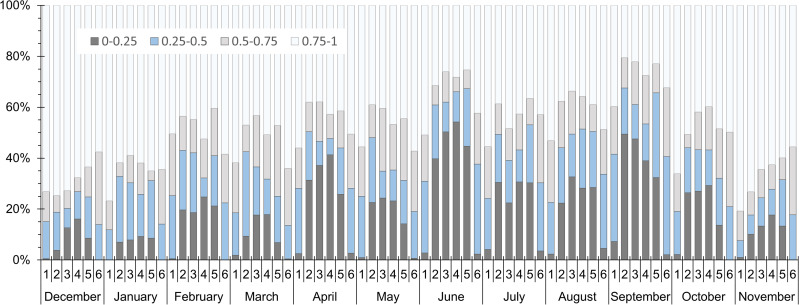
Distribution of the cloud ratio *Ce* from December 2014 to November 2015 close to Nantes (France). *Ce* was computed using data from the Copernicus Atmosphere Monitoring Service [38]. Each day is divided into 6 equal time frames from sunrise to sunset. For a given month and a given time frame, the ratio *Ce* falls into 4 categories: 0 → 0.25, 0.25 → 0.5, 0.5 → 0.75 and 0.75 → 1. The resulting percentage value (bar length on the y-axis) thus corresponds to the frequency at which the ratio is actually included in the range.

Nantes climate is categorized “Cfb” (temperate, no dry season, warm summer) in the Köppen-Geiger climate classification (see also S.1 Fig 2). Weather measurements were done on a mast close to the greenhouse at approximately 8 m high. A pyranometer (Hukseflux SR05) provided the solar global horizontal irradiance *I*_*glob*_: a good agreement with the open-access Copernicus Atmosphere Monitoring Service (CAMS) [38] data has been found for the same time period and location.

Since the CAMS data differentiates the direct and the diffuse parts depending on the local weather as detailed in [39,40], *I*_*glob*_ has been split into *I*_*dir*_ and *I*_*dif*_ using the ratio from the CAMS data at a 15 min time step. Defining the cloud ratio $Ce=I_{dif}/I_{glob}$ [41], Fig 2 exhibits a significantly varying nature of the irradiance depending on the month and the hour of the day. The diffuse part largely predominated during November, December and January, while the direct irradiance part was more pronounced in April and from June to September. As well, the diffuse nature often prevailed at the beginning and the end of the day (1^st^ and 6^th^ time frames).

A pyrgeometer (Hukseflux, IR02) measured the net longwave irradiance between the sky and the instrument *I*_*lw,Sky-Pyr*_. To compute the thermal radiation heat flux between the sky and any surfaces (greenhouse roof and south wall), the sky black body temperature *T*_*Sky*_ (K) was deduced from the pyrgeometer temperature *T*_*Pyr*_ (K), *I*_*lw,Sky-Pyr*_ and the Stefan-Boltzmann constant *σ* [17]:


$$T_{Sky}={\left[T_{Pyr}^{\text{4}}\;-\frac{I_{lw,Sky-Pyr}}{\sigma }\right]}^{\text{1}/\text{4}}$$ (1)

Following the manufacturer advice for such pyrgeometer fitted with an internal space heater (personal communication), in the absence of any precise instrument temperature measurement a 5 K offset has been applied, i.e., $T_{Pyr}=T_{air,out}+\text{5}$.

As well, the wind direction and speed are also included in the dataset.

#### 2.3.2. Indoor climate measurements.

**Fig 3:**
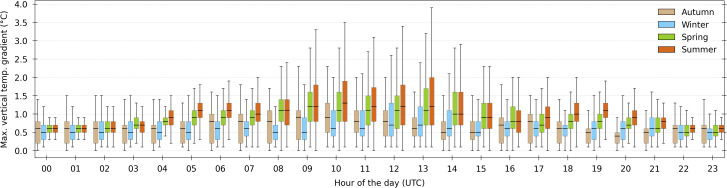
Maximum vertical temperature gradient distribution during the day and grouped per season. Distribution of the maximum vertical temperature gradient computed using the 4 measurements available in the dataset, depending on the hour of the day and the season (fliers are not shown). Measurement precision is 0.2 °C.

The compartment air temperature *T*_*air,in*_, humidity *RH*_*air,in*_ (or *x*_*air,in*_) and carbon dioxide concentration *C*_*CO2,air,in*_ were recorded above the 5^th^ row center (one single point at 3.7 m high, close to the mature plant apex): they are called “process” values since they were used by the climate supervision system for the control. In addition, three vertically aligned temperature sensors were installed close to the 11^th^ row center: *T*_*air,in,L*_, *T*_*air,in,M*_ and *T*_*air,in,H*_ at respectively 0.65 m, 1.5 m and 4.7 m from the floor. The measurements (*T*_*air,in*_, *T*_*air,in,L*_, *T*_*air,in,M*_ and *T*_*air,in,H*_) reveal a quite good vertical temperature homogeneity at the middle of the compartment: the maximum gradient is lower than 0.5 °C for 35% of the time, lower than 1 °C for 77.5% of the time, and lower than 2 °C for 97.1% of the time. As shown on Fig 3, on average the heterogeneity is more pronounced during summer between 09:00–15:00.

The highest gradients are restricted to specific time periods: they exceeded 4 °C only during 11 days. These levels of homogeneity can be explained by the presence of the air mixing ducts, which blow air at qualitatively perceptible velocity.

Regarding the corridors, only the air temperature was recorded at one single location (*T*_*air,Ncor*_ and *T*_*air,Ecor*_).

#### 2.3.3. Agronomic data.

The dataset includes the pruning dates and the target LAI value after each pruning up to the 246^th^ day after planting (on the 5^th^ of August 2015). Beyond that point, a weekly pruning was assumed at a target LAI of 4.5 m^2^ m^-2^. Topping was done on the 2^nd^ of September. Together with the harvest timestamps, the dataset also includes the quantity of harvested tomatoes (weight, number). The irrigation was monitored on a daily basis (total supplied volume, drained one, net irrigation). The crop length *l*_*Crop*_ was measured and recorded every week.

#### 2.3.4. Utilities and equipment.

For each of the three heating pipe networks, the supplied energy was recorded by energy counters with a 5 min time step, as well as the upstream water temperature. CO_2_ enrichment was applied the whole season using the boiler exhaust gases (when available) or from a liquid source: the total quantity of injected CO_2_ has been recorded weekly.

The windward and leeward roof vents opening feedbacks (analog signals) as well as the thermal and shading screens positions (analog signals) were recorded with a 5 min time step.

## 3. Models development

As it would not be possible to remind in a short way in this paper Vanthoor’s modelling work with the suitable level of detail, only the main developments are presented hereafter (the reader could refer to [17,19,23,24,42]). The resulting global model overview is introduced in subsection 3.1, while the other subsections emphasize the modifications on solar gains, air volume, airflows, heat and mass transfer and biological model. Although Modelica intends to provide a high abstraction level so that the model development can essentially consist in writing the laws of the physics, the actual implemented equations may slightly differ to manage specific issues (reverse flow, differentiability, problem initialization, etc.), compatibility with other models and simulation performance.

### 3.1. Global model overview

**Fig 4:**
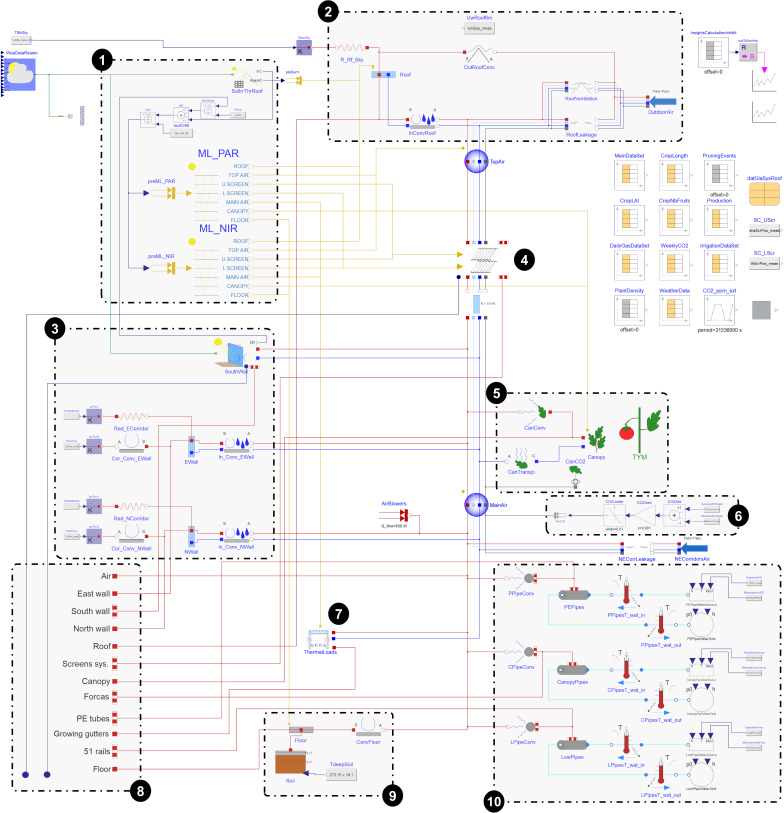
Top-level graphical view of the global model of the greenhouse compartment. Between the elements, connectors of different types are used. Red: heat transfer characterized by a temperature (K) and a heat flow (W) | Yellow: solar heat flux (W m^-2^) | Light blue: dry airflow characterized by a partial pressure (Pa) and a mass flow (kg s^-1^) | Dark blue: water vapor flow (same characterization) | Grey: CO_2_ flow (same characterization) | Green: weather bus, for the meteorological data and the sun position | Turquoise: hot water flows.

Fig 4 shows the top-level graphical representation of the experimental greenhouse model.

Each icon corresponds to a sub-model that can be itself made of other sub-models (object-oriented).

Several blocks can be highlighted:

1. Solar irradiance management through the roof and all the layers inside the greenhouse
2. Heat/mass transfer and balance implying the roof and its vents
3. Heat/mass transfer and balance through the south wall (and its curtain), the east and the north ones.
4. Horizontal screens system, further described in [43]
5. Biological sub-models: canopy transpiration, heat and CO_2_ transfer between the leaves and the air, energy balance and crop yield model
6. CO_2_ injection: an infinite CO_2_ source is assumed and the gas concentration is maintained in MainAir so that it constantly corresponds to the measurements
7. Thermal inertia sub-model, differentiating the greenhouse structure elements from the growing gutters and medium
8. Thermal radiation sub-model
9. Heat transfer with the floor and through the soil (multi-layer conduction)
10. Heating pipe networks including the convective heat transfer in air

### 3.2. Solar gains

The optical solar radiation wavelength band can be split into the UV (Ultra Violet), the PAR (Photosynthetically Active Radiation) and the NIR (Near Infra Red). The measured solar irradiance $I_{glob}$ includes the full solar spectrum, but it is usual in greenhouse climate and energy studies to explicitly manage only the PAR and NIR: an equal partition between PAR and NIR is often considered [44,45]. This approach has been kept, however, as it is reported in the literature since a long time [46] the ratio is in fact weather-dependent. Recent works [47] show that capturing this weather-dependency in a generic manner (i.e., whatever the location is and with open-access data) is still an on-going subject.

In addition to its wavelength band, the solar radiation is characterized by its directional attribute. The photons scattering is the main origin of the diffuse (omnidirectional) part, while the direct part hits the ground with a specific incidence angle which depends on the sun position in the sky [48].

Thus, the resulting transmittance of a surface such as a greenhouse roof exposed to the solar irradiance depends on the sun position in the sky, but also on the irradiance diffuse nature. In [42] original approach a constant greenhouse cover transmittance was considered: however, in his work perspectives he stressed the need to treat this aspect more precisely. As a matter of fact, assuming a constant value is not possible for the present modelling objectives. In order to represent properly short-term phenomena, the sun position in the sky and the meteorological conditions must be accounted for. A physic-based cover transmittance calculation methodology shall be used since:

- It may be difficult to justify the re-use of values determined in the literature for a specific case/location; in the present case, the south wall represents 17% of the greenhouse surface and can therefore transmit a significant amount of solar irradiance.
- A calibration process of the cover radiometric properties is not an option.
- Prospective greenhouse architectures may considerably differ from the existing ones in the literature.

Some detailed methods applied to multispan greenhouses exist in the literature, such as in [16] and [49]. The former has been chosen with modifications described hereafter.

#### 3.2.1. Direct irradiance through the roof.

In Bot’s [16] approach, the solar beam is modelled as a vector which originates from an unique point in the sky defined by the sun azimuth *α*_*s*_ and its zenith angle *γ*_*s*_. For Venlo roofs, the problem is symmetrical: denoting S_1_ the spans facing the sun and S_2_ the opposite spans, two incidence angles can be computed depending on the sun position, the greenhouse axis azimuth *α*_*g*_ and the roof slope *ψ*_*r*_. The model computes the number of spans that a beam is susceptible to pass through before reaching the greenhouse floor, and takes into account the associated multiple reflections on the bottom faces of S_2_ to the ground. As well, in some cases the reflection on upper faces of S_2_ can also contribute to increase the irradiance received on the greenhouse floor through the opposite S_1_ . These considerations lead to the explicit computation of the solar transmittance through the roof material (glass) denoted *τ*_*g*_. However, the resulting solar direct roof transmittance *τ*_*r,dir*_ shall also consider the interception by the opaque elements of the roof (ridges, gutters and glazing bars). Bot [16] demonstrated the formulas to compute the solar transmittances of the {ridges+gutters} system *τ*_*rg*_ and the glazing bars *τ*_*b*_, function of the greenhouse structure dimensions, the element sizes and the sun position. Finally, the resulting roof direct transmittance is:


$$\tau_{r,dir}\left(\alpha_{s},\gamma_{s}\right)=\tau_{g}\left(\alpha_{s},\gamma_{s}\right)\cdot \tau_{rg}\left(\alpha_{s},\gamma_{s}\right)\cdot \tau_{b}(\alpha_{s},\gamma_{s})$$ (2)

The present implementation of this model follows Bot’s methodology extensively described in [16] (from p. 76–109), but with a few differences. For example, instead of implementing the Fresnel equations for the transmittance and reflectance of a single span glass, instances of the detailed “Window” sub-model [33] were used: the latter enables to compute the optical properties of more or less complex glazing (e.g., multi-layer ones with asymmetrical properties). While it initially deals with symmetrical Venlo-type roofs, Bot’s methodology can be applied to other multispan roof shapes.

The sub-model outputs have been checked through the reproduction of Bot’s [16] results for North-South and East-West oriented greenhouses (52° latitude North, with common structure properties) at various days of the year (refer to Fig 3 in the S.1).

#### 3.2.2. Diffuse irradiance through the roof.

Knowing the direct solar transmittance of the roof from any direction in the sky vault, the diffuse solar irradiance one *τ*_*r,dif*_ can be computed as the integration over the whole sky of a radiance distribution law [16]:


$$\tau_{r,dif}=\frac{\int_{\alpha =\text{0}}^{\text{2}\pi }\int_{\gamma =\text{0}}^{\pi /\text{2}}\tau_{r,dir}(\alpha ,\gamma )\cdot L(\alpha ,\gamma )\cdot \text{sin}\gamma \cdot \text{cos}\gamma d\gamma d\alpha }{\int_{\alpha =\text{0}}^{\text{2}\pi }\int_{\gamma =\text{0}}^{\pi /\text{2}}L(\alpha ,\gamma )\cdot \text{sin}\gamma \cdot \text{cos}\gamma d\gamma d\alpha }$$ (3)

With *α* the azimuth (rad) and *γ* the zenith angle (rad) of the infinitesimal element of the sky vault and *L(α,γ)* the radiance of the element. Several distribution models have been considered and discussed by Bot [16] for limited sky conditions (overcast and clear skies). Considering 1) more recent scientific advances 2) the fact that weather data are now widely and freely available in many places in the world 3) the need for year-round models to reflect seasonal weather variability, it appeared necessary to the authors to update this approach. In particular, the All-Sky Model-R from [41] provides a continuous relative radiance distribution computation method based on meteorological indexes calculated from *γ*_*s*_, *I*_*glob*_, *I*_*dif*_, *α* and *γ*.

The model development followed the aforesaid methodology, although with two noticeable remarks. Firstly, the implementation in Modelica requires an explicit discretization of the sky vault to compute the integrals in Eq. (3): the integration step is a parameter of the sub-model and for the present work a 5° value for *α* and *γ* was considered. Secondly, since *τ*_*r,dif*_ only depends on external variables (weather) and defined parameters, it can thus be pre-computed to reduce the simulation time for the global experimental greenhouse model. Similarly, evaluation of cover materials with controlled radiometric properties can be achieved through the interpolation between several pre-computed control cases.

**Fig 5:**
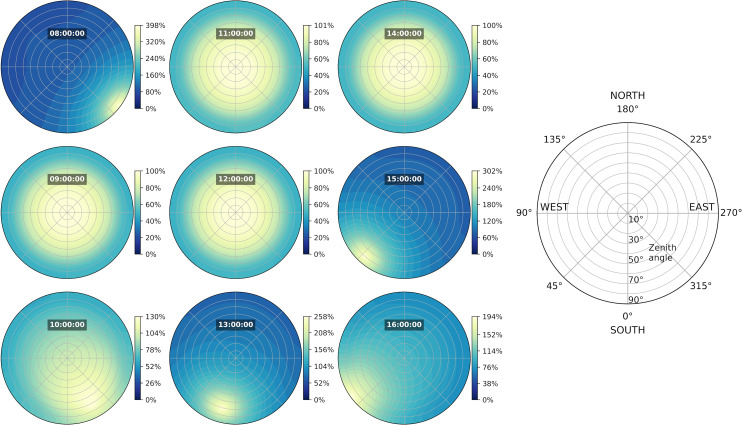
Relative sky radiance distributions computed the 5^th^ of December 2014 close to Nantes (France). Polar views of the relative sky radiance distributions (All-Sky Model R from Igawa [41]) calculated between sunrise and sunset the 5^th^ of December 2014, hourly sampled for this example. For instance, the maximum value at 13:00 (258%) means that the sky element with the highest radiance had a value 2.58 times the zenith one.


Fig 5 illustrates the distribution of the relative sky radiance *L’(α,γ)* on the 5^th^ of December 2014, computed by the model and defined relatively to the zenith sky element radiance *L*_*z*_:

$$L^{\prime }(\alpha ,\gamma )=\frac{L(\alpha ,\gamma )}{L_{z}}$$ (4)

It exhibits varying sky conditions between sunrise and sunset: a standard overcast sky situation happened at 09:00, 11:00, 12:00 and 14:00, while the relative radiance was more or less directional the rest of the day.

#### 3.2.3. Solar irradiance transmission in the greenhouse.

Once the solar irradiance has entered the greenhouse, it can be absorbed, reflected or transmitted by several layers: the inside roof surface, the screens, the canopy and the floor. The existing implementation of the solar irradiance transmission inside the greenhouse was modified to correspond to the present context:

- The experimental greenhouse has two horizontal screens instead of one.
- Because of the alleyways, the cultivated area is smaller than the greenhouse floor area (the surface ratio *r*_*cul*_ being equal to 0.91)
- To use the model for prospective purposes, it appeared interesting to build a model that could evaluate optically anisotropic layers, layers with a wavelength-dependent behavior (such as controlled colored screens [50]) and materials with switchable radiometric properties [44,45].

In the present modelling approach, the transmittance, the reflectance and the absorptance through the horizontal layers inside the greenhouse are computed similarly to the transmission through a multiple glazing window according to [51] (Eq. A.4.78.a-b-c, A.4.82, A.4.81.a-b, A.4.83.b) as detailed in [43]. Applied to the experimental greenhouse, the multilayer model is made of five elements: the roof, the shading screen, the thermal screen, the canopy and the floor. For this study case, only two instances are used (one for the PAR, one for the NIR).

For the canopy layer, the equations based on extinction coefficients *K*_*PAR*_ and *K*_*NIR*_ [42] are adapted to take into account the *r*_*cul*_ ratio. For instance, the canopy layer PAR transmittance *τ*_*Can,PAR*_ is computed:


$$\tau_{Can,PAR}=r_{cul}\cdot \text{exp}(-K_{PAR}\cdot LAI)+(\text{1}-r_{cul})$$ (5)

As far as the roof layer is concerned, the downwards transmittance is computed in the model described in subsections 3.2.1 and 3.2.2, but the upwards one is fixed at the value of *τ*_*r,dif*_ computed considering a uniform radiance, i.e., assuming that the upwards reflections in the greenhouse are diffuse and homogeneous. For each layer including the roof one, the absorbed irradiance calculated with [43] is added to their corresponding energy balance equations.

#### 3.2.4. South wall.

The solar gains through the south wall are computed using the Window sub-model from the “Buildings” library [33] by analogy with a virtual window oriented with the suitable azimuth, a coefficient to account for the frames and the dirtiness as well as a fixed ground albedo for the diffuse part. The absorbed irradiances for the glass itself and for the indoor curtain are added to the respective energy balance equations. In the present model, the solar irradiance entering the greenhouse through the south wall is added to the one entering through the roof, so that it is managed in the same way by the multilayers model PAR and NIR instances.

The net short-wave flow through the three other walls is considered null.

### 3.3. Air volume

The widespread approach consisting in dividing a greenhouse into two zones *MainAir* (below the screens) and *TopAir* (above) was kept: it has indeed been shown, at least for the temperature, that the perfectly stirred tank approach is most of the time acceptable for the experimental greenhouse (refer to subsection 2.3.2). For other case studies where no a priori knowledge is available, a finer approach would probably be more suitable (it will be presented as a perspective at the end of this paper).

The original energy balance equation is also commonly applied in the literature [20], using a formula like $\varrho_{air}\cdot V_{air}\cdot Cp_{air}\cdot {\dot{T}}_{air}=\sum heat\;fluxes$ with *ϱ*_*air*_ the air density, *V*_*air*_ the volume of air, *Cp*_*air*_ its specific heat capacity and *T*_*air*_ its temperature. However, *ϱ*_*air*_ is a function of the air temperature, humidity and absolute pressure. The underlying assumption regarding the pressure (isobaric conditions) leads to mass balances issues if applied. Thus, a new Air Volume model needed to be developed, implementing energy (enthalpy) and mass conservation equations on dry air, water vapor and CO_2_. The greenhouse volumes (*MainAir* and *TopAir*) being constant, isochoric conditions are considered. It is however clear that the indoor volumes mean pressures always remain close to the outdoor one at the same average height. Nevertheless, the resulting isobaric condition is the consequence of air mass flows through the cover governed by pressure differences, which need to be explicitly computed for a correct mass balance at the global compartment scale. Besides, this is a mandatory step towards more details zonal models, as well as towards the assessment of Heating, Ventilation and Air-Conditioning (HVAC) systems integration. The latter are indeed susceptible to influence the air exchanges (so the losses) with outdoor through their effects on the neutral level, air volumes pressurization or depressurization. Consequently, the energy balance is applied to the internal energy *U* (J), i.e., the derivative of *U* equals the sum of the heat flows from/to the volume. *U* is linked to the moist air specific enthalpy *h* (J kg_dair_^-1^) through the fact that H=U+p·V (with *H* the enthalpy (J), *p* the absolute pressure (Pa) and *V* the volume (m^3^)) and $H=M_{dair}\cdot h$ (with *M*_*dair*_ the mass of dry air in the volume, in kg). *h* is itself a function of the temperature *T* (K), the humidity ratio *x*_*w*_ (kg_w_ kg_dair_^-1^) and *p*. *p* is the sum of the partial pressures of the dry air and the water vapour (Dalton’s law): considering the maximum CO_2_ concentration usually met in greenhouses, its effects on *p* are ignored. The partial pressure of a given gas is linked to its mass in the volume by the perfect gas law, and the mass flow balance for each gas is computed using mass conservation of species on the volume. Well-known moist air formulas are used to link *x*_*w*_ to the water vapour pressure *p*_*w*_, to link *p*_*w*_ to the relative humidity, etc. [52]. The model also explicitly computes the moist air density so that it can be used as a variable for the airflow calculations sub-models.

### 3.4. Airflows

#### 3.4.1. Natural ventilation.

The natural ventilation through vents is abundantly studied in the literature dedicated to protected cultivation, both experimentally and numerically. Although the underlying phenomena (buoyancy and wind effects) are well defined, their resulting performance in terms of air exchange is tributary to the vents configuration (location, shape, insect screens, etc.) and to airflow obstacles inside the greenhouse (crop, horizontal screens, etc.).

Limited to the present evaluation case (Venlo-type greenhouse with alternated roof vents), a model was already implemented by [23] but has been slightly modified.

The buoyancy effect is driven by the air density difference through the vent, which was converted into a temperature difference using the Boussinesq approximation, neglecting the effects of the humidity on the air density as it is usually done in the literature [16,53,54]. The buoyancy effect through a vent is minor when the wind speed is greater than 2 m.s^-1^ [16,53,55]: consequently, neglecting the effect of humidity could actually have an impact only at low wind speed. However, air densities as functions of the temperature, humidity and pressure can be used in the present model at no cost since they are made available in the Air Volume model (refer to subsection 3.3). The buoyancy airflow through one leeward vent *F*_*buoy,L*_ (m^3^ s^-1^) is thus computed [56]:


$$F_{buoy,L}\left(\delta_{L}\right)=\frac{C_{f}\cdot L_{O}\cdot {\left[H_{O}\cdot (\text{sin}\psi_{r}-\text{sin}\left(\psi_{r}-\delta_{L}\right))\right]}^{\text{3}/\text{2}}}{\text{3}}\cdot \sqrt{g\cdot \frac{\left|\varrho_{TopAir}-\varrho_{air,out}\right|}{\varrho_{ref}}}$$ (6)

With *C*_*f*_ the discharge coefficient, *L*_*o*_ the length of the vent, *H*_*o*_ its height, *δ*_*L*_ the opening angle, *g* the acceleration of gravity, *ϱ*_*TopAir*_ the *TopAir* air density, *ϱ*_*air,out*_ the outdoor air one and *ϱ*_*ref*_ the density being here considered as the average between *ϱ*_*TopAir*_ and *ϱ*_*air,out*_ for simplification. Eq. (6) also applies for a windward vent *F*_*buoy,W*_.

As far the wind effect is concerned, since a reasonably similar compartment has already been studied in the literature (Fig 4.1 and 4.2 of [56]), the corresponding ventilation functions formulas are used. Denoting *G*_*L*_*(δ*_*L*_*)* the ventilation function (-) of the leeward vents and *G*_*W*_*(δ*_*W*_*)* (-) the windward vents one:


$$G_{L}\left(\delta_{L}\right)={\text{2}.\text{4}\text{6}\cdot \text{1}\text{0}}^{-\text{2}}\cdot (\text{1}-\text{exp}(-\delta_{L}/\text{1}\text{4}.\text{5}))$$ (7)

$$G_{W}\left(\delta_{W}\right)=-{\text{1}.\text{8}\text{9}\cdot \text{1}\text{0}}^{-\text{5}}\cdot \delta_{W}^{\text{2}}+{\text{2}.\text{2}\text{3}\cdot \text{1}\text{0}}^{-\text{3}}\cdot \delta_{W}$$ (8)

With *δ*_*L*_ and *δ*_*W*_ in °. Although Eqs (7) and (8) are experimentally determined for *δ ≤ 29°* and a wind speed between 3 and 6 m s^-1^, it is assumed that they are valid for *δ* up to 44° and the entire dataset wind range.

The wind-induced airflow through a leeward vent *F*_*wind,L*_ (m^3^ s^-1^) is computed [56]:


$$F_{wind,\;L}\left(\delta_{L}\right)=L_{O}\cdot H_{O}\cdot G_{L}\left(\delta_{L}\right)\cdot U_{wind,r}$$ (9)

With *U*_*wind,r*_ the wind speed at the roof height (m s^-1^). Eq. (9) also apply for the windward vent wind-induced airflow *F*_*wind,W*_ (m^3^ s^-1^). Finally, the total airflow through the roof vents per greenhouse floor area (m^3^ s^-1^ m^-2^) is computed considering that the leeward and windward vents surfaces could be different:


$$F_{vents}\left(\delta_{L},\delta_{W}\right)=\sqrt{{\left[N_{L}\cdot F_{buoy,L}\left(\delta_{L}\right)+N_{W}\cdot F_{buoy,W}\left(\delta_{W}\right)\right]}^{\text{2}}+{\left[N_{L}\cdot F_{wind,L}\left(\delta_{L}\right)+N_{W}\cdot F_{wind,W}\left(\delta_{W}\right)\right]}^{\text{2}}}$$ (10)

With *N*_*L*_ and *N*_*W*_ the number of leeward and windward openings per m_floor_^-2^.

The existing approach was also modified considered that whatever the horizontal screens positions are, the airflow mixing occurs beforehand with the *TopAir* zone. Ventilation functions such as those of Eqs (7) and (8) being specific to a greenhouse configuration, the model was also modified so that functions experimentally determined by other authors in the literature could be easily added and selected in a dropdown menu interface.

#### 3.4.2. Air leakages.

As a general rule, air infiltration in a building can depend on the indoor/outdoor air temperatures difference, the building height and its shape, its airtightness and its exposure to the wind [57]: applied to plastic arch-roof greenhouses [58], has for instance observed the strong effect of the wind direction and the cover tightness. Putting aside any poor quality greenhouse covering, according to [59] the leakages are mainly attributable to the imperfect window closure. In the pre-existing implementation, infiltrations were addressed using coefficients reflecting the amount of air exchange (in terms of volume per hour) with a wind-speed dependent linear relationship. One may find difficult, without any calibration process, to find suitable coefficients values for a specific greenhouse. More physic-based methodologies are used in building [52], relying on differential pressure calculations across walls and the estimate of probably more accessible (or comparable) parameters such as equivalent leakage areas. In models, resulting indoor air pressures magnitude can also be a clue to evaluate the consistency of those parameters.

Applied to the present experimental greenhouse, the main issue was to model the fact that the infiltrations could come from either outside or the corridors. Since no pressure measurements were available in the corridor, an intermediary approach was applied and the leakages were considered the result of:

- The air exchange through the vents cracks. A virtual height *h*_*vent,leak*_ (mm) is defined as a parameter in the natural ventilation model (refer to subsection 3.4.1) so that when the vents are closed they actually remain slightly opened at this value. This approach is an attempt 1) to include the dynamics of the natural ventilation (buoyancy and wind effects) 2) to reflect the fact that the air leakage occurs in first place with the air close to the vents 3) to include the dependency on the total window surface in the greenhouse 4) to be a step towards more space-discretized approaches. It shall, however, be pointed out that the phenomena at such very low opening angles are probably different from those observed when the vents are actually opened.
- The pressure balance between zones, i.e., for the experimental greenhouse model 1) the *TopAir* volume and outdoor air 2) the *MainAir* volume and the corridors. Air exchanges with the west compartment are supposed to be neutral in terms of energy and mass balances (same cultivation practices). The south wall, through which there is no window or door, is assumed airtight in comparison with the north and east ones.

The airflows resulting of a pressure difference through a wall or through the roof are modelled using a power law:


$$F_{crack}=sign(\Delta P)\cdot \eta \cdot |\Delta P{|}^{n}$$ (11)

With *n* = 0.65 (-) [52] and *η* a flow coefficient (m^3^ s^-1^ Pa^-n^). The determination of the values for *η* is described in S.1. Interestingly, a physically-based *h*_*vent,leak*_ value of 6 mm [60] provides consistent behavior both regarding the resulting indoor air pressure magnitude and the leakages found experimentally by [61] for a similar greenhouse compartment. One may argue that the latter could have been directly used: it is reminded here that the purpose is to investigate the possibility of a more generic approach, compatible with the assessment of HVAC systems integration that may affect the leakages through their effect on the indoor pressure.

#### 3.4.3. Airflow between zones separated by horizontal screens.

Because of the presence of two horizontal screens instead of one in the evaluated greenhouse study case, the approach from [17] and [42] had to be slightly modified. Four continuously managed folding cases are possible instead of two: 1) No screen unfolded, 2) Thermal screen (lower screen) unfolded, 3) Shading screen (upper one) unfolded 4) Both screens unfolded. Each situation corresponds to the superposition of these 4 states characterized by the association of the unfolding states [0:1] of the lower and upper screens. The equivalent screen permeability is computed using an analogy with electrical conductances in parallel and series. A leakage is also introduced to account for the imperfect sealing along the screens [62], and air density as function of the temperature, humidity and pressure gradient across the horizontal screens system is used in the equations.

#### 3.4.4. Conversion to enthalpy and mass flows.

All these airflow sub-models compute volumetric airflows, which need to be converted into enthalpy and mass flows. This is done using a generic abstract model from which all of them inherit, and being settable to work for an unilateral (e.g., airflow through a crack) or bilateral (e.g., through the screens or the vents) case. Considering a known volume airflow rate *F* (m^3^ s^-1^) between two air volumes *1* and *2*, the perfect gas law applied to all the gas species *i* (dry air, water vapour, CO_2_) gives the corresponding net mass flow rate from 1 to 2 ${\dot{m}}_{i,\text{1}\to \text{2}}$ (kg s^-1^) as the difference between the mass leaving *1* and the one leaving *2*:


$${\dot{m}}_{i,\text{1}\to \text{2}}={\dot{m}}_{i,\text{1}}-{\dot{m}}_{i,\text{2}}=\;F\cdot \frac{M_{i}}{R}\cdot \frac{k_{\text{1}}\cdot p_{i,\text{1}}}{T_{\text{1}}}-F\cdot \frac{M_{i}}{R}\cdot \frac{k_{\text{2}}\cdot p_{i,\text{2}}}{T_{\text{2}}}$$ (12)

With R the gas constant (J kg^-1^ mol^-1^), ${\dot{m}}_{i,\text{1}\to \text{2}}$ positive if the net flux is from *1* to *2*, and negative otherwise. *k*_*1*_ and *k*_*2*_ (-) are coefficients:

- For an airflow through the vents, *k*_*1*_ *= k*_*2*_ *= 1*.
- For a unidirectional flow such as a leakage in a crack due to a pressure difference, if *p*_*1*_ *> p*_*2*_ then *k*_*1*_ *= 1*, else *k*_*1*_ *= 0*. In both cases, *k*_*2*_ *= 1 – k*_*1*_.

The corresponding net sensible heat flux from *1* to *2* (W) is computed:


$${\dot{Q}}_{\text{1}\to \text{2}}=\sum {\dot{Q}}_{i,\text{1}\to \text{2}}=\sum \left[{\dot{m}}_{i,\text{1}}\cdot Cp_{i}\cdot T_{\text{1}}-{\dot{m}}_{i,\text{2}}\cdot Cp_{i}\cdot T_{\text{2}}\right]$$ (13)

With the temperature *T*_*1*_ and *T*_*2*_ in °C.

### 3.5. Heat and mass transfer

#### 3.5.1. Convection.

Compared to the existing implementation from [23], some evolutions have been provided:

- A generic abstract model has been implemented to compute the heat transfer between any indoor surface and the air.
- While it is common to use correlations determined by laboratory experiments for small free-edge plates, their application to real scale in buildings [63] or in greenhouses [54,64] may show some noticeable differences. The correlations used for this work are detailed in S.1.5. It is acknowledged that they are susceptible to be influenced by factors such as vents opening [65] and more generally to be affected by anything that can significantly impact indoor air movements during a whole production year. These dependencies have not been taken into account in the present work, but this is a perspective.

The widespread implementation of the Lewis analogy used by [17] to compute the water mass transfer (condensation and evaporation) is kept.

Where the horizontal screens system is used, the global heat transfer between air zones is continuously computed following the 4-cases superposition principle described in section 3.4.3. In the section where both screens are unfolded, the heat transfer through the separating air layer is computed according to [66]. The water mass transfer is limited by the condensed water availability on the lower screen (same principle as applied in the original single-screen implementation).

For walls fitted with a curtain, for the section where the latter is unfolded close to the glass, the analogy with a convective heat transfer through a vertical air cavity is applied: the “GasConvection” sub-model of the “Buildings” library [33] was modified to take into account the water vapor mass transfer through the curtain.

Outside the greenhouse:

- On the roof, the original implementation of [23] using correlation from [16] which is applicable to Venlo-type shape is considered for the present evaluation study case.
- The convective heat transfer between outdoor air and a wall looking outwards is computed using the existing implementation of the TARP method [67] in the “Buildings” library.

For separation with another indoor air volume (such as the corridors walls in the evaluation study case), the air is supposed to be still and the correlation between an air volume and a vertical surface is used.

#### 3.5.2. Thermal radiation.

The net radiative heat transfer ${\dot{Q}}_{S\text{1}\to S\text{2}}$ (W) between two surfaces S_1_ and S_2_ separated by *N* media is modelled:


$${\dot{Q}}_{S\text{1}\to S\text{2}}=\frac{\varepsilon_{\text{1}}\cdot \varepsilon_{\text{2}}\cdot {FF}_{\text{12}}\cdot {\text{A}}_{\text{1}}\cdot \tau_{x\text{1}}\cdot \tau_{x\ldots }\cdot \tau_{x\text{N}}\cdot \sigma \cdot (T_{\text{1}}^{\text{4}}-T_{\text{2}}^{\text{4}})\;}{\text{1}-\rho_{\text{1}}\cdot \rho_{\text{2}}\cdot F_{\text{21}}\cdot F_{\text{12}}\cdot \tau_{x\text{1}}^{\text{2}}\cdot \tau_{x\ldots }^{\text{2}}\cdot \tau_{x\text{N}}^{\text{2}}}$$ (14)

With *ε*_*1*_ and *ε*_*2*_ the emissivity of S_1_ and S_2_, *FF*_*12*_ the view factor from S_1_ to S_2_ (reciprocally for *FF*_*21*_), *A*_*1*_ the area of S_1_ , *τ*_*x,i*_ the transmissivity of the *i*^*th*^ medium, *σ* the Stefan-Boltzmann constant, *T*_*1*_ and *T*_*2*_ the surface temperatures of S_1_ and S_2_ (K), *ρ*_*1*_ and *ρ*_*2*_ the surfaces reflectivity. Applied to the greenhouse study case, Table 2 lists all the radiative heat transfer cases taken into account. Obviously, some of them could have been neglected without impairing the whole energy balance: for instance, it is expected that the north and east walls temperatures remain always close to each other. However, they are considered to evaluate, from a numerical point of view (simulation time, initialization problem solving and solver choice), the feasibility of a more complex thermal radiation transfer network. As well, the denominator in Eq. (14) could be ignored most of the time considering the high emissivity values (so low reflectivity ones) of surfaces inside a greenhouse (glass, leaves, etc.).

**Table 2:** Possible radiative heat transfer cases: a « X » means that the case is actually computed in the model.

	Floor	Canopy	Hor. Screens^a^	Roof	South wall^b^	North wall	East wall	51 rails pipes	Forcas pipes	PE pipes	Growing gutters	Ducts^c^	West wall^d^
Floor		X	X	X	X	X	X	X	X	X	X	X	X
Canopy			X	X	X	X	X	X	X	X	X	X	X
Hor. Screens^a^				X	X	X	X	X	X	X	X	X	X
Roof					X	X	X	X	X	X	X	X	X
South wall^b^						X	X	X			X	X	X
North wall							X	X			X	X	X
East wall								X	X	X	X	X	X
51 rails pipes											X	X	X
Forcas pipes											X	X	X
PE pipes											X	X	X
Growing gutters												X	X
Ducts^c^													

^a^One sub-case per horizontal screen

^b^One case for the wall itself, another one for the curtain

^c^The flexible mixing ducts thermal inertia is neglected and their temperature is assumed to equal the MainAir one: consequently, the radiative heat transfer cases implying the ducts are attributed to the latter.

^d^Since the cultivation practices in the other compartment on the west side are the same, the wall temperature is assumed to equals the MainAir one and the radiative transfer cases implying this wall are directly attributed to the latter.

While most parameters in Eq. (14) can be set based on manufacturer data, specific measurements and the literature, view factors determination is more complex. Indeed, they depend on the greenhouse geometry, the control of equipment (screens, curtain), the presence of obstacles and surfaces which evolve during the production season (canopy). For that purpose, a generic calculation methodology has been detailed in [68,69], mixing analytical and numerical approaches. Based on the PyViewFactor module [70] and PyVista framework [71], the numerical procedure follows several steps:

1) Geometry design, where all the suitable greenhouse elements are built and located on a scene using a parametric approach.
2) Elements discretization in small cells.
3) Batch view factor computation between the cells of the source and receptor elements using the contour method [72] and raytracing for obstacle detection.
4) Post-treatment of elementary view factors to retrieve the resulting values from/to the chosen source and receptor elements.
5) Batch computation of steps 3) and 4) for different crop row heights and widths.
6) Regression formula calculation, linking the view factor to the crop rows height/width.
7) Batch computation of steps 3), 4), 5) and 6) for all the cases in Table 2.
8) Regression formulas global normalization so that the sum of all view factors related with a specific element always equals 1.

Finally, in the Modelica model, each instance implementing Eq. (14) is fed with the corresponding regression formulas.

Thermal radiation between the cover and the sky is computed according to the sky black body temperature obtained with Eq. (1). The complementary part for a wall that looks outwards is computed considering the ground as a black body at the outdoor air temperature. Similarly, walls separating a compartment to other air volumes (such as corridors) are supposed to face black-bodies at the average temperature between the corridor air and the outdoor air one.

### 3.6. Biological model modifications

The Tomato Yield Model (TYM) in the “Greenhouses” library is an implementation of the model developed by [25]. The main changes regarding the latter concerns implementation performance and the leaves pruning management: while in the original model it is managed continuously considering a maximum bound for the LAI, the pruning is now discrete. A time-table parameter is added to the model: for each pruning event timestamp, a target (or final) LAI is set. A detailed model diagram is given in the Supplementary Information S3 File. As for the crop transpiration model, it is strictly the same as in [73].

### 3.7. Models parametrization for the case of study

#### 3.7.1. Tomato yield sub-model.

Although the plant growth model from [25] was used, its parameters values were adapted to depict the behavior of the Clodano F1 Hybrid cultivar (Syngenta) during the study case experiment. Photosynthesis response to environmental factors has been kept as it was, but plant growth parameters had to be modified: details are provided in S.1.4.13.

Parameters values determination was performed by forcing the experimental climate conditions and crop management (pruning) and adjusting the growth parameters values according [74] and parameter sweeping. Since Vanthoor’s model [25] outputs a Dry Matter content (DM), a conversion to fresh weight had to be applied. As a general rule, the conversion value depends on the tomato type and varieties, as well as on the time of the year (season) [74,75]. DM content in the harvested fruits is also tributary to the producer choice regarding the maturation at which the fruits are actually harvested. In accordance with the CTIFL, a constant Dry Matter content of 5.4% was set.

#### 3.7.2. Numerical considerations.

The parameter values considered for the present case of study are detailed in the S.1.4. From a numerical point of view, the global model includes 2890 time-dependent variables. Using Dymola (tolerance of $\text{1}\cdot {\text{1}\text{0}}^{-\text{5}}$ and 5 min output sampling), it takes approximately 37 min (with DASSL) and 20 min (with CVODE) to run the simulation over 334 days on a laptop PC with an i7-1165G7 CPU.

## 4. Results and discussion

Several kinds of experimental greenhouse model outputs are presented in this section. Firstly, the variations of the roof direct and diffuse transmittances are quantified. These variables imply no coupling, as they only depend on the roof characteristics, the location and the weather. On the contrary, indoor temperature and humidity result from the interdependencies of multiple phenomena: climatic outputs are thus shown, discussed and related metrics are compared to the literature. Then, the biological sub-models outputs (yield, fruits quantity and LAI) are illustrated since they are of prime importance for the present modelling objectives. Water and CO_2_ mass balances are analyzed and model limitations are discussed. Outputs comparisons with global model variants using less detailed approaches are also shown.

### 4.1. Solar models outputs

**Fig 6:**
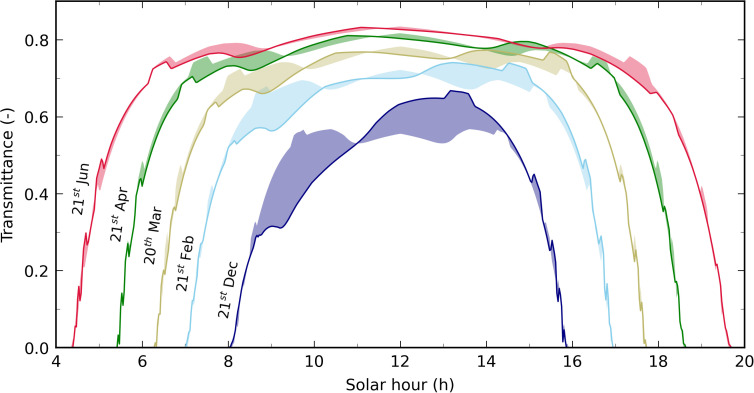
Resulting direct transmittance through the experimental greenhouse roof *τ*_*r,dir*_ at specific days of the year. For each curve, the associated colored area corresponds to the transmittance difference of a very same roof perfectly N-S oriented.

The resulting direct transmittance through the roof *τ*_*r,dir*_ is plotted for several specific days of the year on Fig 6. Its shape obviously depends on the glass and roof structure characteristics: in particular, the noticeable asymmetry from noon is caused by the fact that the greenhouse is not exactly N-S oriented. The difference is more pronounced during winter, when the sun altitude remains low and when its azimuth leads to higher incidence angles on the roof east-facing panes (morning) and lower ones on the west-facing panes (afternoon). Compared to a N-S orientation, the roof transmittance is most of the time reduced during the first part of the day, while it is increased during the afternoon: the absolute difference can reach 0.07 (-) (the 20^th^ of March 2015), 0.11 (-) (the 21^st^ of February 2015) and 0.19 (-) (the 21^st^ of December 2014). The summer solstice curve (21^st^ of June 2015) shows that the direct transmittance is also slightly reduced after 4 p.m. It is also the case for the curve of the 20^th^ of March 2015 and 21^st^ of April 2015 at the end of the afternoon: the west-facing panes being slightly oriented to the south, at an equivalent solar altitude the incidence angle minimum is thus reached before.

However, the actual effect of the greenhouse orientation on the total roof transmittance depends on the directional characteristic of the irradiance reaching the roof. For a uniform sky (where the sky radiance is constant) or a standard overcast sky (where the radiance only depends on the sky element altitude angle (as shown at certain times of the day on Fig 5)), the greenhouse orientation does not matter.

**Fig 7:**
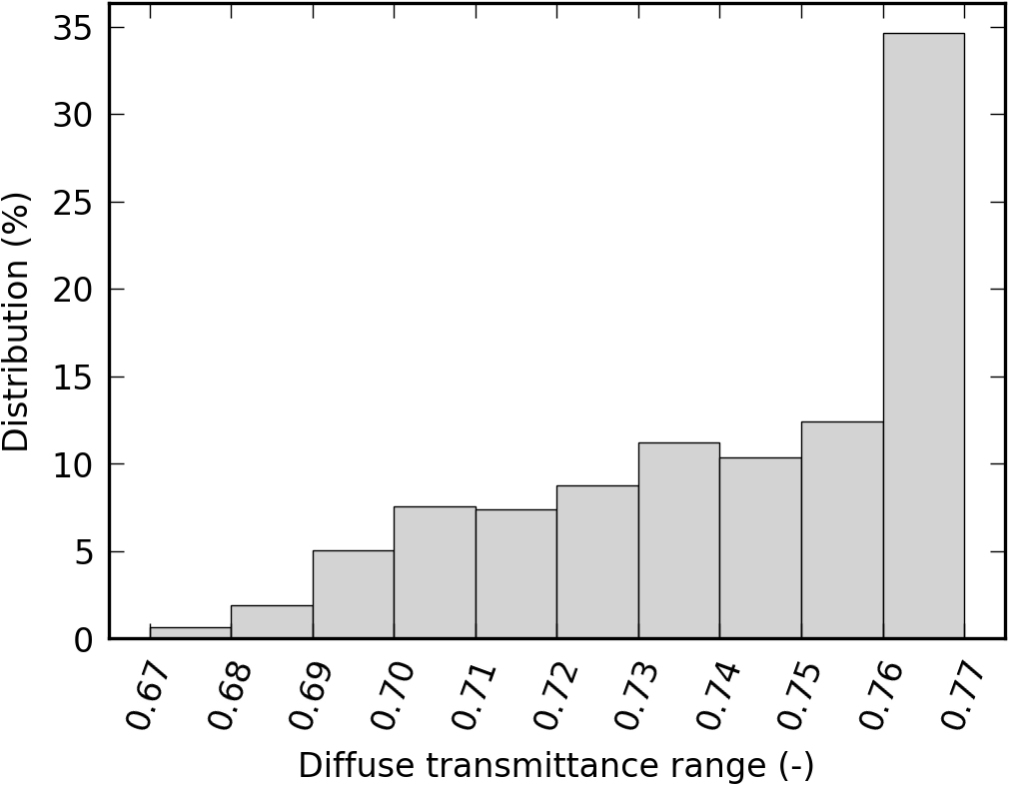
Distribution of *τ*_*r,dif*_ over the whole production season for the experimental greenhouse. Note: stack heights slightly depend (from an order of magnitude typically less than 2% in absolute value) on arbitrary choices concerning numeric effects handling (in the model) and post-treatments (such as rounding).

Over the whole simulation period, *τ*_*r,dif*_ remained limited between 0.67 and 0.77 (-), and was often higher or equal to 0.76 (Fig 7). These variations occurred at the day scale (as the weather changes), but no particular trend is observed at the month or season time scale. *τ*_*r,dif*_ being dependent on *τ*_*r,dir*_ (Eq. (3)), the greenhouse characteristics necessarily have an impact: for a standard overcast sky, *τ*_*r,dif*_ is close to 0.77 (-) for the present roof, while it is equal to 0.72 (-) in [16] one and 0.79 (-) in [17]. The direct and diffuse transmittances through the south wall being computed by analogy with a large vertical window, the resulting values are more trivial and are not shown in this paper.

### 4.2. Greenhouse indoor climate

**Fig 8:**
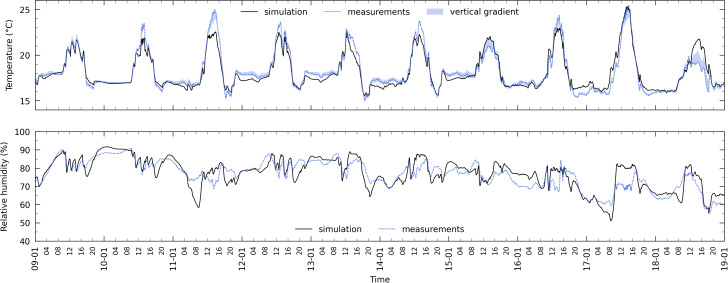
Temperature and relative humidity in the MainAir zone during 10 winter days. Main air zone temperature (top) and relative humidity (bottom) during 10 winter days from the 09^th^ to the 19^th^ of January 2015 (black solid line: simulation | blue dotted line: “process” measurements *T*_*air,in*_ and *RH*_*air,in*_ (at the plant apex)). The blue area on the upper graph delimits the temperature vertical gradient as the difference between the minimum and maximum of *T*_*air,in,L*_, *T*_*air,in,M*_, *T*_*air,in*_ and *T*_*air,in,H*_.

**Fig 9:**
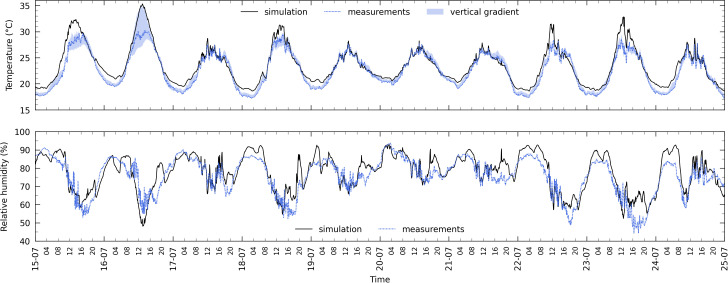
Temperature and relative humidity in the MainAir zone during 10 summer days. Main air zone temperature (top) and relative humidity (bottom) during 10 summer days from the 15^th^ to the 25^th^ of July 2015 (black solid line: simulation | blue dotted line: “process” measurements *T*_*air,in*_ and *RH*_*air,in*_ (at the plant apex)). The blue area on the upper graph delimits the temperature vertical gradient as the difference between the minimum and maximum of *T*_*air,in,L*_, *T*_*air,in,M*_, *T*_*air,in*_ and *T*_*air,in,H*_.

Figs 8 and 9 illustrate the comparison between the *MainAir* zone simulated temperature and relative humidity and the measurements done during winter and summer days, considering a variables sampling frequency equal to the measurements one (5 min, refer to Table 1). Qualitatively, the model is able to reproduce the indoor temperature dynamics and amplitude variations. During winter nights (Fig 8), it outputs an air temperature quite close to *T*_*air,in*_ day after day, with a maximum difference that rarely exceeds 1°C. At that time, the crop is not yet mature (approximately 1 m height), there is obviously no solar gain, the roof vents are most of the time closed, the greenhouse is heated and the horizontal screens and the south wall curtain are unfolded. The indoor temperature is thus mainly driven by the indoor and outdoor convection (with possible condensation) and thermal radiation phenomena, as well as by the air exchange with the outside and the corridors. Between the sunrise and sunset, the temperature trends are also reproduced, although the model exhibits occasional offsets (e.g., the 11^th^ afternoon). Nevertheless, this is not systematic as shown on the 9^th^ and 17^th^. The measured vertical temperature gradient is depicted by the blue area, defining at each instant the temperature range in which all of the four measurements (*T*_*air,in,L*_, *T*_*air,in,M*_, *T*_*air,in*_ and *T*_*air,in,H*_) are included: it remained limited, with a maximum of 1.6 °C.

As far as the humidity is concerned, the trend is globally reproduced, albeit with less accuracy. The absolute difference is lower than 5%RH 71.4% of the time, and reaches a maximum of 16.7%RH the 11^th^ morning (at an air temperature close to 17 °C). It can also be noticed from the measurements that the day/night cycle is not clearly perceptible.

During summer, the dynamics are also reproduced, although, during the time frame of Fig 9, the simulated temperature is often higher than the measurements, but not always. At summer time, the roof vents are frequently used even at night and the shading screen is unfolded 15.5% of the daylight period: on Fig 9, it happened the 15^th^ (less than 4 hours) and the 16^th^ (almost 13 hours). Noticeable differences occur some days between 11:00–17:00. The highest one (reached the 16^th^) corresponds to a time period when the temperature vertical gradient is important at the top of the greenhouse. Values are close to 26–28 °C below 1.5 m (*T*_*air,in,L*_ and *T*_*air,in,M*_), 30 °C at 3.7 m (*T*_*air,in*_) and 35 °C at 4.7 m (*T*_*air,in,H*_), the latter had thus been very close to the simulation output. It is therefore not surprising that the perfectly stirred air volume approach is not able to reproduce precisely the indoor climate in such cases.

Regarding the relative humidity, the day/night cycles are more evident than in winter. The dynamics and the amplitude variations are globally reproduced: occasionally, the difference can be slightly higher than 20%RH. No obvious systematic offset can be observed.

Compared to the winter case, *RH*_*air,in*_ (and to a lesser extent *T*_*air,in*_) exhibits fluctuations during the day. The data analysis shows that it coincides with the use of the vents, especially when both leeward and windward ones are opened. Since for the present study, control loops are not modelled but vents actual feedbacks are used as inputs, the fact that the model does not reproduce the fluctuations cannot be attributed to difference regarding the vents control. The cause is probably related with the perfect stirred air volume modelling approach. However, this hypothesis cannot be confirmed as only one humidity measurement is available in the dataset.

These results being obtained without any calibration process (but with a crop yield model parameters values fitting), the global model accuracy could be improved in that way but this is not the purpose of this work. It would for instance make sense to adjust the ventilation functions retrieved from [56] (see subsection 3.4.1). Besides, deviations with the measurements can be imputable to hypotheses done regarding some inputs that where not recorded in the dataset (corridor air humidity, etc.).

The ability of a model to reproduce the indoor climate can be assessed quantitatively. Recently, [14] established a review of the metrics used in the literature for models assessment. They point out the diversity of the evaluation indicators, which makes the comparison between results difficult. Among them, the Root Mean Square Error (RMSE) and the Relative Root Mean Square Error (RRMSE) are nevertheless quite commonly used, although the latter should be avoided for non-absolute units like °C [14,26].

For the evaluation of her dynamic climate model of an unheated tomato greenhouse [76], relied on the literature to consider that a RMSE on the temperature lower than 1.6 °C is acceptable, and so did [26]. Among the recent studies reviewed by [14] providing temperature and/or humidity RMSE obtained for long-term simulations, two of them provide results which can be compared to:

- [77] based their work on Vanthoor’s [19] model to develop the “GreenLight” one implementing a detailed management of supplemental lighting. They evaluated it during a winter period (from the 20^th^ of October 2009 to the 9^th^ of February 2010, i.e., 112 days) in two Dutch tomato greenhouse 144 m^2^ compartments with 5 min time stamped measurements.
- [78] evaluated a model of a Chinese 875 m^2^ Venlo tomato greenhouse using data recorded in autumn and winter (5 months, 157 days) with a 5 min time step.

Table 3 shows the resulting cumulated RMSE and RRMSE at several simulation durations, using *T*_*air,in*_ and *RH*_*air,in*_ as references. The present model outputs indoor climate indicators that are considered satisfactory in the framework of the modelling objectives (the figures interpretation should be done keeping in mind the measurement uncertainty).

**Table 3:** Indicators computed during the first 112 days, 157 days and the whole production season (332 days, from the 03^rd^ of December 2014 to the 1^st^ of November 2015) with a 5 min sampling period, compared to studies [77] and [78].

Duration	112 days			157 days		332 days
Metric	[77] LED case	[77] HPS case	This study	[78]	This study	This study
RMSE (temperature)	1.74 °C	2.04 °C	0.9 °C	2.1 °C	1.1 °C	1.3 °C
RRMSE (temperature)	8.22%	9.77%	4.9%	–	5.7%	6.3%
RMSE (relative humidity)	5.52%RH	8.5%RH	6.5%RH	–	7.2%RH	8.1%RH
RRMSE (relative humidity)	6.57%	10.5%	8.9%	–	9.8%	10.9%
RMSE (absolute humidity)	–	–	1.2 g.m^-3^	1.68 g.m^-3^	1.6 g.m^-3^	2 g.m^-3^

**Fig 10:**
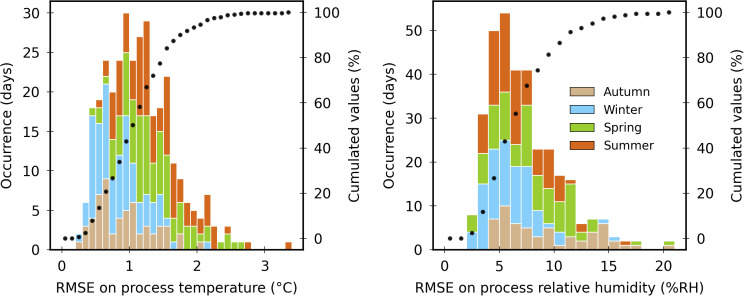
Distributions of the daily RMSE based on the process temperature (left) and relative humidity (right). Bars are colored according to the season of the day. The dotted curves correspond to the cumulated values (right axis of each figure).

Fig 10 illustrates the RSME distributions calculated each day (midnight to midnight): the daily temperature RMSE is lower than 1.6°C 84.1% of the cases, and the daily relative humidity RMSE is lower than 10%RH 81.4% of the time. The daily temperature RMSE is globally higher in spring and summer than in autumn and winter. It is particularly high a few days during the formers. Some of these occurrences are clearly associated with noticeable vertical temperature heterogeneities. For the other cases, no direct cause could have been attributed.

As far as the daily humidity RMSE is concerned, its distribution is quite balanced between winter, spring and summer. It exceeds 12%RH for 29 days, among which 17 are in the last month of the production season (October 2015): at that time, the uncertainty on the crop state is particularly high (refer to subsection 2.3.3) and may affect the transpiration.

### 4.3. Biological data

**Fig 11:**
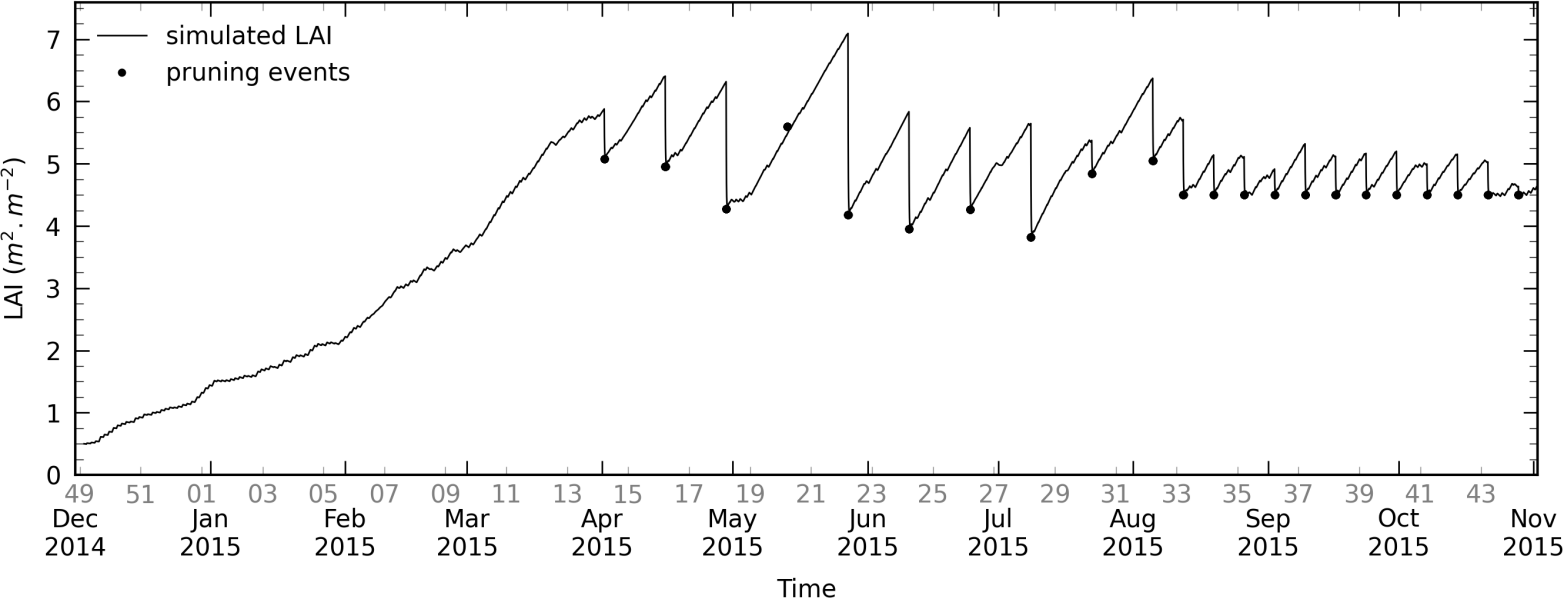
Simulated LAI over the whole production season (continuous line). Pruning events are shown by dots (starting week n°33 (2015), they have not been recorded and it is assumed that pruning was done every week at a target LAI of 4.5 m^2^ m^-2^). Calendar week number is shown in grey on the x-axis.

Fig 11 shows the LAI over the whole production season. Pruning is implemented as realistic discrete events, using the pruning dates and values as inputs (refer to subsection 2.3.3). The leaf development is not constant, i.e., the curve slope between two consecutive pruning events can significantly vary: the amount of carbohydrates allocated to the leaves development is indeed dependent on many factors as detailed in [25].

The model behaves as expected, since the simulated LAI remains higher than the pruning target value, with one exception. The fact that is it slightly lower to the latter week n°20 does not necessarily mean an underestimate: considering that the trajectory followed from the previous pruning (beginning of May) has a consistent slope, it most probably corresponds to a crop homogenization in the compartment rather than an actual pruning. However, the analysis is limited by the fact that the pruned leaves quantity is not included in the dataset. After the 5^th^ of August 2015, pruning events have not been recorded and a weekly basis at a target LAI of 4.5 m^2^ m^-2^ was assumed. In conjunction with the crop topping the 2^nd^ of September, this hypothesis may contribute, to some extent, to the relative humidity RMSE increasing during autumn 2015 as mentioned in subsection 4.2. In the framework of synergies studies, one can note that the pruning discrete management provides as a model output the flow of biomass, which could be used to produce biogas for example.

**Fig 12:**
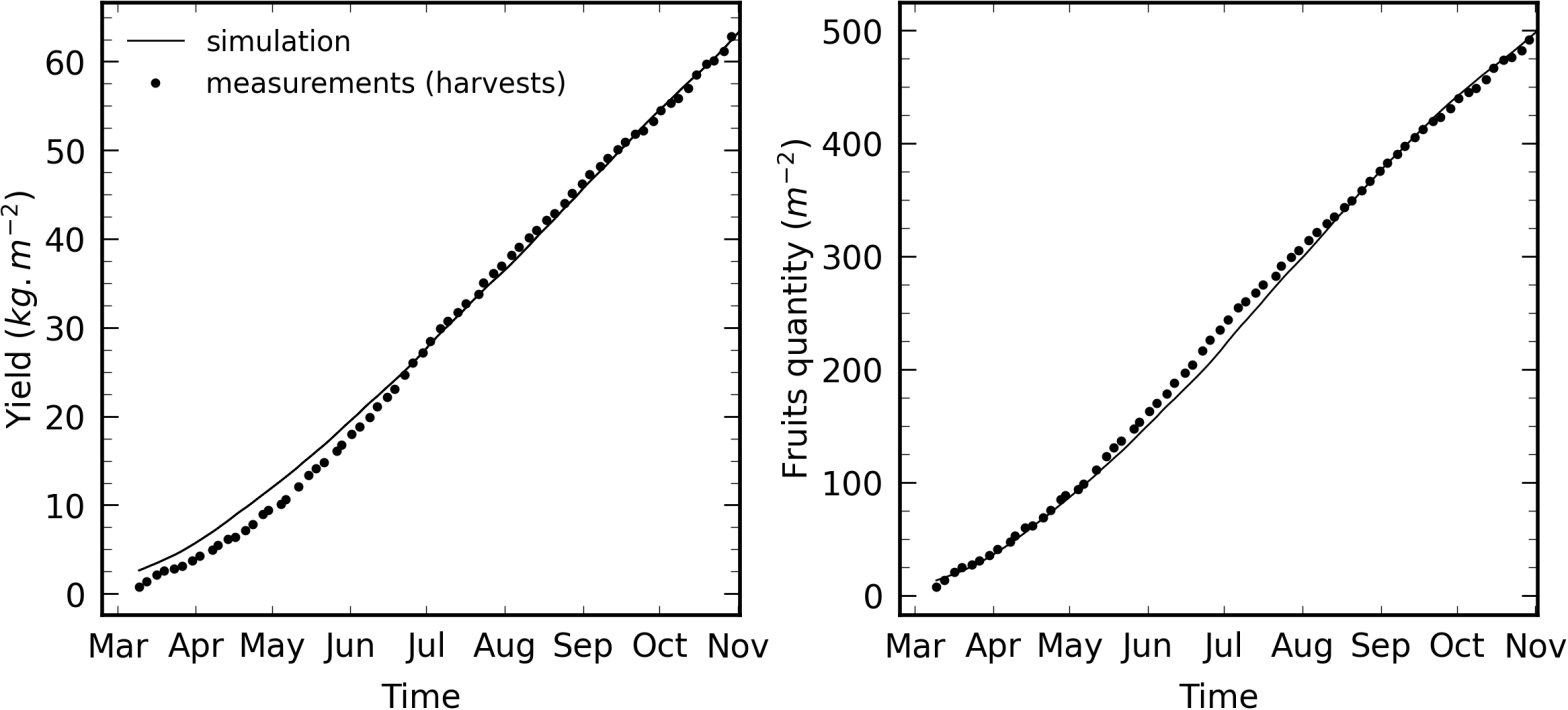
Simulated (solid line) and harvested (dots) tomato fresh weight yield (left) and fruits quantity (right). Values are given per cultivated area and cumulated over the whole production season.

Fig 12 shows the yield cumulated evolution (in kg of tomato per square meter of cultivated area) and the cumulated harvested tomato quantity (per square meter of cultivated area), compared with the actual measurements. As mentioned in subsection 3.7.1, the model outputs a Dry Matter mass allocated to the fruits, and a constant DM value of 5.4% was considered for the conversion into fresh fruits.

At the end of the year, the RMSE on the yield is 1.3 kg m^-2^ with a Mean Absolute Error (MAE) of 1.1 kg m^-2^. The simulated cumulated fruits quantity closely follows the measurements, with a final RMSE of 8.7 fruits m^-2^ and MAE of 6.5 fruits m^-2^.

During the spring period only, the simulated yield is slightly less accurate than the results obtained by [25] for the evaluation of their TYM applied to two Dutch Venlo greenhouses. It is however comparable to those of [79] for 98-days crop cycle period used to evaluate the Vanthoor’s [25] TYM in two Norway greenhouse compartments fitted with artificial lighting. The yield overestimate during spring (while the simulated harvested fruits quantity is very well reproduced) is probably partially imputable to seasonal DM variations in fruits. A more precise tuning of the crop yield model could be possible, as well as the implementation of such variations using for instance results from [74]. However, according to the authors, it would be a matter of “blind” calibration, since the DM content was not systematically measured at each harvest during the experiment in 2015. Besides, the measurement of the LAI before pruning is also not available in the dataset (nor the leaves DM contents). Consequently, the quantity of carbohydrates allocated by the model to the leaves cannot be specifically verified: yet, it influences the DM allocation to the fruits.

### 4.4. Mass balances

#### 4.4.1. Water.

At each simulation instant, a water mass balance is done using the model outputs:

- The cumulated transpired water mass is explicitly computed by the crop transpiration sub-model from [73]
- Considering a Dry Matter content between 7.5% and 13.6% for the stems and the leaves [75] and value of 5.4% for the fruits, the water content of the crop itself (stems, leaves and growing fruits), the harvested fruits and the pruned leaves can be estimated

During the time period when the net irrigation has been recorded (from the 8^th^ of December 2014 to the 26^th^ of October 2015), 897 m^3^ of water have been provided to the crop. According to the model, the total water consumption is included between 1014 and 1023 m^3^ depending on the considered Dry Matter content in the leaves and stems: at the whole production time scale, the model overestimates the water consumption by a ratio between 13.1 to 14.1%. However, at the last known pruning event (the 5^th^ of August 2015), the ratio is lower (8.8 to 9.7%): the final overestimation might thus be partially imputable to the hypothesis applied for the pruning practice after this date. In the present study, no attempt to reduce this deviation through a calibration of the transpiration sub-model was done. This perspective could be studied in a future work, as well as a comparison between transpiration sub-models available in the literature.

**Fig 13:**
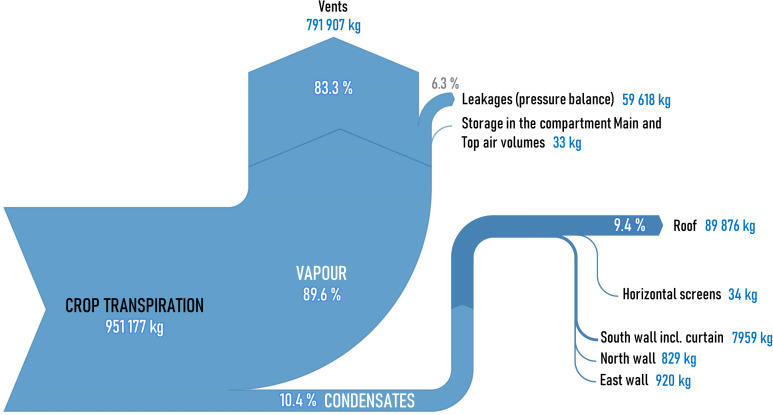
Simulated water flows between the 3^rd^ of December 2014 and the 1^st^ of November 2015. All values are rounded. Obviously, the model does not claim to reach a precision at the kg level, but this has been applied for this figure to illustrate the consideration of the condensation phenomena on the surfaces.

Fig 13 illustrates the water flows for a simulation between the 3^rd^ of December 2014 and the 1^st^ of November 2015. 10.4% of the transpired water is condensed, mainly on the roof and the south wall. No significant condensation (34 kg) occurred on the thermal nor on the shading screens, which is expected since it would mean unsuitable conditions in the compartment as liquid water could then fall on the crop and induce damages. Condensation on the east and north wall proved to be limited (920 kg and 829 kg): this is also expected because the corridors are heated, leading to wall average temperatures close to the MainAir zone one and thus above or slightly lower than the dew point. On the south side, 90% of the condensation occurs on the glass and 10% on the curtain: this is consistent with the in-situ observations during winter, and it tends to show that the hypotheses assumed regarding the water mass transfer through the curtain are satisfactory. The amount of water condensing on the south wall represents 8.9% of the roof condensation. The latter corresponds to a condensation flow of nearly 80.4 kg m_roof_^-2^ for 333 days (taking into account its slope): for the whole cover (roof + south wall), it corresponds to 75.5 kg m_cover_^-2^ for 333 days. Scaled to the whole year, it leads to 88 kg m_roof_^-2^ y^-1^ and 82.8 kg m_cover_^-2^ y^-1^. This is lower than the order of magnitude of 100 L m_cover_^-2^ y^-1^ reported by [80] for a typical greenhouse simulated with KASPRO [17]. However, in addition to local climate differences, it is reminded that the experimental compartment of the present study has some specificities (small floor area, corridors, adjoining compartment and curtain) which make the comparison with a typical use case inappropriate. It quantify the effect of taking into account the boundaries for water mass balance.

83.3% of the transpired water is lost at a vapor state through the roof vents, either when the vents are opened (natural ventilation) or closed (through the virtual height *h*_*vent,leak*_, refer to subsection 3.4.2). While the condensates on the roof are drained and already re-used, it brings out the water recovery potential of centralized ventilation systems (air handling units), as well as the associated latent heat. Finally, 6.3% of the water vapor is evacuated through the leakages caused by the pressure differences through the cover.

#### 4.4.2. Dry air and carbon dioxide.

Similar mass balances can be applied to the dry air and carbon dioxide flows. The corresponding results interpretation is, however, delicate due to the lack of information and airflows characterization of the greenhouse compartment and corridors.

Focusing on CO_2_, although injection measurements have been recorded, the fact that they are available weekly makes the analyses difficult: short dynamics (vents opening) and day/night cycles cannot be studied. Besides, the injection measurement accuracy was low, especially when the source was the gas boiler fumes.

At the last record point (the 12^th^ of October 2015, i.e., 313 days after the simulation start), the model underestimates the injected CO_2_ quantity by 17%. Any attempt to achieve a proper mass balance would necessitate:

- To check if the unique sensor is actually representative of the average concentration *C*_*CO2,air,in*_ in the compartment (homogeneity). Besides, it is worth noting that for the present study the sensor accuracy was approximately of 50 ppm: a sensitivity simulation considering a systematic error of only -20 ppm on the measurement reduces the underestimate by the model to 6.3%.
- A more accurate characterization of the air pressure in the compartment and in the corridors, since the pressure difference governs the airflow direction and amplitude.
- An outdoor CO_2_ concentration measurement. To the best knowledge of the authors, dynamic greenhouse models usually consider atmospheric values [17,42,81]. However, for the present greenhouse, this hypothesis may not be applicable: as shown by [82], atmospheric measurements are not necessarily adapted to assess the outdoor CO_2_ concentration close to the ground in urban and sub-urban areas. In their study, they reported variations higher than 100 ppm in Salt Lake County (USA) depending on the season and location.

### 4.5. Discussion about the confidence in models and level of detail

**Fig 14:**
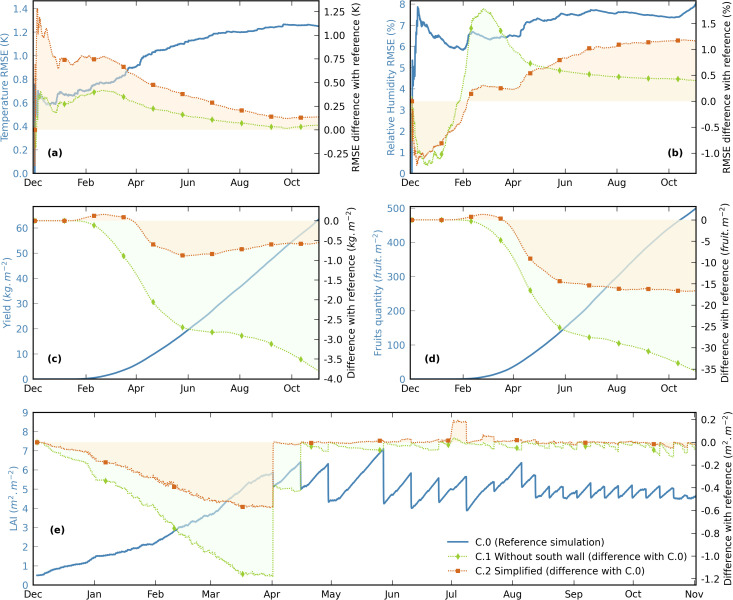
Comparison of model variants. For each of the five graphs, the reference model output trajectory *x*_*ref*_ is plotted using the left y-axis in blue, and the comparison with the variants are shown as the difference (*x*_*var*_ *– x*_*ref*_) in the same units on the right y-axis. **(a)** cumulated *MainAir* zone temperature RMSE (K) | **(b)** cumulated *MainAir* zone relative humidity RMSE (%) | **(c)** yield (kg m^-2^) | **(d)** harvested fruits quantity (fruit m^-2^) | (e) LAI (m^2^ m^-2^).

The present work was motivated by 1) the need to apply an approach that could be as generic as possible for prospective studies 2) the difficulties to manage boundaries effects 3) the difficulties to justify the use of parameter values taken from the literature. From the authors point of view, using sub-models that rely more on physical properties (such as greenhouse structural elements dimensions for the direct light transmission model in [16], or equivalent leakage areas for the infiltrations) is a mean to increase the confidence one can attribute to simulation results. It also has the advantage of exposing parameters that are easier to manage when a phenomenological global model is simplified or used as a based to derive black-box ones. Some trivial considerations (such as discrete-event pruning) can be so easily implemented that it is not required to demonstrate their interest. On the other hand, increasing the model “complexity” shall be balanced by the effects it actually has on the results. In this section, two global model variants are used to illustrate different aspects and related results are shown on Fig 14, compared to reference model:

- Case 1 (C.1): the south gable boundary is ignored (i.e., the south wall sub-model is removed)
- Case 2 (C.2): all the walls are removed, the alleyways are ignored (i.e., *r*_*cul*_ *=* 1) and the cover transmittance is assumed constant over the year, without distinguishing direct and diffuse parts. For the latter, an arbitrary value had to be chosen so that it does not overestimate the effects of using a more detailed approach. Within the a priori realistic range of [0.7:0.8] (-), a parameter sweeping shows that a constant transmittance of 0.78 (from Vanthoor [42], which results of measurements in Dutch greenhouses) leads to almost minimize the sum of the cumulative temperature and relative humidity RMSE for a C.2 configuration. This value is considered: the bias is consequently favorable to the C.2 configuration compared with the detailed approach.

All these cases are simulated at equivalent energy use.

Modelling the compartment without its south wall globally increases the cumulated temperature RMSE (Fig 14a, green curve) in winter and spring (with a maximum of + 0.42 K. Before February, the relative humidity RMSE (Fig 14b) is lower than the reference simulation, but it becomes noticeably higher up to May. It illustrates the fact that indicators minimization is not always a way to increase the “accuracy” of a model. Limited to a single criterion on the relative humidity and if the observation period were limited to the first months, it would mean that removing the south wall leads to a better model: this is from a physics point of view questionable. The effects on the crop are stronger: the yield is reduced from −3.8 kg m^-2^ (Fig 14c), the fruits quantity of −35 fruits m^-2^ (Fig 14d). The LAI growth is so decreased (−1.2 m^2^ m^-2^ at the end of March, Fig 14e) that it misses the first pruning event.

The second studied case (C.2, in orange on Fig 14) is close to a “quasi-infinite greenhouse approach”. Although the temperature RMSE trajectory is higher than the results of the reference approach (Fig 14a), the difference is quite unexpectedly low (it is however reminded that the cover transmittance has been chosen beforehand to minimize the RMSE). Nevertheless, the difference in terms of relative humidity is noticeable (Fig 14b). Interestingly, the effects on the Yield (Fig 14c) and the LAI (Fig 14e) are less pronounced than for C.1. In such highly coupled modelling applications, the integration in a global model of more physical considerations could lead to increase the global deviation computed statistically. In C.2, the underestimate of solar radiation intercepted by the crop due to the south wall missing part is partially balanced by the overestimate of the surface occupied by the crop in the compartment itself since *r*_*cul*_ is set to 1. Yet, a model which takes into account the presence of alleyways is more accurate, all the more so since *r*_*cul*_ value is known with a high confidence as it only depends on geometrical considerations. The necessity to model the corridors walls is a priori less obvious, since the air temperature differences across them are much lower than with outdoor and because they are never directly exposed to sunlight. Still, physic-based considerations have to be considered regarding the infiltrations, because the air properties in the corridors (temperature, gas contents) are not the same as the outdoor air ones. Using air leakage laws such as those based on wind-speed does not help to know with which volume (corridor and outdoor) the exchanges happen. Moreover, the evaluation of the integration of HVAC systems can affect the neutral levels, thus those air balances.

According to the authors, focusing on indicators minimization does not necessarily lead to a more representative global model when a physic-based approach is used. Firstly, if the evaluation of more detailed models provides unexpectedly worst statistical indicators values, it could instead highlight opportunities to increase the associated knowledge: other unidentified phenomena may unexpectedly be involved. As well, small physic-based considerations can change the sensitivity of some parameters, helping in “denoising” their respective effects and thus contribute to derive more precise black-box models from the global phenomenological one. Secondly, it is worth reminding that for applications such as greenhouses, the evaluation of the deviations highly depends on the measurements accuracy and representativeness: it implies asymptotic values that statistics indicators could never reach. It is believed that in the perspective of model applications for prospective evaluation, adapting modelling approaches from physics-based considerations is a way to increase the confidence one can attribute to results, providing it does not require additional hypotheses or parameters that are difficult to assess.

## 5. Conclusion

In the perspective of the evaluation of various combinations of solutions to the challenges that greenhouse producers are facing with, greenhouse climate and energy modelling can be used: however, the modelling approach shall be appropriate. Starting from the dynamic model in [42] and its implementation by [23], contributions have been presented:

1) Emphasis was placed on developing generic sub-models which can be configured based on physical properties instead of calibration. This modular implementation also aimed to facilitate the reuse of components in a context where non-standard greenhouse elements have to be assessed, as well as to derive benefits from available implementations in other domains.
2) Solar gains are computed with a more detailed approach, taking into account the cover geometry as well as the solar irradiance nature depending on the instantaneous weather.
3) Boundary effects are considered to manage the gap between existing large-scale greenhouses dynamic models and applications such as experimental facilities or urban farming.
4) Inside the greenhouse, explicit moist air energy and mass balances in dry air and CO_2_ are implemented in addition to the water vapor one. To model air leakages, the present work suggests the use of pressure differences between volumes and the definition of an equivalent vents leakage height.
5) As for thermal radiation, a methodology mixing analytical and numerical approach is applied to compute the view factors.
6) The Vanthoor’s [25] tomato crop yield model is now able to manage discrete pruning events as an accurate picture of the situation: its parameters have been adapted to the cultivar and cultivation practices, thus constituting an instantiable reference for any future greenhouse concept evaluation.

This modelling approach was assessed using an extensive dataset from an experimental 1037 m^3^ soil-less tomato greenhouse. Without a calibration process, the corresponding global model is able to reproduce the indoor climate at both small and large time scales. Improvement perspectives are identified. Regarding thermodynamics for instance, the “perfectly stirred tank” approach is sometimes limited in greenhouses where noticeable gradients (temperature, humidity, CO_2_) can occur: co-simulation between Modelica and Fast Fluid Dynamics (FFD) [83] could therefore be investigated not only to manage indoor climate gradient, but also convective heat transfers dependency on varying local air speed.

This generic and modular modelling approach can be applied to a wide range of practical applications. It can handle existing commercial greenhouse configurations as long as their location and characteristics are known. Since it is not limited to semi-infinite greenhouses, it is also well suited for small urban greenhouses where boundary conditions play a significant role. In addition, it facilitates the assessment of emerging technical solutions and materials and allows for the analysis of climate change impacts by incorporating meteorological conditions more thoroughly. Moreover, since the global model is hybrid (dynamic, continuous and handling discrete events), complete control (PID, Sequential Functions Charts) can be integrated and more complex ones could be assessed: optimal control is also a lead towards the reduction of the greenhouses environmental footprint. Finally, the resulting global phenomenological model can be used through the generic and modular approach to evaluate prospective sustainable solutions. It also constitutes a reference from which alternative models can be built (simplified ones, black-box) for practical applications such as predictive control.

### Nomenclature

**Table:** 

Acronyms	
CAMS	Copernicus Atmosphere Monitoring Service
CTIFL	*Centre Technique Interprofessionnel des Fruits et Légumes*
DM	Dry Matter
FOS	Free and Open-Source
HPS	High Pressure Sodium
HVAC	Heating, Ventilation and Air Conditioning
LAI	Leaf Area Index (m_leaf_^2^ m_floor_^-2^)
LED	Light Emitting Diode
MAE	Mean Absolute Error
NIR	Near Infra Red
PAR	Photosynthetically Active Radiation
RMSE	Root Mean Square Error
RRMSE	Relative Root Mean Square Error
TYM	Tomato Yield Model
UV	Ultra Violet
General notations	
*A*	Surface (m^2^)
C	Concentration (ppm)
F	Airflow (m^3^ s^-1^ or m^3^ s^-1^ m^-2^ depending on the context)
FF	View factor (-)
g	Acceleration of gravity (9.81 m s^-2^)
G	Ventilation function, for roof vents (-)
h	Specific enthalpy (for an air volume) (J kg_dAir_^-1^)
H	Enthalpy (J)
I	Irradiance (W m^-2^)
k	Coefficient (contextual)
K	Extinction coefficient (-)
l	Length (m)
M	Mass (kg)
p	Pressure (absolute or partial) (Pa)
$\dot{Q}$	Heat transfer (W)
RH	Air relative humidity (%1)
T	Temperature (K or °C)
U	Internal energy (J)
V	Volume (m^3^)
Specific notations	
Ce	Cloud ratio (-)
C_f_	Discharge coefficient (-)
Cp	Specific heat capacity (J K^-1^ kg^-1^)
H_o_	Roof vent height (m)
h_vent,leak_	Virtual roof vent opening height (mm)
I_glob_	Outside solar global horizontal irradiance (W m^-2^)
I_lw,Sky-Pyr_	Net longwave irradiance between the sky and the pyrgeometer (W m^-2^)
L(α,γ)	Radiance of a sky element located at the azimuth *α* and zenith angle *γ* (W m^-2^ sr^-1^)
L_z_	Radiance at the zenith (W m^-2^ sr^-1^)
L’(α,γ)	Relative radiance of a sky element located at the azimuth *α* and zenith angle *γ* (-)
L_o_	Roof vent length (m)
N_L_	Number of leeward roof vents
N_W_	Number of windward roof vents
r_cul_	Ratio of the actual cultivated area over the total floor area (-)
U_wind,8m_	Wind speed (8 m from the ground) (m s^-1^)
x_w_	Air humidity ratio (kg_w_ kg_dAir_^-1^)
ΔP	Pressure difference (Pa)
Greek letters and other symbols	
α	Azimuth (rad or °)
γ	Zenith angle (rad or °)
δ	Roof vent opening angle (°)
ε	Emissivity (-)
η	Flow coefficient (m^3^ s^-1^ Pa^-…^)
ϱ	Density (kg m^-3^)
ρ	Reflectance (-)
σ	Stefan-Boltzmann constant (5.67·10^−8^ W m^-2^ K^-4^)
τ	Transmittance (-)
ψ	Slope (°)
M	Molar mass (kg mol^-1^)
Subscript	
air	Air (medium)
alw	Alleyways
b	Greenhouse roof bars set
buoy	For buoyancy effect
Can	Canopy
crack	For an airflow through a crack
Crop	When applicable to the crop/ plant
cul	When applicable to the actual cultivated area in the greenhouse
dAir	Dry air
dif	Diffuse (for irradiance)
dir	Direct (for irradiance)
g	Glass
i	Component (gas)
in	Indoor
L	When applicable to a leeward roof vent
MainAir	When related to the Main air zone, below the screens system
Pyr	Pyrgeometer
r	Greenhouse roof
rg	Greenhouse roof ridges and gutters set
out	Outdoor/ outside values
s	Sun
Sky	Sky/ atmosphere
TopAir	When related to the Top air zone, above the screens system
vent	Roof vents
w	Water
W	Windward roof vent
wind	Wind

## Supporting information

S1 File Modelled greenhouse compartment details and model parametrization. This file includes additional details about the modelled greenhouse compartment, and provides the corresponding parameter values and related explanations.
(DOCX)

S2 File Dataset of the global model inputs and outputs presented in this paper. This dataset includes all the modelled greenhouse compartment experimental inputs during the whole simulation, as well as the model outputs presented and discussed in this paper. This supporting information is available from https://doi.org/10.5281/zenodo.14943558.
(DOCX)

S3 File Detailed diagram of the Vanthoor’s tomato yield model. This file is a graphical representation of the Vanthoor’s tomato yield model. The model description and nomenclature can be found in the author work [25,42].
(JPG)

S4 File High resolution for Figs 8 and 9. This file includes high-resolution version of Figs 8 and 9.
(ZIP)

S5 File Modelica models to reproduce the paper results. This supporting information is available from https://doi.org/10.5281/zenodo.15590403.
(DOCX)

## References

[1] Teitel M, Liang H, Vitoshkin H, Tanny J, Ozer S. Airflow patterns and turbulence characteristics above the canopy of a tomato crop in a roof-ventilated insect-proof screenhouse. Biosyst Eng. 2020;190:184–200. doi: 10.1016/j.biosystemseng.2019.12.001

[2] Montero JI, Teitel M, Baeza E, Lopez JC, Kacira M. Greenhouse design and covering materials. In: Baudoin W, Nono-Womdim R, Lutaladio N, Hodder A, Castilla N, Leonardi C, et al., editors. Good agricultural practices for greenhouse vegetable crops: principles for Mediterranean climate areas. Rome: Food and Agricultural Organization of the United Nations (FAO); 2013. p. 35–62. Available from: https://www.fao.org/3/i3284e/i3284e.pdf

[3] Boulard T, Raeppel C, Brun R, Lecompte F, Hayer F, Carmassi G, et al. Environmental impact of greenhouse tomato production in France. Agron Sustain Dev. 2011;31(4):757–77. doi: 10.1007/s13593-011-0031-3

[4] Bartzanas T, Tchamitchian M, Kittas C. Influence of the Heating Method on Greenhouse Microclimate and Energy Consumption. Biosyst Eng. 2005;91(4):487–99. doi: 10.1016/j.biosystemseng.2005.04.012

[5] Körner O, Challa H. Process-based humidity control regime for greenhouse crops. Comput Electron Agricult. 2003;39(3):173–92. doi: 10.1016/s0168-1699(03)00079-6

[6] De Gelder A, Dieleman JA, Bot GPA, Marcelis LFM. An overview of climate and crop yield in closed greenhouses. J Horticult Sci Biotechnol. 2012;87(3):193–202. doi: 10.1080/14620316.2012.11512852

[7] IPCC. 6th assessment report - Fact sheet Europe. Intergovernental Panel on Climate Change; 2022. Report No.: 6. Available from: https://www.ipcc.ch/report/ar6/wg2/downloads/outreach/IPCC_AR6_WGII_FactSheet_Europe.pdf

[8] Paris B, Vandorou F, Balafoutis AT, Vaiopoulos K, Kyriakarakos G, Manolakos D, et al. Energy Use in Greenhouses in the EU: A Review Recommending Energy Efficiency Measures and Renewable Energy Sources Adoption. Appl Sci. 2022;12(10):5150. doi: 10.3390/app12105150

[9] Grisey A, Brajeul E, Oudin T. Enquête 2021 auprès des producteurs de tomate et de concombre sous serre sur l’utilisation de l’énergie. 2022. p. 32–6. Report No.: 385.

[10] Nikolaou G, Neocleous D, Katsoulas N, Kittas C. Irrigation of Greenhouse Crops. Horticulturae. 2019;5(1):7. doi: 10.3390/horticulturae5010007

[11] Dossa-Thauvin V. Évolutions de la ressource en eau renouvelable en France métropolitaine de 1990 à 2018. Paris: Service des données et études statistiques (SDES), Ministère de la Transition Ecologique et de la Cohésion des Territoires; 2022. Available from: https://side.developpement-durable.gouv.fr/default/digitalCollection/

[12] Chauveau M, Chazot S, Perrin C, Bourgin P-Y, Sauquet E, Vidal J-P, et al. Quels impacts des changements climatiques sur les eaux de surface en France à l’horizon 2070 ? La Houille Blanche. 2013;99(4):5–15. doi: 10.1051/lhb/2013027

[13] Sethi VP, Sumathy K, Lee C, Pal DS. Thermal modeling aspects of solar greenhouse microclimate control: A review on heating technologies. Solar Energy. 2013;96:56–82. doi: 10.1016/j.solener.2013.06.034

[14] Katzin D, van Henten EJ, van Mourik S. Process-based greenhouse climate models: Genealogy, current status, and future directions. Agricultural Syst. 2022;198:103388. doi: 10.1016/j.agsy.2022.103388

[15] López-Cruz IL, Fitz-Rodríguez E, Salazar-Moreno R, Rojano-Aguilar A, Kacira M. Development and analysis of dynamical mathematical models of greenhouse climate: A review. Europ J Hortic Sci. 2018;83(5):269–79. doi: 10.17660/ejhs.2018/83.5.1

[16] Bot GPA. Greenhouse climate from physical processes to a dynamic model. Doctoral dissertation, Wageningen University. 1983. Available from: https://edepot.wur.nl/188427

[17] De Zwart HF. Analyzing energy-saving options in greenhouse cultivation using a simulation model. Doctoral dissertation, Wageningen University. 1996. Available from: https://research.wur.nl/en/publications/analyzing-energy-saving-options-in-greenhouse-cultivation-using-a-2

[18] Pieters JG, Deltour JM, Debruyckere MJ. Condensation and dynamic heat transfer in greenhouses part I: theoretical model. Int Agric Eng J. 1996;5: 119–133. Available from: http://hdl.handle.net/1854/LU-266295

[19] Vanthoor BHE, Stanghellini C, van Henten EJ, de Visser PHB. A methodology for model-based greenhouse design: Part 1, a greenhouse climate model for a broad range of designs and climates. Biosyst Eng. 2011;110(4):363–77. doi: 10.1016/j.biosystemseng.2011.06.001

[20] Choab N, Allouhi A, El Maakoul A, Kousksou T, Saadeddine S, Jamil A. Review on greenhouse microclimate and application: Design parameters, thermal modeling and simulation, climate controlling technologies. Solar Energy. 2019;191:109–37. doi: 10.1016/j.solener.2019.08.042

[21] Rodríguez F, Yebra LJ, Berenguel M, Dormido S. Modelling and simulation of greenhouse climate using Dymola. IFAC Proc Volume. 2002;35(1):79–84. doi: 10.3182/20020721-6-es-1901.01322

[22] Schwan T, Unger R, Pipiorke J. Energy-efficient design of a research greenhouse with Modelica. Proceedings of the 11th International Modelica Conference, Versailles, France, September 21-23, 2015. Linköping University Electronic Press; 2015. p. 207–216. doi: 10.3384/ecp15118207

[23] Altes-Buch Q, Quoilin S, Lemort V. Greenhouses: A Modelica Library for the Simulation of Greenhouse Climate and Energy Systems. In: Proceedings of the 13th International Modelica Conference, Regensburg, Germany, 2019. p. 533–42. doi: 10.3384/ecp19157533

[24] Altes-Buch Q, Quoilin S, Lemort V. A modeling framework for the integration of electrical and thermal energy systems in greenhouses. Build Simul. 2021;15(5):779–97. doi: 10.1007/s12273-021-0851-2

[25] Vanthoor BHE, de Visser PHB, Stanghellini C, van Henten EJ. A methodology for model-based greenhouse design: Part 2, description and validation of a tomato yield model. Biosyst Eng. 2011;110(4):378–95. doi: 10.1016/j.biosystemseng.2011.08.005

[26] Piché P. Amélioration du comportement thermique d’une serre nordique communautaire. Doctoral dissertation, Université de Pau et des Pays de l’Adour. 2021. Available from: https://tel.archives-ouvertes.fr/tel-03368948

[27] Marin JG. Greenhouse Tomatoes: Process Simulation. Master’s thesis, University of Arkansas. 2021. Available from: https://scholarworks.uark.edu/etd/4256

[28] Maureira F, Rajagopalan K, Stöckle CO. Evaluating tomato production in open-field and high-tech greenhouse systems. J Clean Prod. 2022;337:130459. doi: 10.1016/j.jclepro.2022.130459

[29] Blaud PC, Haurant P, Chevrel P, Claveau F, Mouraud A. Multi-flow optimization of a greenhouse system: A hierarchical control approach. Appl Energy. 2023;351:121840. doi: 10.1016/j.apenergy.2023.121840

[30] Mattsson SE, Elmqvist H. An overview of the modeling language modelica. Helsinki, Finland; 1998. Available from: https://modelica.org/publications/papers/Eurosim98Modelica.pdf

[31] Fritzson P. Principles of object-oriented modeling and simulation with Modelica 3.3: a cyber-physical approach. 2 ed. Piscataway, NJ: IEEE Press. 2015.

[32] Haumer A, editor. Proceedings of the 13th International Modelica Conference. Proceedings of the 13th International Modelica Conference. Regensburg, Germany; 2019. doi: 10.3384/ecp19157

[33] Wetter M, Zuo W, Nouidui TS, Pang X. Modelica Buildings library. J Build Perform Simul. 2013;7(4):253–70. doi: 10.1080/19401493.2013.765506

[34] Dassault Systèmes. Dymola [Software]. 2023. Available from: https://www.3ds.com/products-services/catia/products/dymola/

[35] Fritzson P, Pop A, Abdelhak K, Ashgar A, Bachmann B, Braun W, et al. The OpenModelica Integrated Environment for Modeling, Simulation, and Model-Based Development. MIC. 2020;41(4):241–95. doi: 10.4173/mic.2020.4.1

[36] Petzold LR. A description of DASSL: a differential/algebraric system solver. Livermore, California: Sandia National Laboratories; 1982. Report No.: SAND82-8637. Available from: https://www.osti.gov/servlets/purl/5882821

[37] Cohen SD, Hindmarsh AC, Dubois PF. CVODE, A Stiff/Nonstiff ODE Solver in C. Comput Phys. 1996;10(2):138–43. doi: 10.1063/1.4822377

[38] Copernicus. Copernicus Atmosphere Monitoring Services (CAMS) Atmosphere Data Store (ADS). In: CAMS solar radiation time-series [Internet]. 2021 [cited 17 Nov 2021]. Available from: https://ads.atmosphere.copernicus.eu/cdsapp#!/dataset/cams-solar-radiation-timeseries?tab=overview

[39] Qu Z, Oumbe A, Blanc P, Espinar B, Gesell G, Gschwind B, et al. Fast radiative transfer parameterisation for assessing the surface solar irradiance: The Heliosat‑4 method. metz. 2017;26(1):33–57. doi: 10.1127/metz/2016/0781

[40] Schroedter-Homscheidt M, Azam F, Betcke J, Hanrieder N, Lefèvre M, Saboret L, et al. Surface solar irradiation retrieval from MSG/SEVIRI based on APOLLO Next Generation and HELIOSAT‑4 methods. metz. 2022;31(6):455–76. doi: 10.1127/metz/2022/1132

[41] Igawa N, Koga Y, Matsuzawa T, Nakamura H. Models of sky radiance distribution and sky luminance distribution. Solar Energy. 2004;77(2):137–57. doi: 10.1016/j.solener.2004.04.016

[42] Vanthoor BHE. A model-based greenhouse design method. Doctoral dissertation, Wageningen University. 2011. Available from: https://edepot.wur.nl/170301

[43] Sourisseau S, Toublanc C, Chantoiseau E, Havet M. Assessment of technical innovations for greenhouses: modelling of a horizontal two-screens system. Acta Hortic. 2023;(1377):127–36. doi: 10.17660/actahortic.2023.1377.15

[44] Baeza E, Hemming S, Stanghellini C. Materials with switchable radiometric properties: Could they become the perfect greenhouse cover? Biosyst Eng. 2020;193:157–73. doi: 10.1016/j.biosystemseng.2020.02.012

[45] Stanghellini C, Dai J, Kempkes F. Effect of near-infrared-radiation reflective screen materials on ventilation requirement, crop transpiration and water use efficiency of a greenhouse rose crop. Biosyst Eng. 2011;110(3):261–71. doi: 10.1016/j.biosystemseng.2011.08.002

[46] Goudriaan J. Crop micrometeorology: a simulation study. Doctoral dissertation, Wageningen University. 1977. Available from: https://edepot.wur.nl/166537

[47] Thomas C, Wandji Nyamsi W, Arola A, Pfeifroth U, Trentmann J, Dorling S, et al. Smart Approaches for Evaluating Photosynthetically Active Radiation at Various Stations Based on MSG Prime Satellite Imagery. Atmosphere. 2023;14(8):1259. doi: 10.3390/atmos14081259

[48] Torres JL, Torres LM. Angular Distribution of Sky Diffuse Radiance and Luminance. Modeling solar radiation at the earth’s surface: recent advances. Heidelberg: Springer; 2008. p. 427–448. Available from: https://link.springer.com/chapter/10.1007/978-3-540-77455-6_17

[49] De Zwart HF. Determination of Direct Transmission of a Multispan Greenhouse Using Vector Algebra. J Agricul Eng Res. 1993;56(1):39–49. doi: 10.1006/jaer.1993.1059

[50] Kotilainen T, Robson TM, Hernández R. Light quality characterization under climate screens and shade nets for controlled-environment agriculture. PLoS One. 2018;13(6):e0199628. doi: 10.1371/journal.pone.0199628 29940006 PMC6016941

[51] Wetter M. Simulation-Based Building Energy Optimization. Doctoral dissertation, University of California, Berkeley. 2004. Available from: https://simulationresearch.lbl.gov/wetter/download/mwdiss.pdf

[52] ASHRAE. 2013 ASHRAE handbook: fundamentals. SI edition. Atlanta, GA; 2013.

[53] Boulard T, Baille A. Modelling of Air Exchange Rate in a Greenhouse Equipped with Continuous Roof Vents. J Agricult Eng Res. 1995;61(1):37–47. doi: 10.1006/jaer.1995.1028

[54] Roy J-C, Boulard T, Kittas C, Wang S. PA—Precision Agriculture: Convective and Ventilation Transfers in Greenhouses, Part 1: the Greenhouse considered as a Perfectly Stirred Tank. Biosystems Eng. 2002;83: 1–20. doi: 10.1006/bioe.2002.0107

[55] Papadakis G, Mermier M, Meneses JF, Boulard T. Measurement and Analysis of Air Exchange Rates in a Greenhouse with Continuous Roof and Side Openings. J Agricult Eng Res. 1996;63(3):219–27. doi: 10.1006/jaer.1996.0023

[56] De Jong T. Natural ventilation of large multi-span greenhouses. Doctoral dissertation, Wageningen University. 1990. Available from: https://library.wur.nl/WebQuery/wurpubs/fulltext/206452

[57] Brinks P, Kornadt O, Oly R. Air infiltration assessment for industrial buildings. Energy Build. 2015;86:663–76. doi: 10.1016/j.enbuild.2014.10.040

[58] Kuroyanagi T. Investigating air leakage and wind pressure coefficients of single-span plastic greenhouses using computational fluid dynamics. Biosyst Eng. 2017;163:15–27. doi: 10.1016/j.biosystemseng.2017.08.004

[59] Von Elsner B, Briassoulis D, Waaijenberg D, Mistriotis A, von Zabeltitz Chr, Gratraud J, et al. Review of Structural and Functional Characteristics of Greenhouses in European Union Countries: Part I, Design Requirements. J Agricult Eng Res. 2000;75(1):1–16. doi: 10.1006/jaer.1999.0502

[60] Wells DA, Hoxey RP. Measurements of wind loads on full-scale glasshouses. J Wind Eng Ind Aerodynam. 1980;6(1–2):139–67. doi: 10.1016/0167-6105(80)90027-6

[61] Boulard T, Roy J-C, Pouillard J-B, Fatnassi H, Grisey A. Modelling of micrometeorology, canopy transpiration and photosynthesis in a closed greenhouse using computational fluid dynamics. Biosyst Eng. 2017;158:110–33. doi: 10.1016/j.biosystemseng.2017.04.001

[62] Hemming S, Baeza E, Mohammadkhani V, Van Breugel B. Energy saving screen materials: measurement method of radiation exchange, air permeability and humidity transport and a calculation method for energy saving. Bleiswijk: Wageningen University & Research, BU Greenhouse Horticulture; 2017. doi: 10.18174/409298

[63] Awbi HB, Hatton A. Natural convection from heated room surfaces. Energy Build. 1999;30(3):233–44. doi: 10.1016/s0378-7788(99)00004-3

[64] Miguel AF, van de Braak NJ, Silva AM, Bot GPA. Free-Convection Heat Transfer in Screened Greenhouses. J Agricult Eng Res. 1998;69(2):133–9. doi: 10.1006/jaer.1997.0235

[65] Piscia D, Muñoz P, Panadès C, Montero JI. A method of coupling CFD and energy balance simulations to study humidity control in unheated greenhouses. Comput Electron Agricult. 2015;115:129–41. doi: 10.1016/j.compag.2015.05.005

[66] Hollands KGT, Raithby GD, Konicek L. Correlation equations for free convection heat transfer in horizontal layers of air and water. Int J Heat Mass Trans. 1975;18(7–8):879–84. doi: 10.1016/0017-9310(75)90179-9

[67] EnergyPlus. EnergyPlus documentation. [Software]. US DOE; 2021. Available from: https://energyplus.net/documentation

[68] Sourisseau S, Chantoiseau E, Toublanc C, Havet M. Computing radiative heat transfers in greenhouses: a methodology coupling analytical and numerical approaches for view factors assessment. Acta Hortic. 2025;(1426):1–8. doi: 10.17660/actahortic.2025.1426.1

[69] Sourisseau S, Chantoiseau E, Toublanc C, Havet M. Implementation of view factors computation using a discretized approach based on PyVista and PyViewFactor, applied to a greenhouse study case with numerous obstacles. [Software]. 2023. Available from: 10.5281/zenodo.10020604

[70] Bogdan M, Walther E, Alecian M, Chapon M. Calcul des facteurs de forme entre polygones – Application à la thermique urbaine et aux études de confort. Châlons en Champagne, France; 2022. p. 1–8. Available from: https://lhypercube.arep.fr/references/publications/ibpsa_fr_22/

[71] Sullivan C, Kaszynski A. PyVista: 3D plotting and mesh analysis through a streamlined interface for the Visualization Toolkit (VTK). JOSS. 2019;4(37):1450. doi: 10.21105/joss.01450

[72] Mazumder S, Ravishankar M. General procedure for calculation of diffuse view factors between arbitrary planar polygons. Int J Heat Mass Trans. 2012;55(23–24):7330–5. doi: 10.1016/j.ijheatmasstransfer.2012.07.066

[73] Stanghellini C. Transpiration of greenhouse crops: an aid to climate management. Doctoral dissertation, Wageningen University. 1987. Available from: https://edepot.wur.nl/202121

[74] De Koning ANM. Development and dry matter distribution in glasshouse tomato: a quantitative approach. Doctoral dissertation, Wageningen University. 1994. Available from: https://research.wur.nl/en/publications/development-and-dry-matter-distribution-in-glasshouse-tomato-a-qu

[75] Heuvelink E. Tomato growth and yield: quantitative analysis and synthesis. Doctoral dissertation, Wageningen University. 1996. Available from: https://edepot.wur.nl/206832

[76] Baptista FJ. Modelling the Climate in Unheated Tomato Greenhouses and Predicting Botrytis cinerea Infection. Doctoral dissertation, Universidade de Evora. 2007. Available from: https://dspace.uevora.pt/rdpc/bitstream/10174/1724/1/PhD%20Thesis_FBaptista2007.pdf

[77] Katzin D, van Mourik S, Kempkes F, van Henten EJ. GreenLight – An open source model for greenhouses with supplemental lighting: Evaluation of heat requirements under LED and HPS lamps. Biosyst Eng. 2020;194:61–81. doi: 10.1016/j.biosystemseng.2020.03.010

[78] Su Y, Xu L, Goodman ED. Nearly dynamic programming NN‐approximation–based optimal control for greenhouse climate: A simulation study. Optim Control Appl Methods. 2017;39(2):638–62. doi: 10.1002/oca.2370

[79] Righini I, Vanthoor B, Verheul MJ, Naseer M, Maessen H, Persson T, et al. A greenhouse climate-yield model focussing on additional light, heat harvesting and its validation. Biosyst Eng. 2020;194:1–15. doi: 10.1016/j.biosystemseng.2020.03.009

[80] Stanghellini C, Bruins M, Mohammadkhani V, Swinkels GJ, Sonneveld PJ. Effect of condensation on light transmission and energy budget of seven greenhouse cover materials. Acta Hortic. 2012;(952):249–54. doi: 10.17660/actahortic.2012.952.30

[81] Katzin D, Marcelis LFM, van Henten EJ, van Mourik S. Heating greenhouses by light: A novel concept for intensive greenhouse production. Biosyst Eng. 2023;230:242–76. doi: 10.1016/j.biosystemseng.2023.04.003

[82] Mitchell LE, Lin JC, Bowling DR, Pataki DE, Strong C, Schauer AJ, et al. Long-term urban carbon dioxide observations reveal spatial and temporal dynamics related to urban characteristics and growth. Proc Natl Acad Sci U S A. 2018;115(12):2912–7. doi: 10.1073/pnas.1702393115 29507190 PMC5866532

[83] Zuo W, Wetter M, Li D, Jin M, Tian W, Chen Q. Coupled simulation of indoor environment, HVAC and control system by using Fast Fluid Dynamics and the Modelica Buildings library. Proceedings of ASHRAE/IBPSA-USA. Atlanta, USA: ASHRAE/IBPSA-USA; 2014. p. 56–63. Available from: https://publications.ibpsa.org/conference/paper/?id=simbuild2014_08

