# Supplementary material for: Contributions to the development and simulations of generic, modular and multiphysics greenhouses dynamic models, evaluated with a whole year study case dataset
Source: PLoS One. 2026 Feb 17;21(2):e0340619. doi: 10.1371/journal.pone.0340619 (PMC12912604; doi:10.1371/journal.pone.0340619)
Supplement: S1 File — This file includes additional details about the modelled greenhouse compartment, and provides the corresponding parameter values and related explanations. (DOCX) [file pone.0340619.s001.docx]

Supporting Information S1

-

Modelled greenhouse compartment details and model parametrization

Table of contents

[1. Greenhouse compartment overview 2](#_Toc191644442)

[2. Nantes climate compared with Almeria and De Bilt ones 2](#_Toc191644443)

[3. Validation of the implementation of Bot’s model for the calculation of the direct transmittance through a Venlo greenhouse roof 3](#_Toc191644444)

[4. Model parametrization 4](#_Toc191644445)

[4.1. Floor 4](#_Toc191644446)

[4.2. Soil 6](#_Toc191644447)

[4.3. Air volumes 8](#_Toc191644448)

[4.4. Roof 8](#_Toc191644449)

[4.4.1. Roof structure 8](#_Toc191644450)

[4.4.2. Glass properties 9](#_Toc191644451)

[4.4.3. Ventilation through the roof 9](#_Toc191644452)

[4.4.4. Other parameters 10](#_Toc191644453)

[4.5. Corridors 10](#_Toc191644454)

[4.6. Growing gutters 11](#_Toc191644455)

[4.7. Air mixing ducts 11](#_Toc191644456)

[4.8. Irradiance transmission in the greenhouse 11](#_Toc191644457)

[4.9. Horizontal screens system 13](#_Toc191644458)

[4.10. Heating pipes characteristics 14](#_Toc191644459)

[4.11. South wall 14](#_Toc191644460)

[4.12. Thermal loads 15](#_Toc191644461)

[4.13. Biological sub-models 16](#_Toc191644462)

[4.13.1. Tomato crop yield 16](#_Toc191644463)

[4.13.2. Other biological models parameters 17](#_Toc191644464)

[4.14. Thermal radiation 17](#_Toc191644465)

[4.15. Other parameters 19](#_Toc191644466)

[5. Convective heat transfer correlations 20](#_Toc191644467)

[6. References 21](#_Toc191644468)

# Greenhouse compartment overview


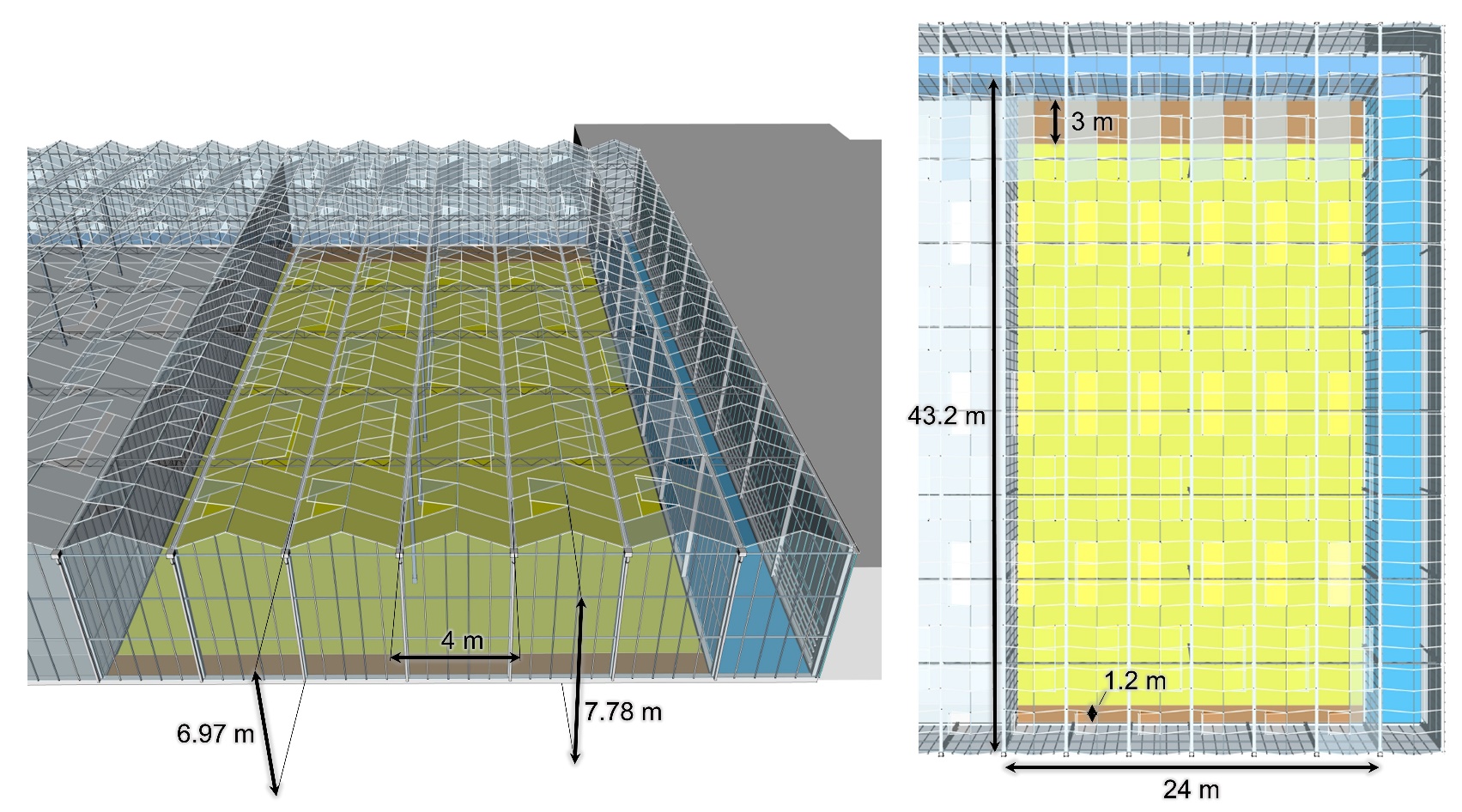
The figure below provides a general view (from the South) of the greenhouse compartment. The cultivated area is in yellow and the north and south alleyways in brown. Outside the compartment, the east and north corridors are in blue.

Fig. 1 Greenhouse compartment overview (from the south)

# Nantes climate compared with Almeria and De Bilt ones

Fig. 2 shows a comparison between the recorded insolation and temperature records and the Typical Mean Year data in Almeria (Spain) and De Bilt (The Netherlands) using PVGIS data [1,2]:


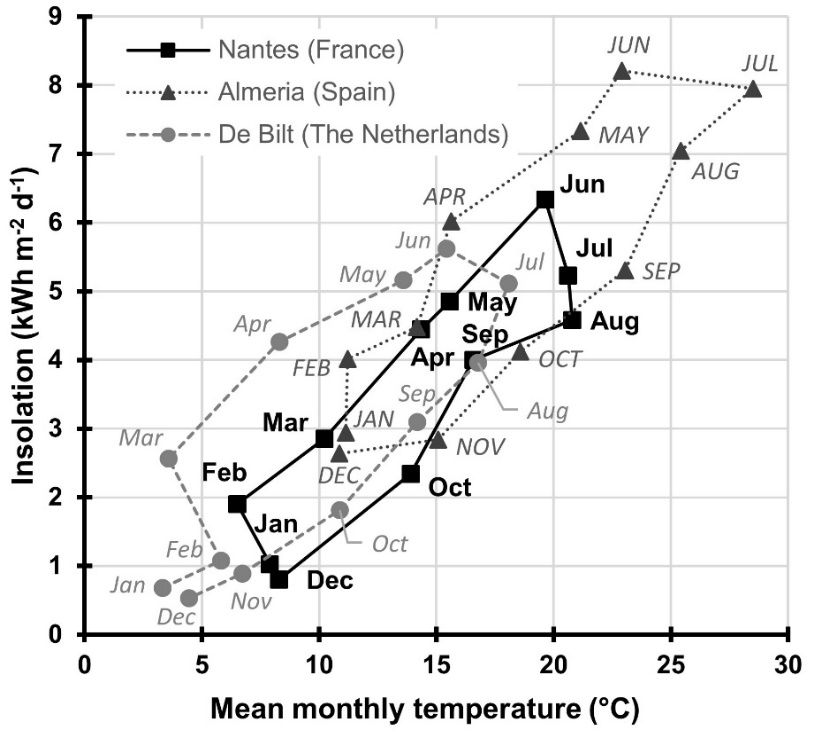


Fig. 2 Outdoor climate comparison between the measurements close to Nantes (December 2014 to October 2015) and the Typical Mean Year data [1,2] of Almeria and De Bilt.

# Validation of the implementation of Bot’s model for the calculation of the direct transmittance through a Venlo greenhouse roof

The sub-model outputs have been checked through the reproduction of Bot’s [3] results for N-S (North-South) and E-W (East-West) oriented greenhouses (52° latitude North, with common structure properties) at various days of the year. Fig. 3 shows the evolutions of $\tau_{r,dir}$, $\tau_{g}$, $\tau_{rg}$ and $\tau_{b}$ on a 21^st^ June depending on the hour of the day. The curves analysis is not reported here since it has been discussed in details by [3].

*
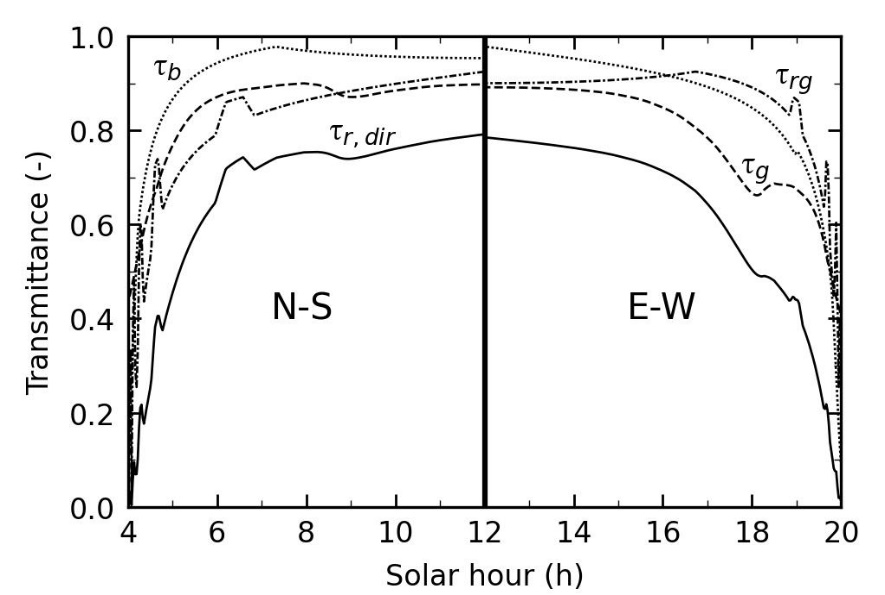
*

Fig. 3 Evolution of the global direct transmittance through a Venlo greenhouse roof $\boldsymbol{\tau}_{\boldsymbol{r}\mathbf{,}\boldsymbol{dir}}$ oriented N-S (half-left) and E-W (half right) and located at a 52° latitude (North) a 21st June. $\boldsymbol{\tau}_{\boldsymbol{g}}$ is the glass panes transmittance, $\boldsymbol{\tau}_{\boldsymbol{rg}}$ the ridges and gutters system one and $\boldsymbol{\tau}_{\boldsymbol{b}}$ the glazing bars one.

# Model parametrization

This section aims at providing the relevant sub-models parameters values that have been considered for the present work. Parameters used by several sub-models are mentioned only once. Numerical and initialization parameters are not detailed.

## Floor

| Parameter | Considered value | References and comments |
| --- | --- | --- |
| Floor surface | $1037 m^{2}$ |  |
| Floor equivalent density | $1700 kg\cdot m^{-3}$ | Below the cultivated area ($944 m^{2}$), the floor is made of dense sand/gravel. The north alleyway ($72 m^{2}$) is in concrete, as well as the south one ($<30 m^{2}$, concrete partially covered with teardrop pattern steel sheets). The weighted average is considered.   - Concrete: $2100 kg\cdot m^{-3}$ considered, based on the range noticed in the literature  \| Value \| Reference \| Application \| \| --- \| --- \| --- \| \| $1800\to2450 kg\cdot m^{-3}$ \| [4 Chap. 2.1] \| Generic \| \| $1900\to2300 kg\cdot m^{-3}$ \| [5 Table 2] \| Greenhouse dryer \| \| $2100 kg\cdot m^{-3}$ \| [6 Table B 10] \| Generic \| \| $2100 kg\cdot m^{-3}$ \| [7 Table 1] \| Greenhouse \| \| $2200 kg\cdot m^{-3}$ \| [8 Table 1] \| Greenhouse \| \| $2300 kg\cdot m^{-3}$ \| [9 Table 11] \| Greenhouse \| \| $2400 kg\cdot m^{-3}$ \| [10 para. 4.3] \| Greenhouse \|  - Cultivated area floor: $1650 kg\cdot m^{-3}$considered, based on the range noticed in the literature  \| Value \| Reference \| Application \| \| --- \| --- \| --- \| \| $1620 kg\cdot m^{-3}$ \| [11 Table B.1] \| Greenhouse \| \| $1620 kg\cdot m^{-3}$ \| [12 para 2.1] \| Greenhouse \| \| Gravel : $1674 kg\cdot m^{-3}$  Sand : $1640 kg\cdot m^{-3}$ \| [5 Table 2] \| Greenhouse dryer \| \| Ground, coarse gravel: $2040 kg\cdot m^{-3}$  Sandy ground, dry: $1650 kg\cdot m^{-3}$  Sandy ground, damp: $1750 kg\cdot m^{-3}$ \| [6 Table B 10] \| Generic \| |
| Floor equivalent specific heat capacity | $1300 J\cdot kg^{-1}\cdot K^{-1}$ | The weighted average between a dense sand/gravel and concrete is considered:   - Concrete: $880 J\cdot kg^{-1}\cdot K^{-1}$ considered  \| Value \| Reference \| Application \| \| --- \| --- \| --- \| \| $880 J\cdot kg^{-1}\cdot K^{-1}$ \| [6 Table B 10] \| Generic \| \| $880 J\cdot kg^{-1}\cdot K^{-1}$ \| [5 Table 2] \| Greenhouse dryer \| \| $880 J\cdot kg^{-1}\cdot K^{-1}$ \| [7 Table 1] \| Greenhouse \| \| $880 J\cdot kg^{-1}\cdot K^{-1}$ \| [9 Table 11] \| Greenhouse \| \| $1000 J\cdot kg^{-1}\cdot K^{-1}$ \| [8 Table 1] \| Greenhouse \|  - Cultivated area floor: $1350 J\cdot kg^{-1}\cdot K^{-1}$ considered  \| Value \| Reference \| Comment \| \| --- \| --- \| --- \| \| Gravel : $881 J\cdot kg^{-1}\cdot K^{-1}$  Sand : $830 J\cdot kg^{-1}\cdot K^{-1}$ \| [5 Table 2] \| Greenhouse dryer \| \| Ground, coarse gravel: $1840 J\cdot kg^{-1}\cdot K^{-1}$  Sandy ground, dry:$800 J\cdot kg^{-1}\cdot K^{-1}$  Sandy ground, damp: $1000 J\cdot kg^{-1}\cdot K^{-1}$ \| [6 Table B 10] \| Generic \| \| $1350 J\cdot kg^{-1}\cdot K^{-1}$ \| [13 Table 1] \| For the first layer of the Venlo greenhouse soil \| \| $1480 J\cdot kg^{-1}\cdot K^{-1}$ \| [11 Table B.1] \| Greenhouse \| \| $1480 J\cdot kg^{-1}\cdot K^{-1}$ \| [8 Table 1] \| Greenhouse \| |
| Floor thickness | $1\cdot{10}^{-2} m$ | Equivalent thickness of the resulting floor layer (alleyways + cultivated area), to reproduce the temperature gradient at the top of the floor. [14 para 5.5.1] considers a 1 cm value, while [15 Table 8.2] considers a value of 2 cm. |

Note 1: a specific heat capacity of $1300 J\cdot kg^{-1}\cdot K^{-1}$ and a density of $1700 kg\cdot m^{-3}$ lead to a volumetric heat capacity of ${2.21.10}^{6} J\cdot m^{-3}\cdot K^{-1}$.

Note 2: because of the presence of a white plastic sheet over the floor, no water mass transfer is modelled through the latter.

## Soil

| Parameter | Considered value | References and comments |
| --- | --- | --- |
| Number of layers (2 parameters) | 5 and 5 | The layer-by-layer thermal conductivity model implies the determination of the number of “virtual” layers into which the soil shall be discretised for the calculation of the conductive heat transfer.   \| Total number of layers \| Reference \| Thicknesses (cm) \| \| --- \| --- \| --- \| \| 3 \| [16 Table 1] \| 1 \| 15 \| 30 \| \| 4 \| [13 Table 1,17 Table 1] \| 5 \| 15 \| 30 \| 70 \| \| 5 \| [15 para 2.3.2.2.1] \| 4 \| 8 \| 16 \| 32 \| 64 \| \| 6 \| [12 para 1.1] \| 1 \| 4 \| 10 \| 10 \| 10 \| 30 \| \| 7 \| [14 para 5.5.1] considers 2 layers of concrete and 5 layers of soil (70% sand, 20% water and 10% air). The 1^st^ layer of concrete corresponds to the Floor one. \| 1 \| 2 \| 4 \| 8 \| 16 \| 32 \| 64 \|   In accordance with the specificities of the ground at the experimental greenhouse location (Nantes, France), the first 60 cm of soil are considered as quite homogeneous. Consequently, with a model where the layer thicknesses are doubled from the top to the bottom, 5 virtual layers are applied to the first soil physical layer (2 \| 4 \| 8 \| 16 \| 32 cm thickness).  The choice of the number of virtual layers for the second physical soil layer is driven by the choice of the depth where the soil temperature can be fixed at a constant value. This value is noticeably variable in the literature:   - According to [18 para 4.3], the temperature is constant below 3 to 4 m. - According to [19], soil temperature in Canada (where the climate variations are globally higher than in Nantes, France) is generally constant below 5 to 6 m. - According to [20], the ground temperature can still fluctuate from more than 1°C at 6 m below the surface. - According to [21], the soil temperature is undisturbed below 8 m. - For their year-round greenhouse dynamic model, [13 Table 1] consider a subsoil layer thickness of 8.8 m.   Assuming a 8 m depth as a minimum and taking into account the first 63 cm thick physical layer (1 + 2 + 4 + 8 + 16 + 32) leads to 4 virtual layers for the second physical one (64 + 128 + 256 + 512 cm). However, as discussed below concerning the soil undisturbed temperature, a 5^th^ one is required. |
| Soil undisturbed temperature | $18.1^{\circ}C$ | According to [21], the yearly average ground surface temperature should not be assumed equal to the yearly average air one, at least in the context of ground heat exchanger. Experimentally, [22] observes for instance a difference lower than 2.7 °C for a bare soil. Using the simplified correlation from [21 Eq. 5]:   \| $T_{ground,undisturbed}=17.898+0.951\cdot T_{amb}$ \| (1) \| \| --- \| --- \| |
|  |  | With $T_{ground,undisturbed}$ and $T_{amb}$ in $K$ the ground undisturbed temperature and the air average one. According to the meteorological data, the average air temperature in Nantes is close to 12.7°C, leading to a ground undisturbed temperature of 16.6°C.   \| Value \| Reference \| \| --- \| --- \| \| $12.05 ^{\circ}C$ \| Source: Drias-Climat, https://www.drias-climat.fr/decouverte/, consulted the 23/11/2023. Observation period for the reference: 1975-2005. \| \| $12.7 ^{\circ}C$ \| Source : InfoClimat, https://www.infoclimat.fr/climatologie/normales-records/1991-2020/nantes-atlantique/valeurs/07222.html, consulted the 23/11/2023. Average between 1991 and 2020 \| \| Between $12 ^{\circ}C$ to $14 ^{\circ}C$ \| Source : Meteo-France, https://meteofrance.com/comprendre-climat/france/le-climat-en-france-metropolitaine, consulted the 23/11/2023. Observation period: 1981-2010. \|   However, this value does not take into account the presence of the CTIFL facilities, which could locally affect the ground undisturbed temperature because of its land use and the average indoor temperature (20 °C over the 11 months of the measurement data). Considering a perimeter of approximately 420 m for these facilities and denoting $V$ the ground volume delimited by this perimeter, the lateral surface of $V$ equals its horizontal (top) one at approximately 16 m deep. A rough hypothesis consists in considering an isotropic volume: at 16 m deep, the equivalent soil undisturbed temperature corresponds to the average between 16.6 °C (ground undisturbed temperature, far from any building) and 20 °C (average indoor air temperature in the greenhouse facilities), i.e. 18.1 °C. This imply to consider 5 virtual layers for the second physical one. |
| Thermal conductivity of the first physical soil layer | $1 W.m^{-1}.K^{-1}$ | The weighted average between a dense sand/gravel and concrete is considered:   - Concrete:  \| Value \| Reference \| Application \| \| --- \| --- \| --- \| \| $1 W\cdot m^{-1}\cdot K^{-1}$ \| [6 Table B 10] \| Generic \| \| $1.7 W\cdot m^{-1}\cdot K^{-1}$ \| [14 para 5.5.2.2], also used by [15 Table 8.2] and [23 para 3.C.1] \| Greenhouse \| \| $1.4 W\cdot m^{-1}\cdot K^{-1}$ \| [7 Table 1] \| Greenhouse \| \| $1.5 W\cdot m^{-1}\cdot K^{-1}$ \| [8 Table 1] \| Greenhouse \|  - Soil:  \| Value \| Reference \| Application \| \| --- \| --- \| --- \| \| Ground, coarse gravel: $0.52 W\cdot m^{-1}\cdot K^{-1}$  Sandy ground, dry: $0.27 W\cdot m^{-1}\cdot K^{-1}$  Sandy ground, damp: $0.58 W\cdot m^{-1}\cdot K^{-1}$ \| [6 Table B 10] \| Generic \| \| $0.7 W\cdot m^{-1}\cdot K^{-1}$ \| [10 Table 7.3] \| Greenhouse (calibration) \| \| $0.85 W\cdot m^{-1}\cdot K^{-1}$ \| [14 para 5.5.2.2], also used by [15 Table 8.2] and [23 para 3.C.1] \| Greenhouse \| \| $1.3 W\cdot m^{-1}\cdot K^{-1}$ \| [8 Table 1] \| Greenhouse (calibration) \| \| $1.3 W\cdot m^{-1}\cdot K^{-1}$ \| [11 Table B.1] \| Greenhouse \| \| $1.4 W\cdot m^{-1}\cdot K^{-1}$ \| [24 Table 1] \| Greenhouse \| \| $1.9\to1.95 W\cdot m^{-1}\cdot K^{-1}$ \| [13 Table 1,17 Table 1] \| Greenhouse \| \| $2 W\cdot m^{-1}\cdot K^{-1}$ \| [7 Table 1] \| Greenhouse \| \| $2\to2.5 W\cdot m^{-1}\cdot K^{-1}$ \| [16 Table 1], \| Greenhouse \|   For the present experimental soil-less Venlo greenhouse, it is assumed that the upper layer ground earthworks is closer to the cases of [11,14,15,23] than to bare ground. This would lead to a thermal conductivity close to $1 W\cdot m^{-1}\cdot K^{-1}$ considering the concrete alleyways. |
| Thermal conductivity of the second physical soil layer (below 63 cm from the surface) | $2.2 W\cdot m^{-1}\cdot K^{-1}$ | In addition to the previously mentioned soil values, the EnergyPlus 9.5.0 documentation [25 para 19.6] quotes some values retrieved from the ASHRAE’s Ground Source Heat Pumps–Design of Geothermal Systems for Commercial and Institutional Buildings, 1997 and the ASHRAE Application Handbook, 2015:   \| Value \| Soil type \| \| --- \| --- \| \| $1.38 W\cdot m^{-1}\cdot K^{-1}$ \| Sand, dry density of 1281 $kg\cdot m^{-3}$, 5 % of moisture content \| \| $1.56 W\cdot m^{-1}\cdot K^{-1}$ \| Sand, dry density of 1281 $kg\cdot m^{-3}$, 15 % of moisture content \| \| $2.6 W\cdot m^{-1}\cdot K^{-1}$ \| Heavy sand, dry density of 1922 $kg\cdot m^{-3}$, 5 % of moisture content \| \| $2.77 W\cdot m^{-1}\cdot K^{-1}$ \| Coarse, dry density of 1922 $kg\cdot m^{-3}$, 5 % of moisture content \| \| $3.1 W\cdot m^{-1}\cdot K^{-1}$ \| Heavy sand, dry density of 1922 $kg\cdot m^{-3}$, 15 % of moisture content \| \| $3.29 W\cdot m^{-1}\cdot K^{-1}$ \| Coarse, dry density of 1922 $kg\cdot m^{-3}$, 15 % of moisture content \|   As well, [26] provides typical values for soft soil and sedimentary rocks: an equivalent conductivity of $2.2 W\cdot m^{-1}\cdot K^{-1}$ is thus applied for the present case. |
| First physical soil layer equivalent density | $1700 kg\cdot m^{-3}$ | This value has been set according to the analysis of the literature references mentioned in this document subsection. |
| Second physical soil layer equivalent density | $2400 kg\cdot m^{-3}$ | This value has been set according to the analysis of the literature references mentioned in this document subsection. |
| First physical soil layer equivalent specific heat capacity | $1300 J\cdot kg^{-1}\cdot K^{-1}$ | This value has been set according to the analysis of the literature references mentioned in this document subsection. This leads to a volumetric heat capacity of ${2.21.10}^{6}J\cdot m^{-3}\cdot K^{-1}$. |
| Second physical soil layer equivalent specific heat capacity | $920 J\cdot kg^{-1}\cdot K^{-1}$ | This value has been set according to the analysis of the literature references mentioned in this document subsection. This leads to the same volumetric heat capacity of ${2.21.10}^{6} J\cdot m^{-3}\cdot K^{-1}$. |

## Air volumes

| Parameter | Considered value | Comments |
| --- | --- | --- |
| Average MainAir volume height | $6.67 m$ |  |
| Average TopAir volume height | $0.7 m$ |  |

## Roof

### Roof structure

| Parameter | Considered value | Comments |
| --- | --- | --- |
| Greenhouse axis azimuth | $-29^{\circ}$ | Angle between the N-S axis and the spans axis. |
| Roof slope | $22^{\circ}$ |  |
| Span width | $4 m$ |  |
| Gutter height | $0.155 m$ | Greenhouse manufacturer data. |
| Gutter width | $0.105 m$ | Greenhouse manufacturer data. |
| Ridge height | $0.035 m$ | Greenhouse manufacturer data. |
| Ridge width | $0.040 m$ | Greenhouse manufacturer data. |
| Glazing bar height | $0.052 m$ | Greenhouse manufacturer data. |
| Glazing bar width | $0.022 m$ | Greenhouse manufacturer data. |
| Horizontal distance between two consecutive glazing bars as defined in [3 Fig. 5.15] | $1.35 m$ | Greenhouse manufacturer data. |

### Glass properties

| Parameter | Considered value | Comments |
| --- | --- | --- |
| Density | $2500 kg\cdot m^{-3}$ | The roof glass is a FLOAT security 90+ one, similar to the reference in [27] i.e. with a density of $2500 kg\cdot m^{-3}$. |
| Specific heat capacity | $840 J\cdot kg^{-1}\cdot K^{-1}$ | According [8 Table 1,11 Table B.1,14 para 5.5.1,15 Table 8.2] |
| Glass pane thickness | ${4\cdot10}^{-3} m$ |  |
| Glass transmittance (energy transmittance) | $0.9 (-)$ | According to the glass datasheet [27], the light transmittance is 0.91 but the energy transmittance equals 0.90. Consequently, it did not appear necessary to the authors to discretize the wavelength bands. |
| Glass reflectance (energy reflectance) | $0.08 (-)$ | According to the glass datasheet [27], the energy reflectance equals 0.08.The glass being symmetrical, the same value is considered in this study for upwards and downwards irradiances. |

### Ventilation through the roof

The roof is fitted with (refer to Fig. 1):

- East-facing: 24 vents of 4.05 m x 1.4 m
- West-facing: 18 vents of 4.05 m x 1.4 m and 12 vents of 1.35 m x 1.4 m

| Parameter | Considered value | Comments |
| --- | --- | --- |
| Roof window length | $4.05 m$ | Although ventilation functions depend on the vents size [3,28], the 12 smaller vents (representing 8.7% of the total opening area) are considered equivalent to the main ones (at equivalent vent area). |
| Roof window width | $1.4 m$ |  |
| Number of equivalent 4.05 x 1.4 m windows per m^2^ of greenhouse on the first roof side | $2.1\cdot{10}^{-2} window.m_{floor}^{-2}$ | West-facing vents. |
| Number of equivalent 4.05 x 1.4 m windows per m^2^ of greenhouse on the second roof side | $2.3\cdot{10}^{-2} window.m_{floor}^{-2}$ | East-facing vents. |
| Discharge coefficient through the roof openings | $0.6 (-)$ | According to [28 Table 6.1]. |
| Equivalent vents opening height for leakage calculation ($h_{vent,leak}$) | ${6\cdot10}^{-3} m$ | It defines the vent opening height (related with the opening angle and the roof slope by Bot’s equation 3.16 [3]) at which the vent is kept slightly opened (in the model) when it is supposed to be closed. This value has been found by trial and error to fit the air leakages measurements of a similar compartment by [11]. |
| Roof equivalent height | $7.4 m$ | At the roof level (7.4 m on average), the outdoor air source absolute pressure is computed taking into account the corresponding air column weight: as a simplification, the outdoor air density is approximated as a linear function of the air dry bulb temperature. |

The pressure balance between TopAir and outdoor is computed using a power law adapted from [29 chap. 16.15,30 Eq. V.1] and from the Modelica Buildings library *Orifice* component [31] (*Buildings.Airflow.Multizone.Orifice*):

| $F=sign\left( \Delta P \right)\cdot\frac{k}{A}\cdot\left\vert\Delta P \right\vert^{n}$ | (2) |
| --- | --- |

with $F$ the resulting airflow ($m^{3}\cdot s^{-1}\cdot m_{floor}^{-2}$), $A$ the compartment area ($m^{2}$), $k$ a flow coefficient ($m^{3}\cdot s^{-1}\cdot Pa^{-n}$), $\Delta P$ the pressure difference between TopAir and outdoor, and $n$ an exponent (-).

| Parameter | Considered value | Comments |
| --- | --- | --- |
| $n$ | $0.65 (-)$ | [29 chap. 16.15] |
| $k$ | ${0.4 m}^{3}\cdot s^{-1}\cdot Pa^{-n}$ | The $k$ coefficient can be estimated considering that in the general form where $n=0.5$ [30 Eq. III.33]:   \| $F=sign\left( \Delta P \right)\cdot C_{f}\cdot A_{leak}\cdot\sqrt{\frac{2\cdot\left\vert\Delta P \right\vert}{\varrho}}$ \| (3) \| \| --- \| --- \|   With $C_{f}$ the discharge coefficient (-), $A_{leak}$ the leakage area ($m^{2}$) and $\varrho$ the air density ($kg\cdot m^{-3}$). Considering:   - $C_{f}=0.6$ (-) - An average air density of $\varrho=1.2 kg\cdot m^{-3}$ - A roughly estimated leakage area of 1 mm along the join between all the vents and the roof (32 vents of 10.9 m perimeter and 12 vents of 5.5 m perimeter), i.e. 0.52 m^2^. |
|  |  | It leads to ${k=0.4 m}^{3}\cdot s^{-1}\cdot Pa^{-n}$. |

### Other parameters

| Parameter | Considered value | Comments |
| --- | --- | --- |
| Part of the solar irradiance being absorbed by the roof glazing, ridges, gutters and glazing bars | $0.04 (-)$ | \| Value \| Reference \| \| --- \| --- \| \| $0.04 (-)$ \| [14 para 5.5.2.4] found a value of 0.04 both for the direct irradiance (slightly affected by the solar position in the sky) and the diffuse one. \| \| $0.04 (-)$ \| [17 Table 1] considered a cladding absorptance of 0.04 for the diffuse irradiance. \|   A perspective would be to use a light rendering software. |

## Corridors

The airflow through the corridors walls are modelled using Eq. (2):

| Parameter | Considered value | Comments |
| --- | --- | --- |
| $n$ | $0.65 (-)$ | [29 chap. 16.15] |
| $k$ | $0.13 m^{3}\cdot s^{-1}\cdot Pa^{-n}$ | The k coefficient can be estimated considering Eq. (3), with:   - $C_{f}=0.6$ (-) - An average air density of $\varrho=1.2 kg\cdot m^{-3}$ - A roughly estimated leakage area of 1 mm along the corridors walls perimeters and access doors, i.e. $0.17 m^{2}$.   It leads to k = ${0.13 m}^{3}\cdot s^{-1}\cdot Pa^{-n}$. |
| Corridors equivalent height | $3.5 m$ | The pressure balance between MainAir and the corridors is computed at mid-height, taking into account the corresponding air column weight: as a simplification, the outdoor air density is approximated as a linear function of the air dry bulb temperature. |
| East corridor wall surface | $301 m^{2}$ |  |
| North corridor wall surface | $177 m^{2}$ |  |
| Corridor walls thickness | ${4\cdot10}^{-3} m$ |  |
| Corridor walls density | $2500 kg\cdot m^{-3}$ | The walls glass is similar to the reference in [32] i.e. with a density of $2500 kg\cdot m^{-3}$. |
| Corridor walls specific heat capacity | $840 J\cdot kg^{-1}\cdot K^{-1}$ | Same value as for the roof glass (para. 4.4.2). |

## Growing gutters

| Parameter | Considered value | Comments |
| --- | --- | --- |
| Height | $0.25 m$ | From a geometric point of view, the growing gutters and their growing medium are assumed similar to parallelepipeds. |
| Width | $0.30 m$ |  |
| Length | $39.2 m$ |  |
| Number of growing gutters | $15 (-)$ |  |

## Air mixing ducts

| Parameter | Considered value | Comments |
| --- | --- | --- |
| Diameter | $0.8 m$ |  |
| Length | $39.2 m$ |  |
| Number of air mixing ducts | 4 | Starting from the West side, the four mixing ducts are located below the 3^rd^, 6^th^, 10^th^ and 13^th^ rows, with a total mixing airflow in the greenhouse of $14600 m^{3}\cdot h^{-1}$. |

## Irradiance transmission in the greenhouse

| Parameter | Considered value | Comments |
| --- | --- | --- |
| Ratio of the irradiance being intercepted by the opaque elements (structure) inside the greenhouse and converted into sensible heat | $0.1 (-)$ | [14 para 5.5.2.4] considers a value of 0.1 for a Venlo greenhouse with a thermal screen and without artificial lighting. This value is re-used by [15 Table 8.2]. |
| Part of PAR irradiance over the global irradiance | 0.5 | It is common in the literature to attribute half of the global irradiance to PAR (Photosynthetically Active Radiation) and the other half to NIR (Near Infra Red) [15 Table 8.1,33–36]. This hypothesis was kept for the present study. However [37] reported at that time that under a clear sky PAR represent 50%, but under an overcast sky it can represent about 60%. The variability is intensively studied in the literature [38–40]. Some location-specific regression models exists in the literature such as for Almeria (Spain) [41], and model based on open-access solar measurements data are also investigated [42]. |
| Conversion factor from photons to electrons (applied to the global solar irradiance) | $4.59 \mu mol_{photon}\cdot J^{-1}$ | [15 Table 9.1, Eq. 9.19] considers a value of 2.3 $\mu mol_{photon}\cdot J^{-1}$ applied directly to the global irradiance, while other authors (e.g. [43 Chap 3.3]) uses a value of 4.59 – 4.6 $\mu mol_{photon}\cdot J^{-1}$ applied to the PAR irradiance. The explicit implementation in the BigLeaf R-package of [44] is considered for the present work:   \| $PPFD=I_{glob}\cdot frac_{PAR}\cdot\eta_{J to mol}$ \| (4) \| \| --- \| --- \|   With $PPFD$ the photonsynthetic photon flux density ($\mu mol\cdot m^{-2}\cdot s^{-1}$), $I_{glob}$ the total short-wave radiation (PAR + NIR) ($W\cdot m^{-2})$, $frac_{PAR}$ the fraction of solar irradiance that is PAR (default value of 0.5) and $\eta_{J to mol}$ the conversion factor from $J\cdot m^{-2}\cdot s^{-1}$ to $\mu mol_{photon}\cdot m^{-2}\cdot s^{-1}$ (default value of 4.6). |
| Diffuse PAR transmittance for the upwards irradiance (reflection by the screens, the canopy and the floor) | $0.75 (-)$ | The downwards solar irradiance is computed by the dedicated sub-models, with a distinction between direct and diffuse parts. In the present work, it is assumed that the reflection by all the internal surfaces to the sky through the roof is diffuse and homogeneous. The transmittance value applied to the downwards irradiance is equivalent to the upwards transmittance of the roof under an uniform overcast sky, considering:   - The horizontal symmetry of a Venlo-type roof - The fact that for the present experimental greenhouse, the glass properties are symmetrical.   Under a uniform overcast sky, the diffuse transmittance of the experimental greenhouse roof is 0.75. |
| Diffuse NIR transmittance for the upwards irradiance (reflection by the screens, the canopy and the floor) | $0.75 (-)$ | Same as for PAR. |
| Optical properties of the horizontal thermal screen (8 parameters) | See tables | Table 1 - properties of the horizontal screens (provided by Svensson, personal communication)  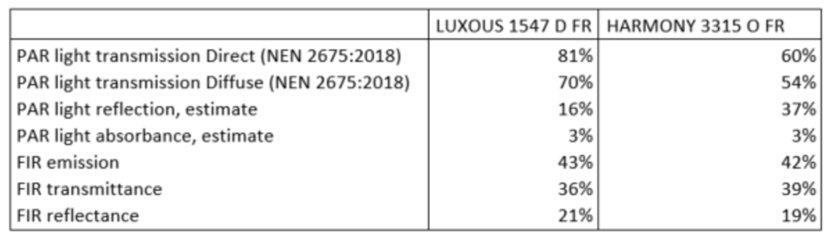   - PAR:  \| Parameter \| Value \| Comments \| \| --- \| --- \| --- \| \| Upwards transmittance \| $0.70 (-)$ \| Reflections from the greenhouse internal surface are assumed diffuse. \| \| Downwards transmittance \| $0.70 (-)$ \| The value for diffuse light is considered since the shading screen (above the thermal one) is always unfolded when the thermal one is unfolded, and since the shading screen tends to diffuse the light. However, the thermal screen is unfolded only at night (and during the very beginning of winter mornings), so this parameter is not expected to have an impact. \| \| Upwards reflectance \| $0.25 (-)$ \| The value ($0.16$) provided in Table 1 is valid for direct light. Scaling the sum of the absorbance and the reflectance to $(1-0.70)$ leads to a value of $0.047$ for the absorbance (similar to $0.05$ considered by Vanthoor [15 Table 8.2]) and $0.25$ for the reflectance. \| \| Downwards reflectance \| $0.25 (-)$ \| Same as for the upwards case. \|  - NIR: same as for PAR, as [15] has assumed. |
| Optical properties of the horizontal shading screen (8 parameters) | See tables | See Table 1.   - PAR:  \| Parameter \| Value \| Comments \| \| --- \| --- \| --- \| \| Upwards transmittance \| $0.54 (-)$ \| Reflections from the greenhouse internal surface are assumed diffuse. \| \| Downwards transmittance \| $0.57 (-)$ \| As a simplification, the average of the direct/diffuse values are considered. A more detailed approach (modulation of the transmittance depending on the direct/diffuse solar irradiance) could be applied, but this was not deemed necessary for the present study. \| \| Upwards reflectance \| $0.42 (-)$ \| The value (0.37) provided in Table 1 is valid for direct light. A scaling similar to the thermal screen case is applied. \| \| Downwards reflectance \| $0.40 (-)$ \| A scaling is applied to take into account the average transmittance. \|  - NIR: same as for PAR, as Vanthoor [15] has assumed. |
| Cultivated area ratio ($r_{cul}$) | $0.91 (-)$ | Ratio of the cultivated area over the whole greenhouse compartment area. |
| Floor reflection coefficients (2 parameters) | $0.65 (-)$ for PAR, $0.5 (-)$ for NIR | \| Value \| Reference \| \| --- \| --- \| \| $0.55 (-)$ (VIS) \| [45 para 3.2.6] mentioned a reflectance of 0.55 for white plastic sheets after some use in the visible range (VIS) \| \| $0.58 (-)$ (PAR + NIR) \| [23 Table 2.3] \| \| $0.65 (-)$ for PAR, $0.5 (-)$ for NIR \| [15 Table 8.2] \| \| $0.7 \left( - \right)$ (for PAR + NIR) \| [16 Table 1] \| \| $0.75 \left( - \right)$ for VIS, $0.40 (-)$ for NIR \| [14 chap 5.5.2.4] \| \| $0.8 (-)$ \| [11] \|   Vanthoor’s values are considered, assuming they are representative of the cultivated area (white plastic sheet, 91%), the north alleyway (concrete, 7%) and the south one (concrete and teardrop pattern steel sheets < 3%).  For the present study case, no measurements were done in 2014-2015. For prospective studies or new measurements campaigns, radiometric properties can be asked to the manufacturer, measured on site and the aging quantified. |
| Canopy extinction coefficients (4 parameters) | $0.7 (-)$ for PAR, $0.27 (-)$ for NIR | 4 parameters are theoretically required:   - 1 PAR et 1 NIR extinction coefficient for a downwards irradiance ($K_{1,PAR}$ and $K_{1, NIR}$) - 1 PAR et 1 NIR extinction coefficient for an upwards irradiance ($K_{2,PAR}$ and $K_{2, NIR}$)   However, to the best knowledge of the authors greenhouses dynamic models usually do not differentiate downwards and upwards cases. Vanthoor’s values [15 Table 8.1] are kept. For the PAR case, he follows the recommended value by [46 para 3.2]. For the NIR case, he refers to the absorption of diffuse NIR of [14]. |
| Canopy reflection coefficient (2 parameters) | $0.07 (-)$ for PAR, $0.35 \left( - \right)$ for NIR | Vanthoor’s values are kept, and no differentiation is applied between upwards and downwards cases. For PAR, $0.07$ is the recommended value by [46 para 3.2]. For the NIR case, he refers to the absorption of diffuse NIR of [14]. |

## Horizontal screens system

| Parameter | Considered value | Comments |
| --- | --- | --- |
| Length of a screen when unfolded | $5.4 m$ |  |
| Vertical distance between the thermal screen and the shading one | $0.55 m$ |  |
| Leakage airflow through a screen when unfolded | ${5\cdot10}^{-4} m^{3}\cdot s^{-1}\cdot m_{floor}^{-2}$ | This is to take into account the airflow through the gaps along a screen. The value from [47 para. 2.5.2] is considered for each screen. However, on the section where both screens are unfolded, this value is divided per 2 in the model. |
| Shading screen thickness | ${2.8\cdot10}^{-4} m$ | Estimated from similar screens studied by [47]. |
| Shading screen density | $180 kg\cdot m^{-3}$ | Considering a screen weight of 0.051 kg.m^-2^ according to the manufacturer datasheet [48]. |
| Shading screen specific heat capacity | $1800 J\cdot kg^{-1}\cdot K^{-1}$ | Vanthoor’s value for a thermal screen is considered [15 Table 8.2]. |
| Shading screen permeability | ${2.66\cdot10}^{-9} m^{2}$ | Value for a Harmony 4215 O FR [8]. In the present study, the aging effect on the permeability is not considered, although it is quantified in the literature [49]. |
| Thermal screen thickness | ${3.3\cdot10}^{-4} m$ | Value for a Luxous 1347 FR LS16U measured by [47 Table 18]. |
| Thermal screen density | $155 kg\cdot m^{-3}$ | Considering a screen weight of 0.051 kg.m^-2^ according to the manufacturer datasheet [50] |
| Thermal screen specific heat capacity | $1800 J\cdot kg^{-1}\cdot K^{-1}$ | Vanthoor’s value for a thermal screen is considered [15 Table 8.2]. |
| Thermal screen permeability | ${2.2\cdot10}^{-11} m^{2}$ | Value for a Luxous 1347 FR LS16U measured by [47 Table 18]. In the present study, the aging effect on the permeability is not considered, although it is quantified in the literature [49]. |

## Heating pipes characteristics

| Parameter | Considered value | Comments |
| --- | --- | --- |
| Rails 51 pipe diameter | ${5.1\cdot10}^{-2} m$ |  |
| Forcas pipe diameter | ${3.5\cdot10}^{-2} m$ |  |
| PE pipe diameter | $2.5\cdot{10}^{-2} m$ |  |
| Number of Rail 51 loops | $14 (-)$ |  |
| Number of Forcas loops | $15 (-)$ |  |
| Number of PE loops | $15 (-)$ |  |
| Length of a Rail 51 loop | $80 m$ |  |
| Length of a Forcas loop | $80 m$ |  |
| Length of a PE loop | $80 m$ |  |

## South wall

| Parameter | Considered value | Comments |
| --- | --- | --- |
| Wall surface | $177 m^{2}$ |  |
| Wall azimuth | $-29^{\circ}$ | Greenhouse general orientation (for the sub-models dedicated to the calculation of the solar transmission and the outdoor convection, the latter being partially dependent on the wind direction compared to the wall normal). |
| Glass thickness | $4\cdot{10}^{-3} m$ |  |
| Glass density | $2500 kg\cdot m^{-3}$ | Same value as for the roof glass (para. 4.4.2). |
| Glass specific heat capacity | $840 J\cdot kg^{-1}\cdot K^{-1}$ | Same value as for the roof glass (para. 4.4.2). |
| Glass transmittance (energy transmittance) | $0.90 (-)$ | According to the glass datasheet [32]. |
| Glass reflectance (energy reflectance) | $0.08 (-)$ | According to the glass datasheet [32], the energy reflectance equals $0.08$.The glass being symmetrical, the same value is considered in this study for upwards and downwards irradiances. |
| Curtain thickness | ${3.3\cdot10}^{-4} m$ | Refer to para. 4.9 for the thermal screen. |
| Curtain density | $155 kg\cdot m^{-3}$ | Refer to para. 4.9 for the thermal screen. |
| Curtain specific heat capacity | $1800 J\cdot kg^{-1}\cdot K^{-1}$ | Refer to para. 4.9 for the thermal screen. |
| Curtain solar transmittance | $0.70 (-)$ | Refer to para. 4.9 for the thermal screen (same value for PAR and NIR). |
| Curtain solar reflectance | $0.25 (-)$ | Refer to para. 4.9 for the thermal screen (same value for PAR and NIR). |
| Air cavity height | $2.3 m$ | When the curtain is unfolded, heat and mass (water vapour) transfer between the curtain and the glass is modelled similarly to transfer through a vertical air cavity. The curtain and the glass frame being divided into several vertical sections of approximately 2.3 m high, the air cavity height corresponds to this value, not to the whole wall height.  The heat transfer model is adapted from the Modelica *Buildings* library one (*Buildings.HeatTransfer.Windows.BaseClasses.GasConvection*) which relies on TARCOG [51]. The water vapour mass transfer is added using the Lewis analogy and taking into account the possible condensation on the curtain and on the glass. |
| View factor with the sky | $0.5 (-)$ | Flat land without close obstacles. |
| Ground albedo (outside) | $0.2 \left( - \right)$ | Although the experimental greenhouse is located in a sub-urban environment, the immediate land the south wall is facing is a cultivated area. For such ground a value of 0.2 is considered [52]. Another approach could be to use widely available satellite data [53], which has the advantage of taking into consideration the evolution during the year. However, these averaging values may not reflect the local specificities (such as the fact that two walls of the same greenhouse may face grounds of different types). |
| Ground surface roughness (or friction length) | $0.5 m$ | This is to adapt the wind speed measured on the mast (8 m high) to the south wall mid-height, using the log law [54 Eq. 15, 18]:   \| $U_{wind,z}=U_{wind,ref}\cdot\frac{ln(\frac{z+z_{0}}{z_{0}})}{ln(\frac{z_{ref}+z_{0}}{z_{0}})}$ \| (5) \| \| --- \| --- \|   With $U_{wind,z}$ the wind speed ($m\cdot s^{-1}$) at the altitude $z$ ($m$), $U_{wind,ref}$ the wind speed ($m\cdot s^{-1}$) at the reference (measure) altitude $z_{ref}$ ($m$), and $z_{0}$ the roughness length ($m$). A 0.5 value is chosen considering the experimental greenhouse surroundings [55 Table 2]. |
| Glass dirtiness | $0.1 (-)$ | This coefficient aims at taking into account the (permanent) dirtiness on the inside of the south wall glass (dust, condensation marks). A value of 0.1 has been estimated, implying that 10% of the solar irradiance received by the glass is converted into sensible heat. |

## Thermal loads

| Parameter | Considered value | Comments |
| --- | --- | --- |
| Greenhouse structure heat transfer surface | $200 m^{2}$ | Estimated. |
| Greenhouse structure mass | $12400 kg$ | [56 Table 11] stated a 7217 kg weight for the whole structure of a 605 m^2^ Venlo-type greenhouse. This value has been scaled to the present surface area. |
| Structure material specific heat capacity | $470 J\cdot kg^{-1}\cdot K^{-1}$ | [6 Table B.11,57 Table A.1]. |
| Growing gutters and growing medium heat transfer surface | $650 m^{2}$ | Estimated. |
| Growing medium mass to consider | $7960 kg$ | Estimated. |
| Growing gutters mass to consider | $6080 kg$ | Estimated. |
| Growing medium specific heat capacity | $4000 J\cdot kg^{-1}\cdot K^{-1}$ | Considering a specific heat capacity of 800 $J\cdot kg^{-1}\cdot K^{-1}$ for dry rock wool [57 Table A.2] and 4180 $J\cdot kg^{-1}\cdot K^{-1}$ for the water specific heat capacity. |
| Growing gutters specific heat capacity | $470 J\cdot kg^{-1}\cdot K^{-1}$ | [6 Table B.11,57 Table A.1] |
| Air blowers (sensible) heat load (total for the 4 fans) | $500 W$ | Based on the fans characteristics (full time operating). |

## Biological sub-models

### Tomato crop yield

The Tomato Yield Model and its parameters being described in [15], they are not detailed in this document. The table hereafter provides the values that have been considered for this study:

| Parameter | Unit | Value [15] | Value (this study) | Comments |
| --- | --- | --- | --- | --- |
| $T_{Can}^{Min}$ | $^{\circ}C$ | $10$ | $12.5$ | Fixed using parameter sweeping and breeder information |
| $T_{Can}^{Max}$ | $^{\circ}C$ | $34$ | $30$ | Fixed using parameter sweeping and breeder information |
| $T_{Can24}^{Min}$ | $^{\circ}C$ | $15$ | $16$ | Fixed using parameter sweeping and breeder information |
| $T_{Can24}^{Max}$ | $^{\circ}C$ | $24.5$ | $25.5$ | Fixed using parameter sweeping and breeder information |
| $T_{End}^{Sum}$ | $^{\circ}C$ | $1035$ | $1000$ | Fixed based on the measurements (first flowering and harvest dates, average air temperature in the greenhouse) |
| $C_{BufFruit_{1}}^{Max}$ | $fruit.plant^{-1}.s^{-1}$ | $-1.71\cdot{10}^{-7}$ | $-7.42\cdot{10}^{-7}$ | Cultivar-dependant. The parameter sweeping revealed that the value was expected to be close to the coefficient $a_{5}$ of [58], considering the applied practice (5 fruits per truss):  $C_{BufFruit_{1}}^{Max}=-k\cdot\frac{1.35\cdot{10}^{-2}}{86400}\cdot5$  $k$ being a sweeping parameter for which a value of 0.95 appeared to be suitable. |
| $C_{BufFruit_{2}}^{Max}$ | $fruit.plant^{-1}.s^{-1}.^{\circ}C^{-1}$ | $7.31\cdot{10}^{-7}$ | $4.06\cdot{10}^{-7}$ | Non cultivar dependant according to [58]. Considering the applied practice (5 fruits per truss), this value is computed from the $b$ coefficient of [58]:  $C_{BufFruit_{2}}^{Max}=\frac{7.02\cdot{10}^{-3}}{86400}\cdot5=4.06\cdot{10}^{-7}$  Note: the value is different from Vanthoor’s since he considered 9 fruits per truss [15]. |
| $c_{Dev1}$ | $s^{-1}$ | $-7.64\cdot{10}^{-9}$ | $-1.75\cdot{10}^{-9}$ | Parameter sweeping using $a_{\ldots}$values from [58] as references. The resulting values appears to be higher than the one used by Vanthoor ($a_{3}$). |
| $c_{Dev2}$ | $s^{-1}.^{\circ}C^{-1}$ | $1.16\cdot{10}^{-8}$ | $1.16\cdot{10}^{-8}$ | Non cultivar dependant according to [58]. |
| $rg_{Fruit}$ | $mg_{CH_{2}O}.m^{-2}.s^{-1}$ | $0.328$ | $0.25$ | Computed following the methodology described by Vanthoor [15 para. 9.7.3] |
| $rg_{Leaf}$ | $mg_{CH_{2}O}.m^{-2}.s^{-1}$ | $0.095$ | $0.093$ | Computed following the methodology described by Vanthoor [15 para. 9.7.3] |
| $rg_{Stem}$ | $mg_{CH_{2}O}.m^{-2}.s^{-1}$ | $0.074$ | $0.049$ | Computed following the methodology described by Vanthoor [15 para. 9.7.3] |
| $G^{Max}$ | $mg_{CH_{2}O}.fruit^{-1}$ | $10000$ | $8000$ | Estimated based on the average fresh fruit weight, the mean fruit dry matter content and the 1.082 ratio from [58]. |
| $k_{B1}$ | $day$ | $2.44$ | $-1.96$ | This is for the $B$ coefficients used to compute the fruit growth rate, refer to Equations 9.40 of [15] and [58]. A sweeping parameter method was used. |
| $k_{B2}$ | $-$ | $0.403$ | $0.741$ |  |
| $k_{M1}$ | $day$ | $-4.93$ | $2.1$ | This is for the $M$ coefficient used to compute the fruit growth rate, refer to Equations 9.41 of [15] and [58]. A sweeping parameter method was used. |
| $k_{M2}$ | $-$ | $0.548$ | $0.318$ |  |

### Other biological models parameters

The canopy transpiration model strictly follows the Stanghellini’s one [45 para 3.5.2 Eq. 3.51], consequently its parameters values are the same. Besides, all the biological sub-models are set considering the actual cultivated area using the previously described parameter $r_{cul}$ (refer to para. 4.6).

| Parameter | Considered value | Comments |
| --- | --- | --- |
| Convective heat transfer coefficient between the leaves and the air | $5 W\cdot m^{-2}\cdot K^{-1}$ | A fixed heat exchange coefficient is used for the present study [14 Eq. 5.59] as it was implemented by [59]. However, this sub-model has been modified so that it can be configured to use the explicit calculation of the boundary layer resistance of the Stanghellini’s model [14 para 5.5.2.1 Eq. 5.57, 5.58,45 para 2.3.3 Eq. 2.53]. |
| Leaf heat capacity per square meter | $1200 J\cdot K^{1}\cdot m_{leaf}^{-2}$ | [15 Table 8.1] used Stanghellini’s one [45 para 2.1.5]. |
| Typical photosynthetic efficiency (applied on PAR) | $0.15 (-)$ | In accordance with [45 para. 2.1.4], it is expected that the maximum energy stored in the products of the photosynthesis is around 10 % of the absorbed irradiance (PAR + NIR) by the canopy. Consequently, a slightly lower value (7.5 %) is considered for the present study, leading to 0.15 applied to PAR, when the PAR ratio equals 0.5. |

## Thermal radiation

| Parameter | Considered value | Comments |
| --- | --- | --- |
| **Sky** |  |  |
| Sky emissivity | $1 (-)$ | Sky is considered as a black body. |
| **Roof** |  |  |
| Roof glass emissivity | $0.89 (-)$ | According to the glass datasheet [27]. The same value applied for both surfaces. |
| Roof glass FIR transmittance | $0 (-)$ |  |
| **Corridors** |  |  |
| Wall glass emissivity | $0.89 (-)$ | According to the glass datasheet [32]. The same value applied for both surfaces. |
| Wall glass FIR transmittance | $0 (-)$ |  |
| **South wall** |  |  |
| Wall glass emissivity | $0.89 (-)$ | According to the glass datasheet [32]. The same value applied for both surfaces. |
| Wall glass FIR transmittance | $0 (-)$ |  |
| Curtain emissivity | $0.43 (-)$ | Refer to para. 4.6 for the thermal screen. |
| Curtain FIR transmittance | $0.36 (-)$ | Refer to para. 4.6 for the thermal screen. |
| **Horizontal screens system** |  |  |
| Shading screen FIR transmittance | $0.39 (-)$ | Manufacturer (Svensson) data, refer to para. 4.6. |
| Shading screen upwards emissivity | $0.42 (-)$ | Manufacturer (Svensson) data, refer to para. 4.6. |
| Shading screen downwards emissivity | $0.42 (-)$ | Manufacturer (Svensson) data, refer to para. 4.6. |
| Thermal screen FIR transmittance | $0.36 (-)$ | Manufacturer (Svensson) data, refer to para. 4.6. |
| Thermal screen upwards emissivity | $0.43 (-)$ | Manufacturer (Svensson) data, refer to para. 4.6. |
| Thermal screen downwards emissivity | $0.43(-)$ | Manufacturer (Svensson) data, refer to para. 4.6. |
| **Canopy** |  |  |
| Canopy FIR extinction coefficient ($K_{FIR})$ | $0.94 \left( - \right)$ | [14 para D.2.1,15 Table 8.1] |
| Leaf emissivity | $1 (-)$ | [14 para. D.2.1,15 Table 8.1,45 para. 2.1.1] |
| **Heating pipes** |  |  |
| Rail 51 pipe emissivity | $0.88 (-)$ | [14 para 5.5.2.3] considers an emissivity of 0.88 for white painted heating pipe, like [15 Table 8.2]. [23 Table B.1] considers a value of 0.95. These pipes are also used as guides for trolleys: therefore, their upper surface conditions might be locally close to unpainted/slightly rusted one. However, for the present experiment the greenhouse was very recent and various technical sources [60–62] tend to confirm that a value of 0.88 is still applicable.  For prospective studies or new measurements campaigns, surfaces emissivities can be asked to manufacturer, measured and their spatial heterogeneity as well as their aging quantified. |
| Forcas pipe emissivity | $0.88 (-)$ | Same as for 51 rails pipe. |
| PE pipe emissivity | $0.95 (-)$ | [60–62] |
| **Growing gutters** |  |  |
| Growing gutters equivalent emissivity | $0.88 (-)$ | The same value as for the heating pipes is considered: the growing gutters are metallic and white-painted, and the growing medium is enclosed into a white plastic sheet. |
| **Air Mixing ducts** |  |  |
| Air mixing duct emissivity | $0.88 (-)$ | Assumed for the white plastic sheet constituent the flexible duct. |
| **Floor** |  |  |
| White plastic sheet emissivity | $0.7 (-)$ | The table below lists a few values considered in the literature for the white plastic sheets covering the cultivated area:   \| Value \| Reference \| \| --- \| --- \| \| $0.4 (-)$ \| [11 Table 3] \| \| $0.7 (-)$ \| [23 Table B.1] \| \| $0.85 (-)$ \| [16 Table 1] \| \| $1 (-)$ \| [15 Table 8.2,45 para 3.2.6] \|   As well, the value used in the literature for the concrete emissivity noticeably varies:   \| Value \| Reference \| \| --- \| --- \| \| $0.6 (-)$ \| [9 7.III.2] \| \| $0.71 (-)$ \| [7 Table 1] \| \| $0.88 (-)$ \| [8 Table 2] \| \| $0.92\to0.97 (-)$ \| [61] \| \| $0.94 (-)$ \| [6 Table B 12] \| |

## Other parameters

| Parameter | Considered value | Comments |
| --- | --- | --- |
| Outdoor air absolute pressure at the ground level | $101325 Pa$ | No outdoor air pressure measurement was done during the experiment, so a constant reference value is considered. The reference pressure at the sea level is used since:   - The local altitude (approximately 30 m) is not susceptible to significantly influence the air properties. - Only the pressure difference between a greenhouse volume and outside matters. |
| Air absolute pressure in the corridors | $101325 Pa$ | Assumed to be equal to the outdoor air pressure. |
| Lewis number (water vapour in air) | $0.89 (-)$ | The Lewis number $Le$ values considered by authors slightly differ in the literature, as illustrated below with a few examples:   \| Value \| Reference \| \| --- \| --- \| \| $0.81 (-)$ \| [63] \| \| $0.845 (-)$ \| [29 Chap 6.9 for Eq. 37] \| \| $0.86 (-)$ \| [64] \| \| $0.87 (-)$ \| [6 Table 1.5], at 0°C 100 hPa. \| \| $0.888 (-)$ \| [24] compute $Le$ as a function of the temperature based on their equations (24-2) and (24-3), but it remains close to 0.888 between 15°C and 35°C. \| \| $0.89 (-)$ \| [13 Table 1,14 App. B,17 Table 1,23 Table B.1] \|   $Le$ (-) is defined:  $Le= \frac{a}{D_{v}}$  With $a$ the thermal diffusivity ($m^{2}.s^{-1}$) and $D_{v}$ the binary mass diffusion coefficient of water vapour in air ($m^{2}\cdot s^{-1}$). $a$ can be computed:  $a= \frac{k}{\rho\cdot C_{p}}$  With $k$ the thermal conductivity ($W\cdot m^{-1}\cdot K^{-1})$, $\rho$ the density ($kg\cdot m^{-3})$ and $C_{p}$ the specific heat capacity ($J\cdot kg^{-1}\cdot K^{-1}$) of the moist air. All these terms have been computed using CoolProp [65] (www.coolprop.org) and the mass diffusion coefficient values from [29 Table 4 Chap 1.15] have been used to deduce $Le$ between 15 °C to 40 °C. It leads to an average value of 0.85, consistent with the value explicitly given in [29 Chap 6.9] (0.845). However, using [66 Eq. 4.3-2 & Table 13] for the calculation of $D_{v}$ leads to a slightly higher value, close to 0.89 for temperature and humidity ranges applicable for this study.  Since most of the papers read by the authors use a value close to 0.89 for their own studies, the latter is considered. |

# Convective heat transfer correlations

The table hereafter lists the correlations used to compute the convective heat transfer coefficients between flat surfaces and air. For prospective studies, in addition to a literature review Fast Fluid Dynamics (FFD) or Computed Fluid Dynamics (CFD) could be used to assess with more confidence the heat transfer coefficients:

| **Case** | **Correlation (**$\boldsymbol{h}_{\boldsymbol{c}}$ **in** $\boldsymbol{W.}\boldsymbol{K}^{\boldsymbol{-1}}\boldsymbol{.}\boldsymbol{m}^{\boldsymbol{-2}}$ **per m^2^ of surface).** $\boldsymbol{T}_{\boldsymbol{s}}$ **: surface temperature (K) \|** $\boldsymbol{T}_{\boldsymbol{a}}$ **: air temperature (K) \|** $\boldsymbol{\Delta T=}\boldsymbol{T}_{\boldsymbol{s}}\boldsymbol{-}\boldsymbol{T}_{\boldsymbol{a}}$ **\|** $\boldsymbol{\xi}$**: surface tilt (°)** | | | **Remark** |
| --- | --- | --- | --- | --- |
| Floor / Air  (air above the surface) | $T_{s}>T_{a}$: | $h_{c}=5.2\cdot\left\vert\Delta T \right\vert^{0.33}$ | (6) | Correlation of [67 Table 4] for a heated greenhouse floor. In the present study, it is expected that the mixing ducts below the growing gutters increase the heat transfer coefficient. That is why this correlation is kept even when the heating pipes are not working, compared to other correlations with smaller coefficients [68 Table 6]. |
|  | $T_{s}<T_{a}$: | $h_{c}=1.3\cdot\left\vert\Delta T \right\vert^{0.25}$ | (7) | Correlation kept from [14 Eq. 5.6,15 Table 8.4]. |
| Wall / Air | $\forall T_{s}$ and $T_{a}$: | $h_{c}=1.31\cdot\left\vert\Delta T \right\vert^{0.33}$ | (8) | Correlation from [25 Eq. 3.100], which is close to [69]. |
| Cover / indoor air  (tilted surface above the air volume) | $T_{s}>T_{a}$ | $h_{c}=(1.3\cdot\left\vert\Delta T \right\vert^{0.33})/\left[ 1+\left( \left\vert\Delta T \right\vert^{0.08}-1 \right)\cdot\left\vert\cos\xi\right\vert\right]$ | (9) | This is to provide a continuous function of the tilt angle, similar to [25 Eq. 3.102,70 Eq. J.2.2.b] but which corresponds to:   - the correlation n°(7) when $\xi=0^{\circ}$ (horizontal) - the correlation n°(8) when $\xi=90^{\circ}$ (vertical)   Note: the implementation shall take into account that this formula is not applicable for $\Delta T=0$ |
|  | $T_{s}<T_{a}$ | $h_{c}=(4.43\cdot\left\vert\Delta T \right\vert^{0.33}/(3.38-\left\vert\cos\xi\right\vert)$ | (10) | This is to provide a continuous function of the tilt angle, similar to [25 Eq. 3.101,70 Eq. J.2.2.a] but which corresponds to:   - When $\xi=0^{\circ}$ (horizontal), the correlation from De Halleux [71] $h_{c}=1.86\cdot\left\vert\Delta T \right\vert^{0.33}$ for large-scale greenhouse when $T_{s}>T_{a}$ and the surface is below the air volume (equivalent to the case where $T_{s}<T_{a}$ and the surface is above the air volume). - When $\xi=90^{\circ}$ (vertical), to the correlation n°(8). |
| Air / Horizontal screen  (air below the surface) | $T_{s}>T_{a}$ | Assumed to be equivalent as (7) |  |  |
|  | $T_{s}<T_{a}$ | $h_{c}=2.09\cdot\left\vert\Delta T \right\vert^{0.33}$ | (11) | From [72]. |
| Air / Horizontal screen  (air above the surface) | $T_{s}>T_{a}$ | $h_{c}=3.09\cdot\left\vert\Delta T \right\vert^{0.33}$ | (12) | From [72]. |
|  | $T_{s}<T_{a}$ | Assumed to be equivalent as (7) |  |  |

- Note concerning the heat transfer coefficient between the indoor air and a tilted roof

[72] have found for a twin-span glasshouse (22° tilt angle, span width of 4.1 m) $h_{c}=2.97\cdot\left| \Delta T \right|^{0.33}$, while (10) leads to $h_{c}=1.81\cdot\left| \Delta T \right|^{0.33}$ with the same angle. For a plastic greenhouse (45° tilt angle, span width of 4.9 m) [69] have found $h_{c}=1.9\cdot\left| \Delta T \right|^{0.33}$, while (10) leads to $h_{c}=1.67\cdot\left| \Delta T \right|^{0.33}$ with the same angle. In comparison, using [14 Eq. 5.48] leads to $h_{c}=1.52\cdot\left| \Delta T \right|^{0.33}$(for the [72] case) and $h_{c}=1.66\cdot\left| \Delta T \right|^{0.33}$ (for the [69] case).

# References

1. PVGIS. Photovoltaïc Geographical Information System - TMY. In: PVGIS - Interactive Tools - Typical Meteorological Year [Internet]. 2023 [cited 29 Aug 2023]. Available from: https://re.jrc.ec.europa.eu/pvg_tools/en/#TMY

2. Huld T, Müller R, Gambardella A. A new solar radiation database for estimating PV performance in Europe and Africa. Solar Energy. 2012;86: 1803–1815. doi: 10.1016/j.solener.2012.03.006

3. Bot GPA. Greenhouse climate from physical processes to a dynamic model. Doctoral dissertation, Wageningen University. 1983. Available from: https://edepot.wur.nl/188427

4. Herr H, Bach E, Bertrand P, Bierwerth W, Tonert M. Génie énergétique et climatique. DunodTech. 2011.

5. Srinivasan G, Muthukumar P. A review on solar greenhouse dryer: Design, thermal modelling, energy, economic and environmental aspects. Solar Energy. 2021 [cited 22 Jul 2021]. doi: 10.1016/j.solener.2021.04.058

6. Baehr HD, Stephan K. Heat and Mass Transfer. Second revised Edition. 2006.

7. Wang X, Luo J, Li X. CFD Based Study of Heterogeneous Microclimate in a Typical Chinese Greenhouse in Central China. Journal of Integrative Agriculture. 2013;12: 914–923. doi: 10.1016/S2095-3119(13)60309-3

8. Santolini E, Pulvirenti B, Guidorzi P, Bovo M, Torreggiani D, Tassinari P. Analysis of the effects of shading screens on the microclimate of greenhouses and glass facade buildings. Building and Environment. 2022;211: 108691. doi: 10.1016/j.buildenv.2021.108691

9. Morille B. Élaboration d’un modèle du climat distribué à l’échelle de l’abri et de la plante en cultures ornementales sous serres: analyse des transferts de masse et de chaleur, bilans énergétiques. Doctoral dissertation, Agrocampus Ouest. 2012. Available from: https://tel.archives-ouvertes.fr/tel-00958617

10. Piché P. Amélioration du comportement thermique d’une serre nordique communautaire. Doctoral dissertation, Université de Pau et des Pays de l’Adour. 2021. Available from: https://tel.archives-ouvertes.fr/tel-03368948

11. Boulard T, Roy J-C, Pouillard J-B, Fatnassi H, Grisey A. Modelling of micrometeorology, canopy transpiration and photosynthesis in a closed greenhouse using computational fluid dynamics. Biosystems Engineering. 2017;158: 110–133. doi: 10.1016/j.biosystemseng.2017.04.001

12. Monteil C, Amouroux M. Analyse du comportement thermique du sol d’une serre agricole par simulation dynamique. J Phys III France. 1997;7: 405–440. doi: 10.1051/jp3:1997130

13. Mashonjowa E, Ronsse F, Milford JR, Pieters JG. Modelling the thermal performance of a naturally ventilated greenhouse in Zimbabwe using a dynamic greenhouse climate model. Solar Energy. 2013;91: 381–393. doi: 10.1016/j.solener.2012.09.010

14. De Zwart HF. Analyzing energy-saving options in greenhouse cultivation using a simulation model. Doctoral dissertation, Wageningen University. 1996. Available from: https://research.wur.nl/en/publications/analyzing-energy-saving-options-in-greenhouse-cultivation-using-a-2

15. Vanthoor BHE. A model-based greenhouse design method. Doctoral dissertation, Wageningen University. 2011. Available from: https://edepot.wur.nl/170301

16. Wang S, Boulard T. Predicting the Microclimate in a Naturally Ventilated Plastic House in a Mediterranean Climate. Journal of Agricultural Engineering Research. 2000;75: 27–38. doi: 10.1006/jaer.1999.0482

17. Pieters JG, Deltour JM. Modelling solar energy input in greenhouses. Solar Energy. 1999;67: 119–130. doi: 10.1016/S0038-092X(00)00054-2

18. Sethi VP, Sumathy K, Lee C, Pal DS. Thermal modeling aspects of solar greenhouse microclimate control: A review on heating technologies. Solar Energy. 2013;96: 56–82. doi: 10.1016/j.solener.2013.06.034

19. Williams GP, Gold LW. Les températures du sol. Digeste de la construction au Canada. 1977; 7 p. doi: 10.4224/40000922

20. Guillou-Frottier L. Température du sol et variations climatiques au cours des derniers millénaires. 2004. Available from: https://planet-terre.ens-lyon.fr/ressource/profil-thermique-sol.xml

21. Ouzzane M, Eslami-Nejad P, Badache M, Aidoun Z. New correlations for the prediction of the undisturbed ground temperature. Geothermics. 2015;53: 379–384. doi: 10.1016/j.geothermics.2014.08.001

22. Mariolopoulos EG. Sur la température à la surface du sol et à différentes profondeurs à Athènes. Annales de l’Observatoire national d’Athènes. 1928;10: XXII–XXIX. Available from: http://adsabs.harvard.edu/full/1928AnAth..10D..22M

23. Van Ooteghem RJ. Optimal control design for a solar greenhouse. Doctoral dissertation, Wageningen University. 2007.

24. Liu R, Li M, Guzmán JL, Rodríguez F. A fast and practical one-dimensional transient model for greenhouse temperature and humidity. Computers and Electronics in Agriculture. 2021;186. doi: 10.1016/j.compag.2021.106186

25. EnergyPlus. EnergyPlus documentation. [Software]. US DOE; 2021. Available from: https://energyplus.net/documentation

26. Reiss C. Protocole de test de réponse thermique. Ventilone, BRGM; 2012 Mar. Report No.: BRGM/RP-60816-FR. Available from: https://infoterre.brgm.fr/rapports/RP-60816-FR.pdf

27. Saint Gobain. SECURIT DIAMANT Cahier des charges.pdf. 2022. Available from: https://befr.saint-gobain-building-glass.com/sites/saint-gobain-building-glass.com/files/documentPdf/SECURIT%20DIAMANT%20Cahier%20des%20charges.pdf

28. De Jong T. Natural ventilation of large multi-span greenhouses. Doctoral dissertation, Wageningen University. 1990. Available from: https://library.wur.nl/WebQuery/wurpubs/fulltext/206452

29. ASHRAE. 2013 ASHRAE handbook: fundamentals. SI edition. Atlanta, GA; 2013.

30. Sherman M. Air Infiltration in Buildings. Doctoral dissertation, University of California, Berkeley. 1980.

31. Wetter M, Zuo W, Nouidui TS, Pang X. Modelica Buildings library. Journal of Building Performance Simulation. 2014;7: 253–270. doi: 10.1080/19401493.2013.765506

32. Saint Gobain. DIAMANT_fiche_technique.pdf. 2022. Available from: https://befr.saint-gobain-building-glass.com/sites/saint-gobain-building-glass.com/files/documentPdf/DIAMANT_fiche_technique.pdf

33. Baeza E, Hemming S, Stanghellini C. Materials with switchable radiometric properties: Could they become the perfect greenhouse cover? Biosystems Engineering. 2020;193: 157–173. doi: 10.1016/j.biosystemseng.2020.02.012

34. Spitters CJT. Separating the diffuse and direct component of global radiation and its implications for modeling canopy photosynthesis Part II. Calculation of canopy photosynthesis. Agricultural and Forest Meteorology. 1986;38: 231–242. doi: 10.1016/0168-1923(86)90061-4

35. Stanghellini C, Dai J, Kempkes FLK. Effect of near-infrared-radiation reflective screen materials on ventilation requirement, crop transpiration and water use efficiency of a greenhouse rose crop. Biosystems Engineering. 2011;110: 261–271. doi: 10.1016/j.biosystemseng.2011.08.002

36. Montero JI, Teitel M, Baeza E, Lopez JC, Kacira M. Greenhouse design and covering materials. Good agricultural practices for greenhouse vegetable crops: principles for Mediterranean climate areas. Rome: Food and Agricultural Organization of the United Nations (FAO); 2013. Available from: https://www.fao.org/3/i3284e/i3284e.pdf

37. Goudriaan J. Crop micrometeorology: a simulation study. Doctoral dissertation, Wageningen University. 1977. Available from: https://edepot.wur.nl/166537

38. Gueymard C. An atmospheric transmittance model for the calculation of the clear sky beam, diffuse and global photosynthetically active radiation. Agricultural and Forest Meteorology. 1989;45: 215–229. doi: 10.1016/0168-1923(89)90045-2

39. Papaioannou G, Papanikolaou N, Retalis D. Relationships of photosynthetically active radiation and shortwave irradiance. Theor Appl Climatol. 1993;48: 23–27. doi: 10.1007/BF00864910

40. Yu X, Wu Z, Jiang W, Guo X. Predicting daily photosynthetically active radiation from global solar radiation in the Contiguous United States. Energy Conversion and Management. 2015;89: 71–82. doi: 10.1016/j.enconman.2014.09.038

41. Alados I, Foyo-Moreno I, Alados-Arboledas L. Photosynthetically active radiation: measurements and modelling. Agricultural and Forest Meteorology. 1996;78: 121–131. doi: 10.1016/0168-1923(95)02245-7

42. Thomas C, Wandji Nyamsi W, Arola A, Pfeifroth U, Trentmann J, Dorling S, et al. Smart Approaches for Evaluating Photosynthetically Active Radiation at Various Stations Based on MSG Prime Satellite Imagery. Atmosphere. 2023;14: 1259. doi: 10.3390/atmos14081259

43. Heuvelink E. Tomato growth and yield: quantitative analysis and synthesis. Doctoral dissertation, Wageningen University. 1996. Available from: https://edepot.wur.nl/206832

44. Knauer J, El-Madany TS, Zaehle S, Migliavacca M. Bigleaf - An R package for the calculation of physical and physiological ecosystem properties from eddy covariance data. Bond-Lamberty B, editor. PLoS ONE. 2018;13: e0201114. doi: 10.1371/journal.pone.0201114

45. Stanghellini C. Transpiration of greenhouse crops: an aid to climate management. Doctoral dissertation, Wageningen University. 1987. Available from: https://edepot.wur.nl/202121

46. Marcelis LFM, Heuvelink E, Goudriaan J. Modelling biomass production and yield of horticultural crops: a review. Scientia Horticulturae. 1998;74: 83–111. doi: 10.1016/S0304-4238(98)00083-1

47. Hemming S, Baeza E, Mohammadkhani V, Van Breugel B. Energy saving screen materials : measurement method of radiation exchange, air permeability and humidity transport and a calculation method for energy saving. Bleiswijk: Wageningen University & Research, BU Greenhouse Horticulture; 2017. doi: 10.18174/409298

48. Svensson. Harmony 3315 O FR datasheet. 2023. Available from: https://www.ludvigsvensson.com/Temp/HARMONY3315OFR_CSProductsheet_en-US.pdf

49. Miguel AF, Van De Braak NJ, Bot GPA. Analysis of the Airflow Characteristics of Greenhouse Screening Materials. Journal of Agricultural Engineering Research. 1997;67: 105–112. doi: 10.1006/jaer.1997.0157

50. Svensson. Luxous 1547 D FR datasheet. 2023. Available from: https://www.ludvigsvensson.com/Temp/LUXOUS1547DFR_CSProductsheet_en-US.pdf

51. Carli. Tarcog: Mathematical models for calculation of thermal performance of glazing systems with or without shading devices. 2006 Oct. Available from: https://windows.lbl.gov/sites/all/files/Downloads/tarcog-mathematical-model.pdf

52. Ineichen P, Guisan O, Perez R. Ground-reflected radiation and albedo. Solar Energy. 1990;44: 207–214. doi: 10.1016/0038-092X(90)90149-7

53. Copernicus. Copernicus Atmosphere Monitoring Services (CAMS) Atmosphere Data Store (ADS). In: CAMS solar radiation time-series [Internet]. 2021 [cited 17 Nov 2021]. Available from: https://ads.atmosphere.copernicus.eu/cdsapp#!/dataset/cams-solar-radiation-timeseries?tab=overview

54. Norton T, Sun D-W, Grant J, Fallon R, Dodd V. Applications of computational fluid dynamics (CFD) in the modelling and design of ventilation systems in the agricultural industry: A review. Bioresource technology. 2007;98: 2386–414. doi: 10.1016/j.biortech.2006.11.025

55. Davenport A, Grimmond C, Oke T, Wieringa J. Estimating the roughness of cities and sheltered country. 12th conference on applied climatology, Ashville, NC, American Meteorological Society. 2000; 96–99.

56. Maraveas C, Tsavdaridis KD. Strengthening Techniques for Greenhouses. AgriEngineering. 2020;2: 37–54. doi: 10.3390/agriengineering2010003

57. Lienhard V JH, Lienhard IV JH. A Heat Transfer Textbook. 5.10. Phlogiston Press Cambridge, Massachusetts, U.S.A.; 2020. Available from: https://ahtt.mit.edu/wp-content/uploads/2020/08/AHTTv510.pdf

58. De Koning ANM. Development and dry matter distribution in glasshouse tomato : a quantitative approach. Doctoral dissertation, Wageningen University. 1994. Available from: https://research.wur.nl/en/publications/development-and-dry-matter-distribution-in-glasshouse-tomato-a-qu

59. Altes-Buch Q, Quoilin S, Lemort V. Greenhouses: A Modelica Library for the Simulation of Greenhouse Climate and Energy Systems. Proceedings of the 13th International Modelica Conference. Regensburg, Germany; 2019. pp. 533–542. doi: 10.3384/ecp19157533

60. Omega. Table of total emissivity. 2022. Available from: https://assets.omega.com/pdf/tables_and_graphs/emissivity-table.pdf

61. Thermoworks. Infrared emissivity table. 22 Oct 2022 [cited 22 Oct 2022]. Available from: https://www.thermoworks.com/emissivity-table/

62. Transmetra. Table of emissivity of various surfaces. 2022. Available from: https://www.transmetra.ch/images/transmetra_pdf/publikationen_literatur/pyrometrie-thermografie/emissivity_table.pdf

63. Fatnassi H, Boulard T, Bouirden L. Development, validation and use of a dynamic model for simulate the climate conditions in a large scale greenhouse equipped with insect-proof nets. Computers and Electronics in Agriculture. 2013;98: 54–61. doi: 10.1016/j.compag.2013.07.008

64. Majdoubi H, Boulard T, Fatnassi H, Bouirden L. Airflow and microclimate patterns in a one-hectare Canary type greenhouse: An experimental and CFD assisted study. Agricultural and Forest Meteorology. 2009;149: 1050–1062. doi: 10.1016/j.agrformet.2009.01.002

65. Bell I, Wronski J, Quoilin S, Lemort V. Pure and Pseudo-pure Fluid Thermophysical Property Evaluation and the Open-Source Thermophysical Property Library CoolProp. Ind Eng Chem Res. 2014;53: 2498–2508. doi: 10.1021/ie4033999

66. Marrero TR, Mason EA. Gaseous Diffusion Coefficients. Journal of Physical and Chemical Reference Data. 1972;1: 3–118. doi: 10.1063/1.3253094

67. Lamrani MA, Boulard T, Roy J-C, Jaffrin A. SE—Structures and Environment: AirFlows and Temperature Patterns induced in a Confined Greenhouse. Journal of Agricultural Engineering Research. 2001;78: 75–88. doi: 10.1006/jaer.2000.0568

68. Roy J-C, Boulard T, Kittas C, Wang S. PA—Precision Agriculture: Convective and Ventilation Transfers in Greenhouses, Part 1: the Greenhouse considered as a Perfectly Stirred Tank. Biosystems Engineering. 2002;83: 1–20. doi: 10.1006/bioe.2002.0107

69. Piscia D, Muñoz P, Panadès C, Montero JI. A method of coupling CFD and energy balance simulations to study humidity control in unheated greenhouses. Computers and Electronics in Agriculture. 2015;115: 129–141. doi: 10.1016/j.compag.2015.05.005

70. Walton GN. Thermal Analysis Research Program reference manual. Washington, USA; 1983. Available from: https://www.govinfo.gov/content/pkg/GOVPUB-C13-6176908b08a357a0ac91a8ab3db55b97/pdf/GOVPUB-C13-6176908b08a357a0ac91a8ab3db55b97.pdf

71. De Halleux D. Dynamic model of heat and mass transfer in greenhouses: theoretical and experimental study. Doctoral dissertation, Gembloux, Belgium. 1989.

72. Miguel AF, Van De Braak NJ, Silva AM, Bot GPA. Free-Convection Heat Transfer in Screened Greenhouses. Journal of Agricultural Engineering Research. 1998;69: 133–139. doi: 10.1006/jaer.1997.0235
