# Supplementary material for: Contributions to the development and simulations of generic, modular and multiphysics greenhouses dynamic models, evaluated with a whole year study case dataset
Source: PLoS One. 2026 Feb 17;21(2):e0340619. doi: 10.1371/journal.pone.0340619 (PMC12912604; doi:10.1371/journal.pone.0340619)
Supplement: S2 File — This dataset includes all the modelled greenhouse compartment experimental inputs during the whole simulation, as well as the model outputs presented and discussed in this paper. This supporting information is available from https://doi.org/10.5281/zenodo.14943558. (DOCX) [file pone.0340619.s002.docx]

Supporting Information S2

-

**S.2 Dataset. Dataset of the global model inputs and outputs presented in this paper**. This dataset includes all the modelled greenhouse compartment experimental inputs during the whole simulation, as well as the model outputs presented and discussed in this paper. This supporting information is available from <https://doi.org/10.5281/zenodo.14943558>
