# Supplementary material for: Contributions to the development and simulations of generic, modular and multiphysics greenhouses dynamic models, evaluated with a whole year study case dataset
Source: PLoS One. 2026 Feb 17;21(2):e0340619. doi: 10.1371/journal.pone.0340619 (PMC12912604; doi:10.1371/journal.pone.0340619)
Supplement: S5 File — This supporting information is available from https://doi.org/10.5281/zenodo.15590403. (DOCX) [file pone.0340619.s005.docx]

Supporting Information S5

-

**S.5 Software. Modelica models to reproduce the paper results.** This supporting information is available from <https://doi.org/10.5281/zenodo.15590403>
